# Mapping relational mechanism clusters in *Of Human Bondage*: a theory-driven multiscale embedding analysis

**DOI:** 10.3389/fpsyg.2026.1836153

**Published:** 2026-07-16

**Authors:** Zhengting Zhao

**Affiliations:** Graduate Institute of Interpretation and Translation, Shanghai International Studies University, Shanghai, China

**Keywords:** coercive control, literary narrative, multiscale analysis, *Of Human Bondage*, relational dynamics, text embedding analysis, theory-driven construct measurement, trauma bonding

## Abstract

**Introduction:**

Some psychological patterns appear as events; others become visible only as sequences. Literary fiction is unusually rich in such sequences, because it records how attachment, injury, dependence, and release accumulate across time. This study asks whether the language of one novel—W. Somerset Maugham’s *Of Human Bondage*—is organized around specific psychological mechanisms, using theory-guided text embeddings.

**Methods:**

We wrote short “anchor” texts for eight relational or existential constructs and two negative-control domains, then measured, in embedding space, how closely each chapter and scene matched each anchor. At chapter level (122 chapters), we tested whether construct signals peak at the same chapters, whether the Mildred Arc differs from the Resolution segment, and whether neighbouring chapters resemble one another. At scene level (165 scenes from Chapters 55–122), we identified which scenes drive chapter peaks. Three embedding models tested robustness; six independent readers rated 20 scenes for external validation.

**Results:**

The construct signals clustered at the same chapters far more than chance permutations allow. In the late novel, four constructs—a narcissistic relational style, coercive control, intermittent reinforcement, and trauma bonding—were higher in the Mildred Arc than in the Resolution segment, while other constructs were not. Scene analysis showed that each chapter peak came from a few distinct local scenes rather than from uniform elevation. The negative controls did not follow the toxic-relational pattern; they peaked instead in reasoning-heavy and travel passages. The pattern held across all three models; the largest model did not give the cleanest separation. Human ratings of key scenes converged with the model on the core constructs (mean Spearman *ρ* = 0.46, individually significant for the narcissistic-style, coercive-control, trauma-bonding, helplessness, and existential signals) but not on three broader anchors.

**Discussion:**

Without diagnosing fictional characters, the study shows how theory-guided anchors can map where a novel organizes interpersonal mechanisms, combining chapter-level trajectories, scene-level localization, negative-control anchors, cross-model checks, and human-rater validation. The workflow is portable to other long-form literary texts.

## 1 Introduction

Some psychological patterns announce themselves in a single scene; others have to be followed. Coercive control, trauma bonding, intermittent reinforcement, and learned helplessness are not isolated acts but temporal formations, made legible through recurrence, delay, return, and gradual loss or recovery of agency. Prospective study of such processes is ethically fraught and practically difficult, and much evidence therefore comes from retrospective self-report or one-time snapshots; the temporal features that matter most are often easier to describe in theory than to observe directly (Herman, 1992; Seligman, 1975; Walker, 1979; Stark, 2007).

Literary fiction offers a different kind of evidence. It is not clinical truth, but it can preserve long, detailed traces of social and emotional process that standard methods miss. Such material is usually studied through close reading, which is illuminating but hard to test systematically: a close reading explains *why* a relationship seems destructive, yet rarely gives a reproducible account of *where* in the narrative those dynamics intensify, cluster, or change form. Existing computational approaches, in turn, are either too general or too static to track specific interpersonal mechanisms over time (see Section 1.2).

This study addresses that gap with a simple, theory-driven design. For each psychological construct we write short descriptive “anchor” texts, convert both the anchors and the novel’s text into *embeddings*—numerical vectors in which passages with similar meaning lie close together—and measure how closely each part of the novel matches each anchor. If the resulting pattern is non-random, organized in time, concentrated in particular scenes, and distinct from negative-control anchors, then the novel can be studied not just as a source of themes but as a structured map of interpersonal process.

### 1.1 Literary narrative as longitudinal psychological material

Using fiction for psychological analysis requires care, but it is not arbitrary. Narrative psychology has long held that stories work as simulations of social life (Mar and Oatley, 2008; Oatley, 2016; Oatley and Djikic, 2018), and recent work shows that the cues in a narrative actively guide how readers reconstruct characters’ mental states (Eekhof et al., 2021). For the present purpose, the novel is valuable because it preserves patterned representations of action, motive, attachment, and self-interpretation over spans of time that ordinary empirical study cannot easily reach. This matters most when the phenomenon is cumulative, cyclical, or hard to track ethically in real life.

*Of Human Bondage* is particularly valuable in this respect. Long recognized as a novel with autobiographical correspondences (Calder, 1972), it combines several features rarely found together: extensive temporal range, dense psychological interiority, close observation of shame and dependency, and a sufficiently prolonged arc to capture both relational destruction and later existential reorganization. The methodological question is whether this complex narrative record contains structured semantic evidence of later-theorized mechanisms. This is consistent with evidence that readers’ behavioral and experiential responses to narrative do not differ sharply as a function of fictional versus factual framing (Hartung et al., 2017), suggesting that fiction is not categorically excluded from psychological relevance.

This logic may be described as pre-theoretical naturalistic convergence: the claim is not that Maugham consciously encoded modern constructs, but that later psychological constructs may capture patterns of social life that earlier narrative observers could represent in rich, naturalistic form (see Section 4.2 for further discussion).

### 1.2 From sentiment and traits to dynamic interpersonal mechanisms

The methodological problem can be stated more precisely: existing computational approaches to literature are either too general or too static for this question. One strand models narrative as affective movement—sentiment arcs or emotional valence over the plot (Jockers, 2015; Reagan et al., 2016; Kim and Klinger, 2019). A second pursues large-scale formal and thematic patterns across many texts (Moretti, 2013; Underwood, 2019), but rarely with specific psychological targets. Dictionary tools such as LIWC (Pennebaker et al., 2015) count words in pre-set categories rather than measuring contextual meaning. And character-profiling work, often using the Big Five (Flekova and Gurevych, 2015), captures stable traits rather than temporal entrenchment, reactive return, or patterned coercion.

What is needed instead is a design that can ask a different kind of question: not “Is the novel sad?” or “Is this character introverted?” but “Do semantically organized traces of coercion, traumatic attachment, helplessness, repeated re-investment, or existential repatterning cluster at specific scales of the narrative?” That question requires a framework that is both psychologically interpretable and sensitive to narrative grain.

### 1.3 CCR, TESA, and the present methodological position

Recent work in computational psychology offers a starting point. Contextualized Construct Representation (CCR) is a theory-driven method that compares text with the items of validated psychometric scales in embedding space, developed in response to the worry that much psychological text analysis is atheoretical and hard to interpret (Atari et al., 2023). A related approach, Text Embedding Similarity Analysis (TESA), likewise uses embeddings to ask hypothesis-driven questions of text rather than relying on inductive clustering or large labeled datasets (Vossen et al., 2025). Recent guidance stresses that embeddings can support psychological measurement only if researchers attend to their geometric properties, operational choices, and threats to validity (Teitelbaum and Simchon, 2025); practical tutorials have clarified model selection and transparency in such workflows (Hussain et al., 2024; Debelak et al., 2025).

The present study draws inspiration from that general logic but adapts it for literary narrative in a deliberately cautious way. It is CCR-inspired, but not a full psychometric implementation of CCR. The anchors used here are theory-informed, multi-variant semantic texts rather than direct item sets from validated scales. This matters because the present target domain—long-form literary narrative—differs substantially from survey response, self-report language, or short-form social text.

This position is also supported by emerging work showing that CCR-like methods can be extended to non-contemporary and historically distant texts. Chen et al. (2024), for example, adapt CCR for historical-psychological text analysis in classical Chinese, sharing the broader premise that psychologically interpretable structure may be recoverable from historically distant text when theory-driven semantic probes are used carefully.

### 1.4 What fiction can and cannot provide

It is worth stating directly what kind of evidence a novel can and cannot give. Empirical psychological data are records of real people: their responses, behaviors, or physiological signals. A novel is not such a record. *Of Human Bondage* does not give access to a real person’s inner life, and it cannot, in principle, diagnose anyone—least of all a character, who has no existence outside the text. The ontological status of the two kinds of material is therefore different in a way that no statistical procedure can erase. We do not treat the novel as clinical data, and none of the present claims concern the “true” mental state of a fictional person.

What the novel does contain is a structured verbal representation of social and emotional process, produced by a careful observer of human conduct. The unit of analysis here is precisely that representation—the language of the narrative—not a mind behind it. The question asked is therefore narrow and testable: does the text organize construct-related language (devaluation, coercion, intermittent reward, traumatic attachment, helplessness, warmth, existential re-patterning) in a non-random, locally concentrated, and domain-specific way?

This can be answered from the text itself, and the answer is reproducible. Three features keep the ontological gap from undermining the conclusions. First, the claim concerns semantic structure, not psychological fact, so it does not require the characters to be real. Second, discriminant safeguards—domain-specific negative controls (Section 3.4) and cross-model replication (Section 3.6)—show that the method responds to specific relational content rather than to “serious” or abstract prose in general. Third, an independent human-rater study (Sections 2.7 and 3.8) tests whether the model’s scores agree with independent human judgments of the same scenes, grounding the computational signal in human judgment.

This is also why the historical reading offered later (Section 4.2) is framed as convergence rather than anticipation or diagnosis: a novelist can record patterns of social life faithfully enough that later constructs recognize them, without the novel “containing” any theory. The relevant evidence is the patterned representation, and that is something a text can legitimately supply.

### 1.5 The present study

Against this background, the present study proposes a multiscale semantic analysis of “*Of Human Bondage*” using embedding-based comparison between narrative units and theory-driven anchor sets. The design combines chapter-level trajectory analysis across the full 122-chapter novel, scene-level localization within the focal late-novel corpus (Chapters 55–122), and negative-control anchors to test specificity. As a supplementary robustness check, three embedding models of different family and scale are compared.

This study makes three contributions. First, it introduces a CCR-inspired semantic anchor framework adapted for literary narrative. Second, it combines chapter-level inferential testing with scene-level localization, linking large-scale narrative trajectory to local narrative mechanism. Third, it incorporates negative controls to test whether the method merely rewards generic complexity or abstraction.

### 1.6 Research questions

*RQ1*. Do theory-driven construct profiles show non-random chapter-level organization across the novel, including both structural co-location and temporal continuity?

*RQ2*. Within the focal late-novel span, does the Mildred Arc show selective enrichment of a relational-process subcluster relative to the Resolution segment?

*RQ3*. Can chapter-level peaks be localized to distinct scene-level mechanisms, and are these local profiles distinguishable from domain-specific negative-control signals?

To evaluate robustness, the study additionally compares the main scene-level patterns across three embedding models of different family and scale, treating cross-model stability as a supplementary robustness analysis rather than as an independent research question (Table 1).

**Table 1:** Operational glossary of recurring terms, internal labels, and workflow steps.

Category	Term	Meaning in this study
Embedding basics	Embedding	A passage of text converted into a vector (list of numbers) arranged so that passages with similar meaning point in similar directions.
Embedding basics	Cosine similarity	How closely two embeddings point the same way; used here as “semantic proximity,” not as a psychometric score.
Text units	Narrative unit	A chapter or a scene—the text span whose embedding is compared with each anchor.
Text units	Chapter level/scene level	Two analysis scales: all 122 chapters (primary inferential tests) vs. 165 manually segmented scenes within Chs. 55–122 (used to localize chapter peaks).
Text units	Chunk	A ~ 350-word window that is embedded; chunk embeddings are pooled to form a chapter or scene embedding.
Text units	Trimmed-mean pooling	Averaging chunk embeddings after dropping the lowest and highest floor (0.1·n) values in each dimension; for short units this means no trimming (Section 2.5).
Constructs and anchors	Construct	A psychological pattern probed by an anchor: eight focal constructs plus two controls (Table 3).
Constructs and anchors	Semantic anchor	A short, theory-informed text expressing one construct (e.g., coercive control), compared against each narrative unit.
Constructs and anchors	Anchor variant (V0–V3)	Four paraphrases of each anchor, averaged so results do not depend on one wording (full texts in Supplementary Appendix S1).
Constructs and anchors	Construct-level anchor vector	The mean of a construct’s four unit-normalized variant embeddings; the cosine to this vector is the construct’s similarity value.
Constructs and anchors	Vector- vs. similarity-level aggregation	Averaging the variant vectors *then* taking cosine, vs. taking cosine to each variant *then* averaging; because the variants are unit-normalized these are mathematically equivalent here (Section 2.5).
Constructs and anchors	Positive-/negative-control anchor	Positive controls = the eight focal constructs; negative controls = two domain-specific controls (mathematical reasoning; geography/navigation) testing that the method is not merely rewarding “serious” or abstract prose.
Constructs and anchors	Construct profile	The sequence of one construct’s similarity scores across the 122 chapters or 165 scenes.
Constructs and anchors	Cronshaw signal	Shorthand for the Existential Patterning construct, so named because the Chapter 106 passage centers on Cronshaw’s “life as a pattern” idea.
Corpus structure	Focal corpus/focal arc	The late-novel span the study focuses on (Chapters 55–122).
Corpus structure	Mildred arc/resolution	The two chapter groups compared in Test 2—chapters labeled Mildred Arc vs. chapters labeled Resolution; full membership in Section 2.2.3 and Supplementary Appendix S2.
Corpus structure	Label layers	Three parallel chapter labelings (full-book, focal-arc, paper-ready); only the focal-arc layer drives the Test 2 contrast (Section 2.2.3).
Corpus structure	Internal key ↔ manuscript label	The code-side key (e.g., REF_NPD_BEHAVIOR) vs. the cautious reader-facing label (e.g., Narcissistic Relational Style); only the *display string* differs—anchor texts were frozen before analysis (Section 2.8; map in Table 3/Supplementary Appendix S1).
Analysis and validation	Co-location/localization	Co-location: several construct profiles peaking at the same chapters (Test 1). Localization: tracing a chapter-level peak to the specific scene(s) that generate it (Section 3.5).
Analysis and validation	Test 1/Test 2/Test 3	Cross-construct co-location across chapters; the Mildred Arc vs. Resolution difference; and chapter-to-chapter (lag-1) continuity.
Analysis and validation	Permutation test	A significance test comparing the observed statistic against many random reshufflings of the data.
Analysis and validation	Cross-model robustness	Whether the scene-level patterns recur across the three encoders (Section 3.6; Figure 9; full table in Supplementary Appendix S3).
Analysis and validation	Human-rater convergent validity	Agreement (Spearman) between mean human ratings and model scores across 20 blind scenes (Section 3.8; Table 8).

Terms are grouped by their role in the workflow: embedding basics, text units, constructs and anchors, corpus structure, and analysis/validation. Full construct definitions and the internal-key ↔ manuscript-label map are in Table 3 and Supplementary Appendix S1; the complete focal-arc chapter lists and the 165-scene inventory are in Supplementary Appendix S2.

## 2 Materials and methods

### 2.1 Study design

This study adopts a theory-driven, multiscale semantic mapping design. Narrative units from “*Of Human Bondage*” are compared with psychologically informed anchor texts using embedding-based similarity as a structured semantic proxy. The design operates on two linked scales—chapter and scene—whose distinct functions are summarized in Table 2. The measured object is therefore the semantic organization of narrative text; the later human-rating study is used as an external convergent-validity check rather than as a reader-response experiment.

**Table 2:** Corpus structure and analytical scales used in the study.

Scale	Coverage	Unit count	Primary use	Label layer
Chapter level	Full novel (Chs. 1–122)	122 chapters	Primary inferential layer: Test 1, Test 2, Test 3	Full-book labels; focal-arc labels where available
Scene level	Focal corpus (Chs. 55–122)	165 scenes	Localization of chapter peaks and local mechanism decomposition	Arc / cluster / focal labels from final scene segmentation

The table summarizes the two analytic scales used in the study and their distinct functions in the multiscale design.

### 2.2 Corpus construction

#### 2.2.1 Chapter-level corpus

The chapter-level corpus consists of the full 122 chapters of “*Of Human Bondage*”. Each chapter was represented as a chapter embedding derived from chunked full-text chapter content. The chapter file also preserves the label fields used for whole-novel orientation and for the focal-arc contrast; these label layers are defined in Section 2.2.3.

This full 122-chapter corpus design serves two purposes. It allows the focal late-novel structures to be interpreted against the background of the entire novel, and it preserves the continuous chapter sequence needed for chapter-scale temporal organization analyses.

#### 2.2.2 Scene-level corpus

The scene-level corpus consists of a manually segmented focal dataset covering Chapters 55–122. Each scene is stored as an individual JSON object with chapter number, scene index, arc and cluster labels, focal labels, scene text, word count, and segmentation rationale. Scene segmentation was based on narrative-functional shifts rather than sentence count or arbitrary token windows. Each scene was defined as a coherent local narrative unit, such as a humiliation sequence, a reflective passage, a leverage episode, a philosophical breakthrough, or a settlement decision.

The goal of the scene corpus is not to replace chapter-level inference, but to identify which local scenes actually drive the larger peaks observed at chapter scale.

#### 2.2.3 Focal-arc labels and the two focal groups

Each chapter carries three parallel label layers, kept separate so that book-scale context and focal-arc analysis do not contaminate each other. (i) A full-book layer gives a whole-novel phase/group label for all 122 chapters and is used only for orientation and figure shading. (ii) A focal-arc layer, defined for the focal span, carries the macro-group field used in the Test 2 comparison; its macro-groups are Mildred Arc, Resolution, Norah Contrast, Contextual Prelude, Cronshaw Anchor, and Transition. (iii) A paper-ready display layer supplies standardized reader-facing names (provisional for Chs. 1–54, scene-informed for Chs. 55–122; original values retained as legacy fields). Only the focal-arc layer drives the inferential contrast; the other two are descriptive.

The two focal groups are defined operationally as follows. The Mildred Arc (40 chapters: 55–65, 69–80, 90–105, 109) comprises the focal chapters whose narrative function centers on Philip’s relationship with Mildred—its initiation, the repeated ruptures and re-engagements, the cohabitation crisis, and the poverty and collapse that follow from that entanglement. The Resolution segment (13 chapters: 110–122) comprises the closing focal chapters organized around Philip’s movement toward settlement—the Athelny/Sally line and his vocational and marital resolution, including the relinquishing of the earlier bond. The remaining 15 focal chapters (Norah Contrast, 66–68; Contextual Prelude, 81–89; Cronshaw Anchor, 106; Transition, 107–108) belong to neither group and are excluded from the Test 2 contrast by design. Chapter-level focal-arc labels for Chs. 55–122 were derived from the scene segmentation described above so that the two scales are mutually consistent. Labeling was performed by the author; because such labeling involves interpretive judgment, the complete per-chapter and per-scene assignments are published in Supplementary Appendix S2 so that any reader can audit them.

### 2.3 Construct anchors

#### 2.3.1 Anchor design principle

Eight positive-control anchor sets were used. Each anchor was represented not by a single paragraph, but by four theory-informed variants (V0–V3). These variants were designed to reduce dependence on a single formulation and to stabilize the construct representation across stylistic differences. In all cases, the anchor texts were written to describe interactional dynamics, internal states, and relational patterns rather than to rest on overt diagnostic vocabulary. The anchors were finalized prior to the final multiscale analyses and were not rewritten in response to model outputs. This preserves the logic of theory-first rather than result-driven text probing.

#### 2.3.2 Positive-control constructs

The positive-control anchors were selected to capture theory-linked mechanism families relevant to relational narcissism, coercive asymmetry, intermittent reward, traumatic attachment, reduced agency, affiliative security, and meaning-oriented reorganization. For readability the manuscript uses brief labels, while the original internal keys are retained in the code and JSON outputs for reproducibility (the internal-key ↔ label map is given in Table 3 and Section 2.8).

**Table 3:** Positive- and negative-control semantic anchor families.

Internal key	Manuscript label	Type	Functional description
REF_NPD_BEHAVIOR	Narcissistic relational style (NRS)	Positive	Devaluation, exploitative asymmetry, humiliation, and low-empathy relational framing
REF_COERCIVE_CONTROL	Coercive control	Positive	Control through pressure, leverage, intimidation, or constrained options
REF_INTERMITTENT_REINFORCEMENT	Intermittent reinforcement	Positive	Alternation of reward and frustration that deepens attachment
REF_GAMBLERS_FALLACY	Repeated-investment logic	Positive	Escalating investment despite worsening returns or poor prospects
REF_TRAUMA_BONDING	Trauma bonding	Positive	Attachment under pain, degradation, fear, and relational instability
REF_LEARNED_HELPLESSNESS	Learned helplessness	Positive	Reduced agency under repeated failure, exhaustion, or futility
REF_HEALTHY_LOVE	Relational warmth	Positive	Warmth, care, mutuality, and affiliative security
REF_CRONSHAWS_PHILOSOPHY	Existential patterning	Positive	Pattern, meaning, existential reorganization, and life-design language
REF_MATHEMATICAL_REASONING	Math reasoning [NEG]	Negative	Analytical or abstract reasoning not specific to the focal relational arc
REF_GEOGRAPHY_NAVIGATION	Geography/navigation [NEG]	Negative	Travel, route-planning, spatial orientation, and movement scripting

The table lists the internal anchor keys, manuscript-facing labels, control type, and brief functional descriptions used throughout the study.

Table 3 gives a brief functional description and the internal key for each construct, and the full anchor texts (V0–V3) appear in Supplementary Appendix S1; here we note only the interpretive qualifications that the brief labels leave implicit. Narcissistic Relational Style (NRS) is a semantic anchor for a relational *style*—devaluation, exploitative asymmetry, humiliation, and low empathy—not a clinical diagnosis (Miller et al., 2007; Schalkwijk et al., 2021). Repeated-Investment Logic is an auxiliary mechanism marker rather than a formal psychometric construct, conceptually closer to escalation-of-commitment and sunk-cost reasoning than to a literal gambling error (Tversky and Kahneman, 1974; Staw, 1976; Arkes and Blumer, 1985). Relational Warmth is a proxy for warmth, mutuality, and affiliative security rather than a definitive detector of “healthy love” in any clinical or normative sense (cf. Bowlby, 1988). Existential Patterning is not a psychopathology construct but a text-internal convergent-validity anchor, concentrated around the Chapter 106 “life as a pattern” sequence (cf. Frankl, 1959). The remaining four follow standard definitions in the cited literature: Coercive Control (Stark, 2007; Johnson, 2008), Intermittent Reinforcement (cf. Ferster and Skinner, 1957), Trauma Bonding (Dutton and Painter, 1993; Herman, 1992), and Learned Helplessness (Seligman, 1972; Abramson et al., 1978).

#### 2.3.3 Negative-control anchors

Two negative-control anchor sets were added (see Table 3): mathematical reasoning and geography/navigation. These are not expected to remain uniformly flat across the novel but instead function as domain-specific negative controls whose purpose is to test whether the main results merely reflect generic abstraction or discursive complexity. A successful negative control responds selectively to its own semantic domain without reproducing the focal toxic-relational arc.

### 2.4 Embedding models

The analysis used one primary encoder and two secondary encoders for robustness checks (see Table 4).

**Table 4:** Embedding models used in the primary analysis and robustness checks.

Model	Parameters	Role
Qwen/Qwen3-Embedding-8B	8B	Primary model
sentence-transformers/all-MiniLM-L6-v2	22 M	Cross-family baseline
Qwen/Qwen3-Embedding-0.6B	0.6B	In-family contrast model

The comparison varies two factors—model family and parameter scale—while holding the task (passage embedding) fixed. Qwen3-Embedding-8B was selected as the primary encoder because, at the time of analysis, it was a first-tier open-weight embedding model—the top-ranked system on the MTEB multilingual leaderboard and the largest (8B parameters) in its family (Qwen Team, 2025). Using the strongest available general-purpose encoder makes the recovery of construct structure a conservative test rather than an artifact of a weak encoder. It also sets a deliberately demanding bar: as Section 3.6 reports, the smaller in-family model in fact draws *sharper* anchor–control margins on these scenes, so designating the benchmark-leading 8B as primary—and the smaller and cross-family models as robustness comparisons—guards against the concern that the findings depend on a model tuned to maximize local separation. MiniLM (all-MiniLM-L6-v2, 22M parameters) provides a lightweight, widely used cross-family baseline built on the Sentence-BERT architecture (Reimers and Gurevych, 2019; cf. Devlin et al., 2019), and Qwen3-Embedding-0.6B provides a same-family scale contrast that isolates parameter count from architecture. Both chapter-level and scene-level results are based primarily on Qwen3-Embedding-8B; the two secondary models were used not to replace the primary analysis but to assess whether the large-scale and local patterns observed under Qwen3-8B were broadly recoverable across family and scale (Section 3.6). Encoding parameters and aggregation rules for all three models are reported in Section 2.5.

### 2.5 Text chunking, aggregation, and similarity computation

#### 2.5.1 Chapter representation

For chapter-level analysis, full chapter texts were segmented into overlapping word-based chunks (chunk_words = 350, stride_words = 175) and then encoded. Word-based chunking is independent of the model, so chunk counts are identical across the three encoders; chapters yielded 3–21 chunks (median 11). Chapter embeddings were built by aggregating the normalized chunk embeddings using a trimmed mean with trim_frac = 0.1. Anchor variants were aggregated separately into construct-level anchor vectors using the (vector) mean. The trimmed mean was applied per dimension across the chunk set: for *n* chunks, the floor (0.1·n) lowest and highest values were removed in each dimension before averaging. Thus, for *n* < 10, no values are removed and the operation reduces exactly to the ordinary mean. The aggregated vector was then re-normalized to unit length before similarity computation. Given the chunk counts above, trimming therefore affects only the 88 chapters with ≥ 10 chunks (at most two chunks per tail) and reduces to plain mean pooling for the remaining 34 chapters; robustness to this choice, and to plain-mean and pairwise alternatives, is reported in Section 3.7. All models were encoded on CPU in float32 with normalize_embeddings = true. For Qwen3-8B and Qwen3-0.6B, max_seq_length = 1,024; for MiniLM, max_seq_length = 512. Batch sizes were 4 (Qwen3-8B) and 16 (MiniLM, Qwen3-0.6B).

#### 2.5.2 Scene representation

For scene-level analysis, each scene text was first segmented into overlapping chunks (chunk_words = 350, overlap_words = 75). Each chunk was encoded, and the resulting normalized chunk embeddings were aggregated into a scene embedding using the same trimmed mean (trim_frac = 0.1, per dimension, re-normalized), with anchor variants again aggregated by mean. Scenes contained 2–6 chunks (median 3); because floor(0.1·n) = 0 for every *n* < 10, the trimmed mean reduces exactly to the ordinary mean for all 165 scenes, so the trimming choice is immaterial at the scene scale. This procedure ensured that long scenes were not silently truncated and that chapter- and scene-level embeddings were methodologically aligned.

#### 2.5.3 Similarity computation

For each narrative unit (chapter or scene) and each construct anchor, cosine similarity was computed between the embedding of the narrative unit and the embedding of the construct anchor:

$$\mathrm{sim}(u,c)=\frac{u\cdot c}{∥u∥∥c∥}$$

Where *u* is the embedding of a chapter or scene and *c* is the construct-level embedding derived from the anchor variants. Here *c* is the mean of the four unit-normalized variant embeddings (a “vector-level” aggregation). An alternative “similarity-level” aggregation computes cosine similarity to each variant and then averages the four similarities. Because the variants are unit-normalized, this value is equal to the vector-level score multiplied by a positive constant that depends only on the construct, not on the narrative unit. The two therefore yield identical rank orderings, identical standardized effect sizes, and identical permutation *p*-values; this is confirmed empirically in Section 3.7.

The resulting similarity scores are interpreted as semantic proximity measures, not as psychometric scores.

### 2.6 Statistical strategy

#### 2.6.1 Chapter-level inferential tests

The chapter level serves as the primary inferential layer. Three tests were used.

##### 2.6.1.1 Test 1: cross-construct consistency (full 122-chapter sequence)

Using the full 122-chapter sequence, positive-control construct profiles were tested for non-random co-location. The observed rank-consistency statistic was compared against a permutation null in which each construct profile was independently shuffled across chapters. The purpose of this test is to determine whether the construct profiles tend to rise and fall together at the same chapter positions more often than expected by chance.

##### 2.6.1.2 Test 2: focal group differentiation (chapters 55–122)

Within the focal late-novel range, only chapters with non-null labels.focal_arc were retained. The primary contrast compared chapters labeled as Mildred Arc with those labeled as Resolution under labels.focal_arc.macro_group; other focal chapters were excluded from this specific group-difference test. Permutation-based mean-difference tests were conducted for each construct, with Hedges’ *g* reported as effect size (Hedges, 1981) and Benjamini–Hochberg FDR correction applied across constructs (Benjamini and Hochberg, 1995).

##### 2.6.1.3 Test 3: temporal organization (full 122-chapter sequence primary; chapters 55–122 supplementary)

Lag-1 autocorrelation was computed for each construct profile. The primary version of this test used the complete 122-chapter sequence, which provides a genuinely continuous narrative series. A supplementary version repeated the analysis on the focal 55–122 subset. Significance was assessed via permutation.

#### 2.6.2 Scene-level explanatory analyses

The scene level is treated as an explanatory and localization layer rather than as a second fully independent inferential framework. After major chapter-scale peaks were identified, scene-level results were used to determine which local narrative units actually generated those peaks. In practice, this meant examining the top-scoring scenes for each construct, the internal decomposition of key high-scoring chapters (especially the cohabitation crisis, the poverty-collapse sequence, and the Persian-rug epiphany), and the contrast between positive-control and negative-control peaks within those local sequences. The scene level therefore answers a different question from the chapter level: not whether a construct is globally structured across the whole novel, but where, locally, a chapter-scale signal is concentrated.

#### 2.6.3 Robustness analyses

Two families of robustness check were run. First, to test sensitivity to the aggregation choices, the cached Qwen3-8B chunk and variant embeddings were evaluated under three alternative schemes—similarity-level anchor aggregation, plain-mean chunk pooling, and pairwise chunk–anchor similarity—and Tests 2 and 3 were reported or recomputed as appropriate under each scheme (Section 3.7). Second, to test sensitivity to the encoder, the scene corpus was re-scored under MiniLM and Qwen3-0.6B and compared with the primary model using rank-based statistics (Section 3.6).

### 2.7 Human-rater validation

To provide external, non-computational evidence, a blind convergent-validity study was designed. Twenty passages were drawn from the focal scene corpus to span the model’s score range on every rating dimension: the ten scenes that the analysis identifies as the major chapter peaks (Section 3.5), discriminator scenes high on a single construct, and low/flat scenes. These 20 passages therefore constitute a targeted validation sample—selected to cover the score range and the scenes on which the manuscript’s claims rest—rather than a random or representative sample of the 165-scene corpus. Passages were stripped of all labels and identifiers, assigned neutral codes in randomized order, and split across two sessions to limit fatigue. Six adult volunteer raters (coded R1–R6), all proficient readers of English-language literary fiction, independently rated each passage for how strongly each of ten rating dimensions was present on a 0–4 scale, blind to the model scores. Raters’ formal training included literature, linguistics, and translation, with one rater also trained in psychology. A sensitivity subset restricted to the three raters with formal literary or translation training (R1, R2, R5) is reported separately, and leave-one-out analysis confirms that no single rater drives the result (Section 3.8). Raters scored the English passages directly—the same text the embeddings scored—using only the neutral, non-clinical construct definitions supplied in the packet. No clinical or diagnostic expertise was assumed or required. The two negative-control domains were presented in the same format as the eight focal constructs and were not flagged as controls, so that the discriminant check—whether raters independently kept those domains low—rested on their own judgments. Inter-rater reliability is summarized with ICC(2,k) (two-way random, average-measures, absolute-agreement) and Krippendorff’s *α* (ordinal); convergent validity is the Spearman correlation between the mean human rating and the model cosine across passages, computed per rating dimension and reported with bootstrap 95% confidence intervals (10,000 passage resamples) and two-sided permutation *p*-values (10,000 permutations). The full rating protocol, construct definitions, anonymized rating data (R1–R6), and analysis script are provided in the Supplementary material.

### 2.8 Reproducibility and internal labels

**Figure 1:**
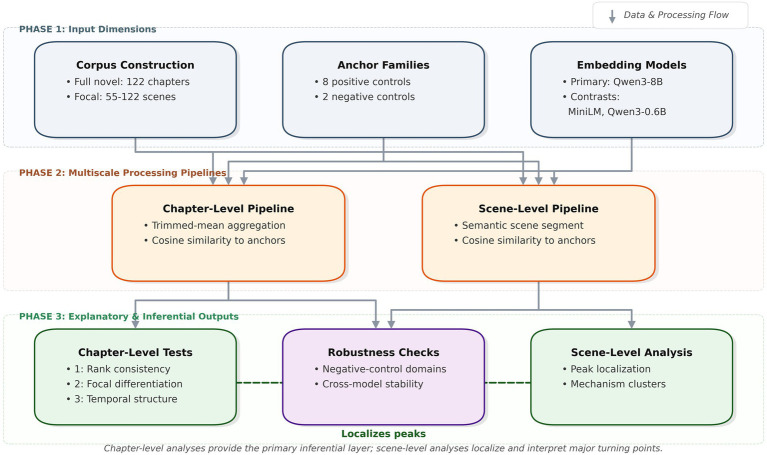
Multiscale theory-driven embedding workflow. Overview of the analytical pipeline. Full-text chapters were chunked and aggregated to produce chapter embeddings for the complete 122-chapter corpus. A manually segmented focal scene corpus spanning Chapters 55–122 was processed with the same chunking and aggregation logic. Similarity was computed between narrative units and multi-variant positive- and negative-control anchors. Chapter-level analyses supported inferential tests of cross-construct consistency, focal-group differentiation, and temporal organization, while scene-level analyses localized the chapter peaks and clarified their internal narrative composition.

For reproducibility, all internal result files preserve the original anchor keys, including REF_NPD_BEHAVIOR and REF_HEALTHY_LOVE. In the manuscript these are reported using the more cautious labels Narcissistic Relational Style (NRS) and Relational Warmth, to avoid diagnostic overclaiming and to better match the semantic breadth actually observed in the outputs. Crucially, this relabeling changed only the display string: the anchor *texts* were finalized before analysis and never altered. This provenance is verifiable: the cached embeddings that generate the published scores carry a run signature recording the SHA-256 of the anchor file used at encoding time; the released anchor files reproduce those hashes exactly (anchor_variants.json, negative_controls.json) and the cached embeddings reproduce the released chapter similarities to within floating-point error. The full text of every anchor variant (V0–V3) and the internal-key ↔ manuscript-label map are given in Supplementary Appendix S1. The distinction between internal reproducibility labels and external interpretive labels is maintained throughout the manuscript (see Figure 1).

## 3 Results

### 3.1 Chapter-level cross-construct consistency (test 1)

**Figure 2:**
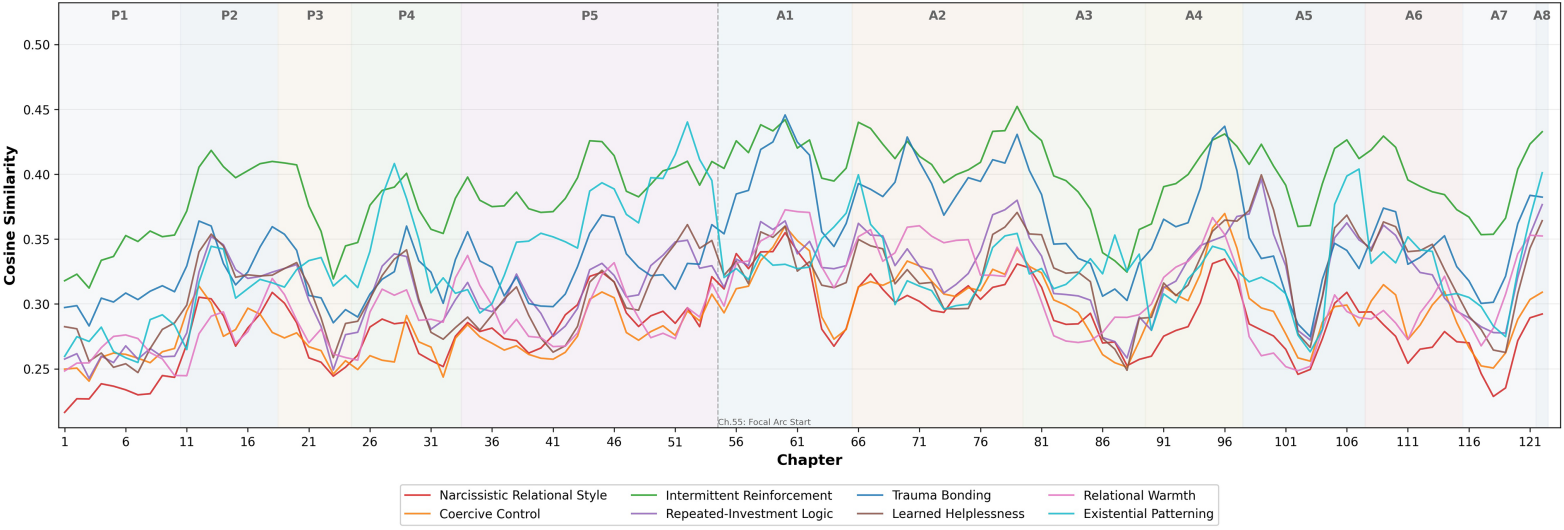
Chapter-level construct trajectories across the full 122-chapter novel. Chapter-level cosine-similarity profiles for the eight positive-control constructs under the primary Qwen3-Embedding-8B model. The figure is descriptive rather than inferential: it visualizes the broad chapter-scale trajectories that are formally tested in Sections 3.1–3.3. Background shading marks the major full-book phases and focal-arc segments used for narrative orientation.

The first chapter-level question was whether the positive-control construct profiles behaved as isolated signals, or whether they tended to co-locate at the same chapter positions more often than expected by chance. To test this, a cross-construct rank-consistency permutation test was run on the full 122-chapter corpus (see Figure 2).

The result was strongly significant. The observed rank-consistency statistic was 847.12, whereas the permutation null had a mean of 154.76 (SD = 18.61), yielding *p* < 0.001. This indicates that the chapter-level construct profiles are not distributed independently across the novel. Rather, some chapters repeatedly emerge as multi-construct peaks, whereas others remain consistently low across several constructs. In other words, the construct signals cluster structurally across the chapter sequence instead of appearing as isolated, unrelated spikes.

**Figure 3:**
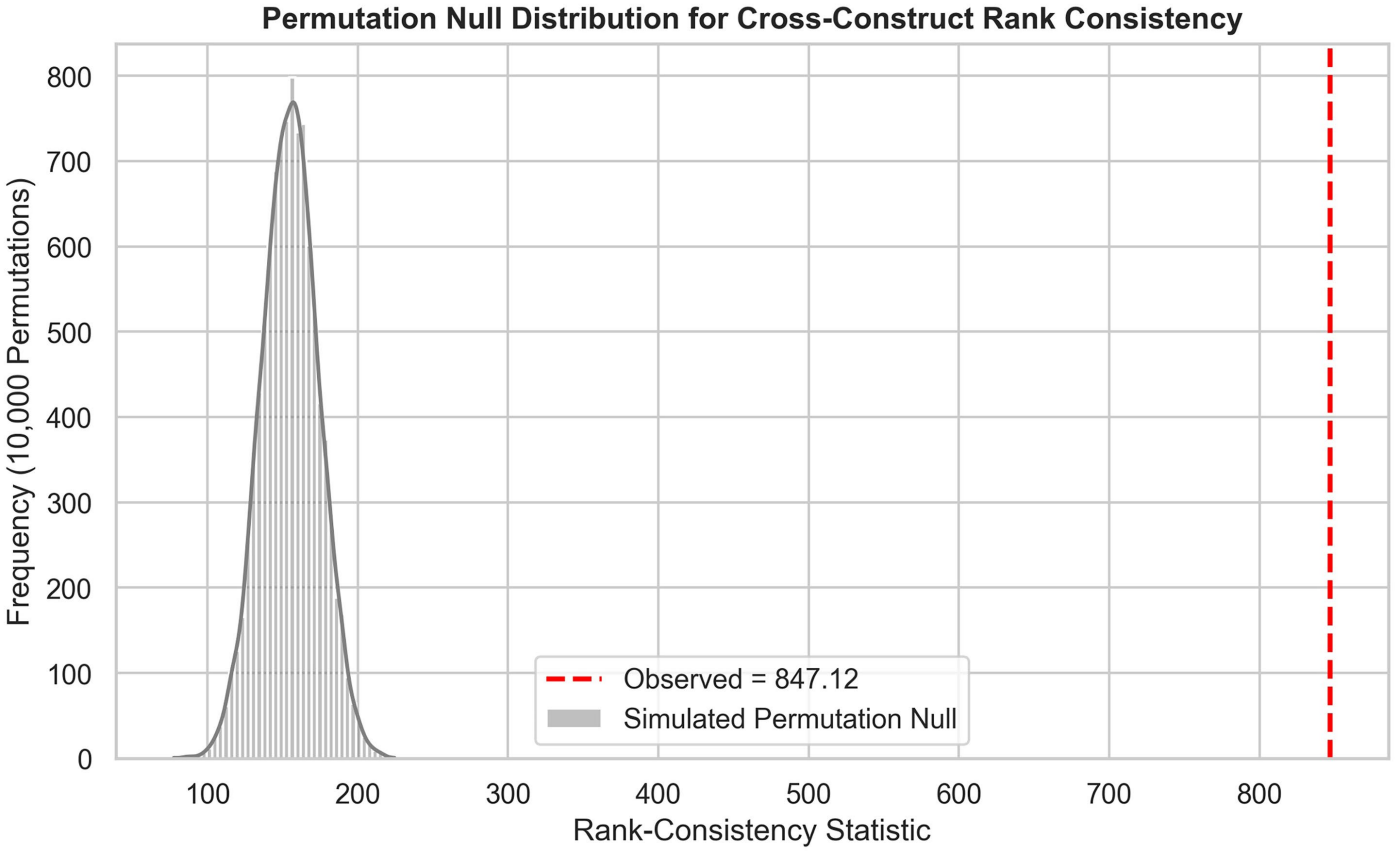
Permutation null distribution for cross-construct rank consistency (test 1). Null distribution of the cross-construct rank-consistency statistic obtained by independently permuting the full-122 chapter profiles of the positive-control constructs. The vertical reference line marks the observed statistic. The strong separation between the observed value and the permutation null supports non-random co-location of construct profiles across the chapter sequence.

Some degree of co-location is inherently expected from semantic overlap among the anchors themselves, but the magnitude of the observed statistic relative to the permutation null suggests that the chapter-level co-organization in the novel exceeds what that baseline overlap alone would predict. The chapter peaks are therefore interpretable as structured narrative zones rather than as arbitrary local similarities between individual anchors and isolated passages (see Figure 3).

### 3.2 Chapter-level group differentiation within the focal arc (test 2)

The second test examined whether the focal late-novel span (Chapters 55–122) showed systematic differences between the chapters labeled as Mildred Arc and those labeled as Resolution. Only chapters with non-null labels.focal_arc were retained for the focal subset. Within that set, the primary contrast compared 40 Mildred Arc chapters with 13 Resolution chapters. Other focal chapters belonging to neither of these two macro-groups were excluded from this specific group-difference test by design.

Permutation-based mean-difference tests revealed a clear pattern (see Table 5 for full results). Four constructs remained significant after Benjamini–Hochberg FDR correction: NRS (*g* = 1.13), Coercive Control (*g* = 0.94), Trauma Bonding (*g* = 0.87), and Intermittent Reinforcement (*g* = 0.81). All four were higher in the Mildred Arc, with effect sizes ranging from large to very large. This indicates that the Mildred sequence is not simply “emotionally intense” in a general sense but is enriched for a specific relational mechanism cluster involving devaluation, coercion, repeated re-engagement, and traumatic attachment. Just as importantly, only a specific subset of positive anchors reached significance, arguing against a nonspecific positivity bias.

**Table 5:** Chapter-level group differences (Mildred arc vs. resolution).

Construct	Mildred Arc *M*	Resolution *M*	Δ	*p*	*q* (BH)	Hedges’ *g*
Narcissistic relational style (NRS)	0.3031	0.2639	+0.0393	0.0011	0.0088	1.13
Coercive control	0.3161	0.2825	+0.0337	0.0058	0.0211	0.94
Intermittent reinforcement	0.4137	0.3877	+0.0260	0.0134	0.0268	0.81
Trauma bonding	0.3824	0.3392	+0.0433	0.0079	0.0211	0.87
Repeated-investment logic	0.3398	0.3158	+0.0240	0.0574	0.0918	0.60
Learned helplessness	0.3335	0.3172	+0.0163	0.2490	0.2845	0.37
Relational warmth	0.3248	0.3036	+0.0213	0.1123	0.1497	0.51
Existential patterning	0.3215	0.3264	−0.0049	0.6607	0.6607	−0.14

Permutation-based mean-difference tests comparing chapters labeled as Mildred Arc and Resolution within the focal late-novel subset. Positive Δ values and positive Hedges’ g values indicate higher mean similarity in the Mildred Arc. Benjamini–Hochberg corrected *q* values are reported for multiple-comparison control.

**Figure 4:**
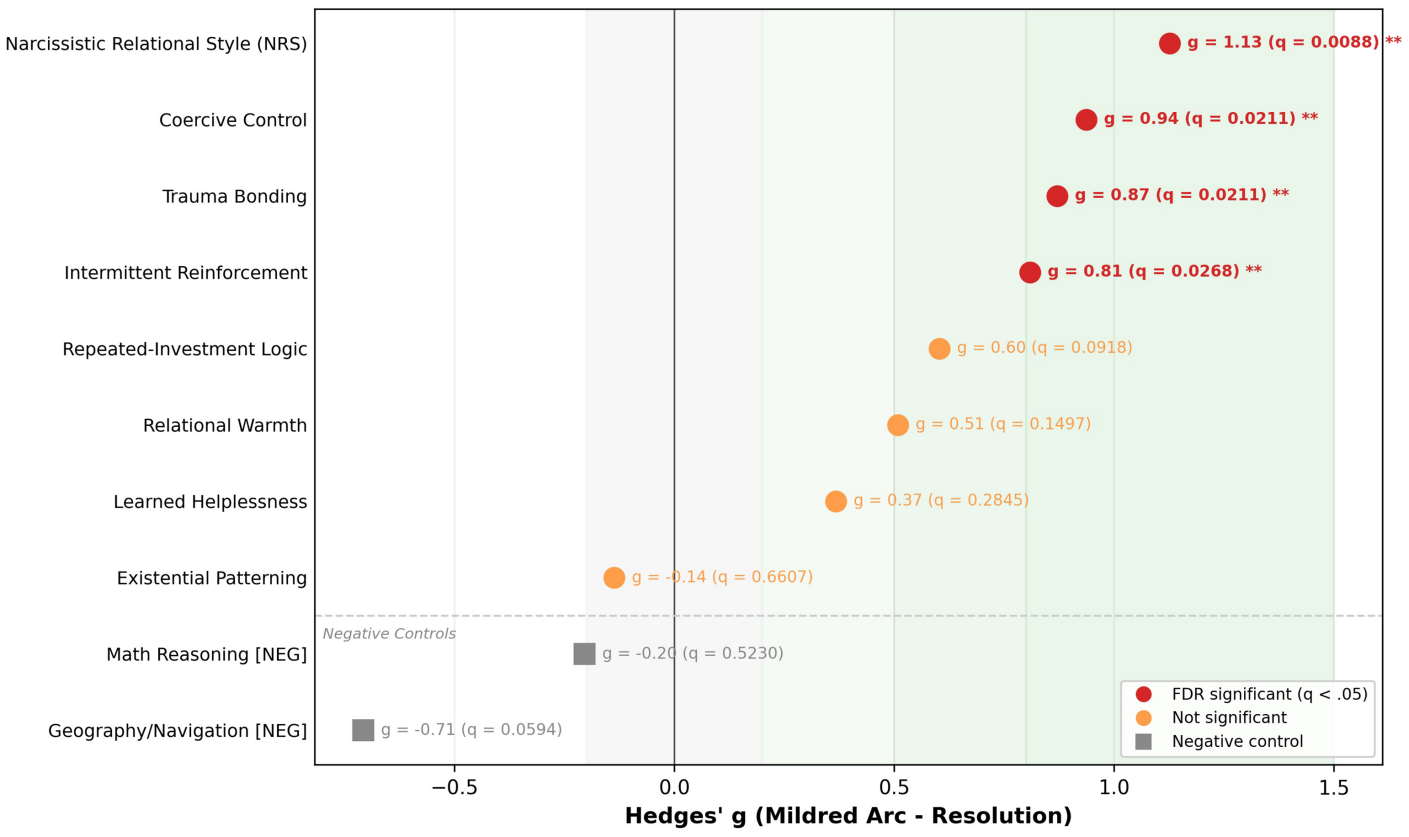
Effect sizes for focal group differences (Mildred arc vs. resolution). Forest plot of chapter-level effect sizes for the focal late-novel comparison. Positive Hedges’ g values indicate higher similarity in the Mildred Arc than in the Resolution chapters. The figure complements Table 5 by visualizing the magnitude, direction, and selectivity of the focal contrast across constructs.

Three other positive-control constructs—Repeated-Investment Logic, Relational Warmth, and Learned Helplessness—did not survive FDR correction but remained directionally positive. Their failure to reach significance is consistent with the narrative structure: learned helplessness, for instance, appears not only within the Mildred Arc but also in Philip’s broader vocational and existential struggles, while relational warmth is not confined to Resolution chapters. Existential Patterning showed no meaningful group differentiation (*g* = −0.14, *q* = 0.661), which is theoretically appropriate: this construct—hereafter occasionally referred to as the Cronshaw signal—is not expected to behave as a focal abuse-related construct but instead concentrates around the later existential reorientation sequence (see Figure 4).

### 3.3 Chapter-level temporal organization (test 3)

The third chapter-level question concerned temporal organization: do adjacent chapters resemble one another in construct similarity more than expected under permutation? This was assessed through lag-1 autocorrelation tests.

#### 3.3.1 Full novel (122 chapters)

Across the full 122-chapter sequence, all eight positive-control constructs showed positive lag-1 autocorrelation, and all were significant after FDR correction (see Table 6). The strongest temporal continuity was observed for Relational Warmth (*r*_1_ = 0.49) and Trauma Bonding (*r*_1_ = 0.48), with the weakest but still significant result for Existential Patterning (*r*_1_ = 0.19, *q* = 0.014). Adjacent chapters therefore tend to remain locally similar in construct profile, consistent with the novel organizing its relational and existential mechanisms in extended arcs rather than isolated bursts.

**Table 6:** Lag-1 autocorrelation for chapter-level construct profiles.

Construct	Full122 *r*₁	Full122 *q*	Focal55–122 *r*₁	Focal55–122 *q*
Narcissistic relational style (NRS)	0.4414	<0.001	0.4754	<0.001
Coercive control	0.3908	<0.001	0.4394	<0.001
Intermittent reinforcement	0.4553	<0.001	0.4364	<0.001
Repeated-investment logic	0.3952	<0.001	0.3320	0.0021
Trauma bonding	0.4842	<0.001	0.5116	<0.001
Learned helplessness	0.3586	<0.001	0.2946	0.0042
Relational warmth	0.4863	<0.001	0.5247	<0.001
Existential patterning	0.1906	0.0142	−0.0045	0.4554

Lag-1 autocorrelation coefficients for the chapter-level construct profiles, calculated for both the full 122-chapter sequence and the focal 55–122 subset. Permutation-based q values indicate which trajectories are more temporally organized than expected by chance.

#### 3.3.2 Focal subset (55–122)

The same analysis repeated on the 68 focal chapters (55–122) yielded a similar pattern: seven of eight constructs remained significantly autocorrelated, with Relational Warmth (*r*_1_ = 0.52) and Trauma Bonding (*r*_1_ = 0.51) again strongest. The only exception was Existential Patterning (*r*_1_ = −0.005, *q* = 0.455), which is expected: the Cronshaw signal is concentrated narrowly around the late existential-reorientation zone rather than forming a broad smooth trajectory across the entire focal arc.

### 3.4 Negative-control robustness at the chapter level

A crucial question is whether the observed structure simply reflects generic abstraction, discursive complexity, or any kind of “serious prose.” To test this, two domain-specific negative controls were introduced: mathematical reasoning and geography/navigation.

#### 3.4.1 Group contrast

In the focal Mildred Arc vs. Resolution comparison, neither negative control showed the same pattern as the positive relational cluster. Mathematical Reasoning showed negligible differentiation (*g* = −0.20, *q* = 0.523), whereas Geography/Navigation trended in the opposite direction from the positive cluster (*g* = −0.71, *q* = 0.059), with higher values in Resolution than in the Mildred Arc. This contrast is theoretically plausible, since the Resolution segment contains more travel- and route-oriented future scripting, whereas the Mildred Arc chapters are more tightly organized around interpersonal entrapment.

#### 3.4.2 Temporal organization

At the full 122-chapter scale, mathematical reasoning showed modest but significant lag-1 structure (*r*_1_ = 0.25, *q* = 0.005), whereas geography/navigation did not (*r*_1_ = 0.03, *q* = 0.327). In the focal 55–122 subset, neither negative control showed significant lag-1 organization (Mathematical Reasoning: *r*_1_ = 0.01, *q* = 0.808; Geography/Navigation: *r*_1_ = −0.12, *q* = 0.823). The negative controls are therefore not completely flat—some chapters genuinely contain more reasoning-heavy or travel-oriented content—but crucially, they do not reproduce the focal late-novel toxic-relational arc.

### 3.5 Scene-level localization of chapter peaks

Sections 3.1–3.4 established that the construct signals form a non-random, time-organized system that separates the Mildred Arc from the Resolution chapters. The scene-level analysis now asks a different question: within a high-scoring chapter, where is the signal actually concentrated? It is a localization layer, not a second round of inference.

Across the focal corpus, the strongest chapter peaks do not come from uniformly high scores throughout a chapter; they come from a few local scenes where several construct signals converge. This convergence is clearest in four zones: the initial Mildred sequence (A1), the cohabitation crisis (A4), the poverty-collapse and existential-reorientation sequence (A5), and the late Sally/settlement sequence (A7–A8).

#### 3.5.1 A1: initial hooking, rumination, and return

The earliest focal scenes already show that the Mildred sequence is not driven by a single instantaneous attraction but by a chain of humiliation, rumination, and compelled return. In the chapter-level results, the early Mildred Arc chapters emerged as part of the late-novel toxic-relational trajectory. Scene decomposition clarifies how that early loading is generated.

The clearest early scene is ch055_s03 (“Post-Snub Rumination”). Philip’s wounded pride, compulsive return, and revenge fantasy produce one of the earliest strong multi-anchor patterns in the corpus: intermittent reinforcement, trauma bonding, repeated-investment logic, and NRS all rise together, while the controls stay lower. The point is not that these constructs are fully formed this early, but that the scene sets up the reactive loop the later arc elaborates—humiliation leads not to withdrawal but to renewed investment. The text makes this plain: after the slight, Philip admits the insult had “rankled” and that it would be “least trouble to see her” again (Maugham, 1915, ch. 55).

**Figure 5:**
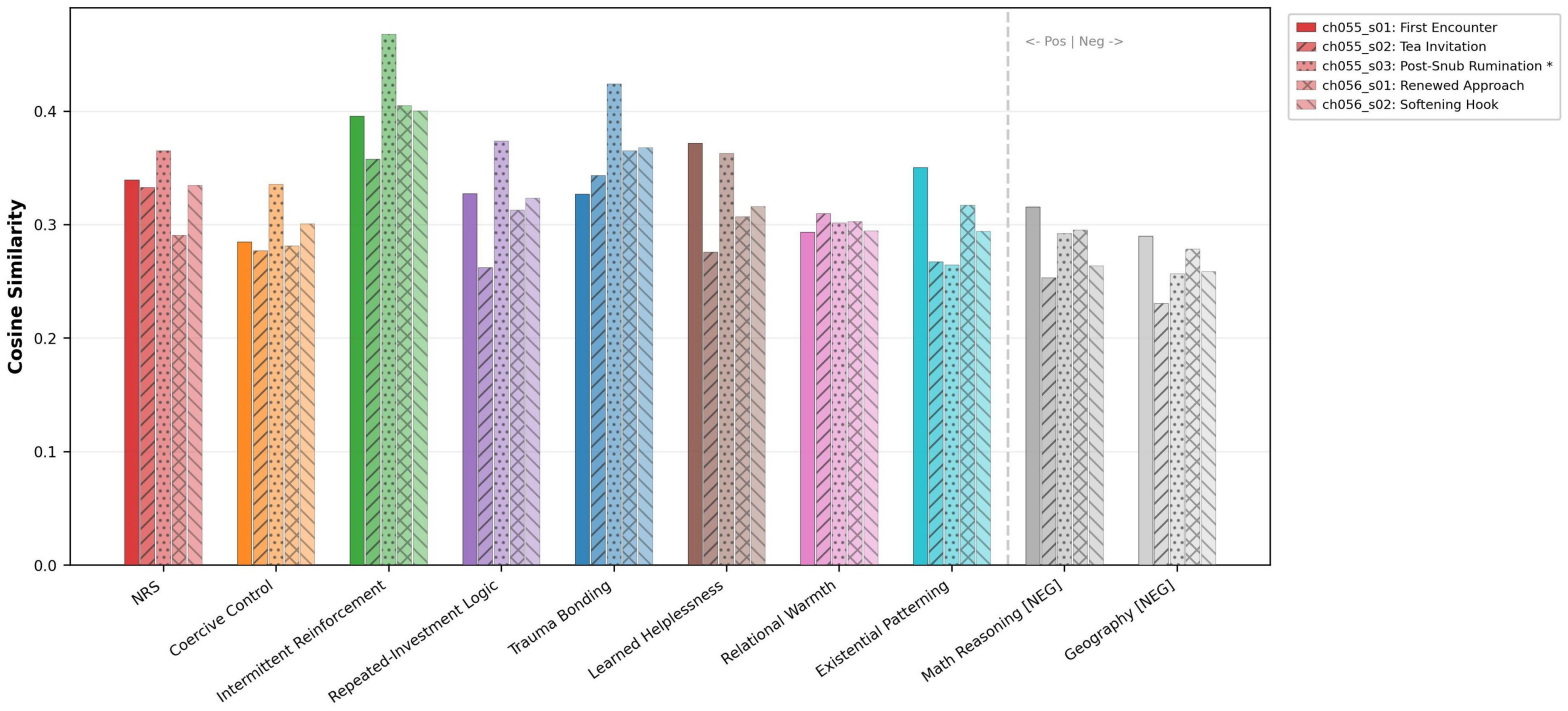
Scene-level localization of the early Mildred hooking sequence. Local scene profiles from the early Mildred sequence showing how humiliation, rumination, and compelled return are concentrated in a small number of narrative units. The highlighted scenes center on the early hooking zone around ch055_s03 and adjacent scenes, illustrating that the chapter-scale signal is locally concentrated rather than evenly distributed across the chapter pair.

This logic continues in the subsequent early scenes (ch056_s01, ch056_s02, ch057_s02, ch058_s01), where the pattern is repeatedly the same: rebuff, softening, jealousy, brief repair, renewed approach. The scene-level pattern therefore helps explain why the early Mildred Arc chapters are already chapter-level active: the construct load is not diffuse, but repeatedly concentrated in compact local scenes of injury → return → partial reward → deeper investment (see Figure 5).

#### 3.5.2 A4: Mildred cohabitation and domestic breakdown

The strongest example of scene-level localization appears in the Chapter 96 cohabitation crisis, where the chapter-level peak is clearly decomposed into multiple distinct local mechanisms rather than one undifferentiated high chapter.

The first key scene is ch096_s02 (“Brighton Failure and Discovery of Lost Control”). It captures Philip’s discovery that Mildred no longer commands his old responses, even as she tries to regain leverage through the child and through her position in the household. This is the main local site where trauma-bonding, coercive, and devaluing/exploitative signals co-occur. It is not merely “emotionally intense”: it is the scene where the similarity profile shifts most sharply from control-related to devaluation-related signals, matching the narrative moment when the old dynamic visibly breaks down.

The second is ch096_s03 (“London Panic Dependency Fear and Sex as Last Weapon”), where the scene function shifts from the recognition of lost control to the search for a final lever. Here Mildred’s fear of losing security, her growing dependence, and her attempt to reactivate Philip through desire turn the chapter-scale crisis into a more specific local mechanism cluster. This scene is especially important for the localization of coercive control, but also remains high in trauma bonding and intermittent reinforcement. At the same time, the negative controls remain modest, indicating that the scene is not simply being rewarded for discursive complexity. The local coercive turn is also textually explicit: Mildred “fixed the catch on the wicket … so that Philip could not get in,” and the failed re-engagement soon escalates into “a furious torrent of abuse” after refusal (Maugham, 1915, ch. 96).

The final local scene in this chain is ch096_s04 (“Night Seduction Rejection and Narcissistic Rage”). This scene functions as the terminal rupture unit: confinement, soft seduction, refusal, and then full-scale abusive reversal. In chapter terms, Chapter 96 looks like one large high point; in scene terms, however, that high point is generated by at least three different local units: loss of leverage, panic-driven counter-move, and rage after failed recovery. This is precisely the kind of explanatory gain that scene-level decomposition provides.

**Figure 6:**
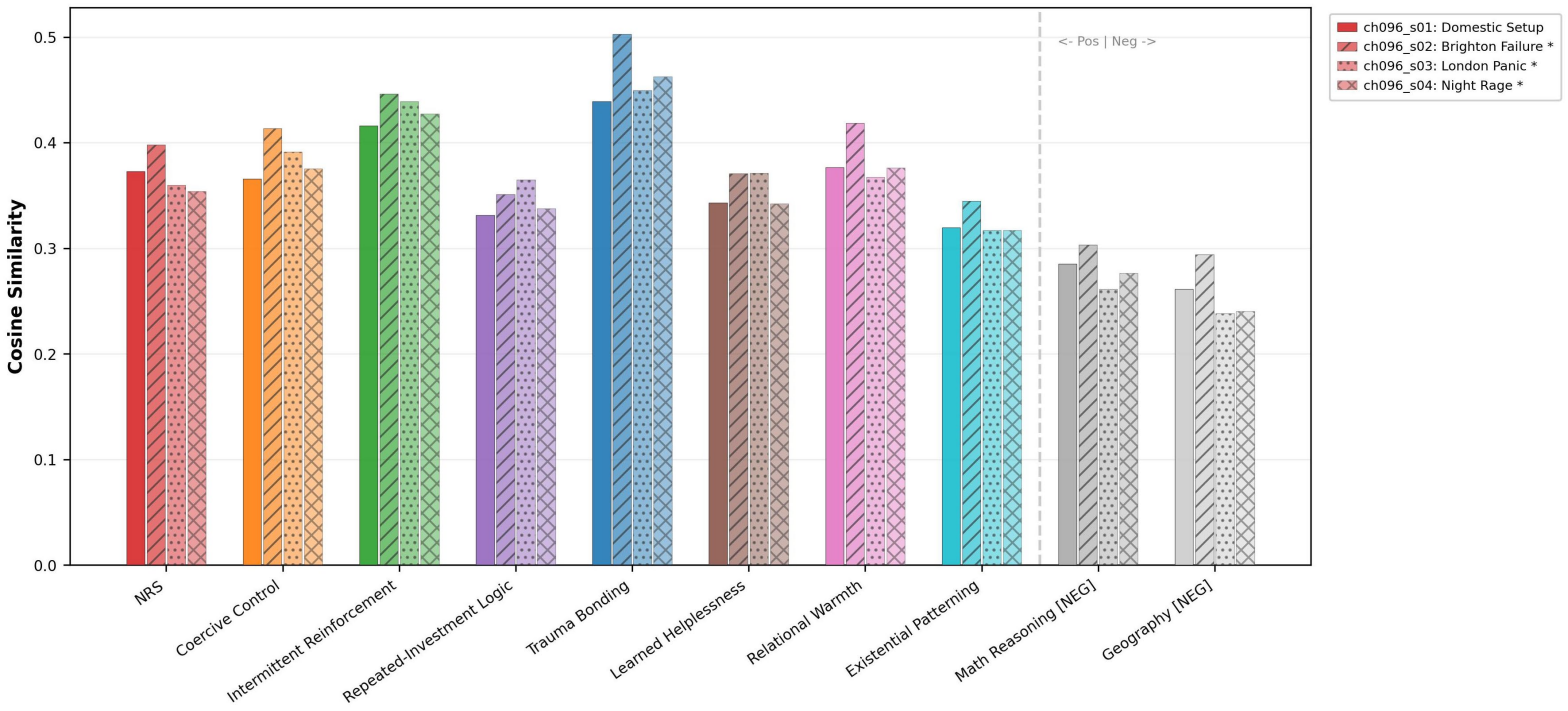
Scene-level decomposition of the chapter 96 cohabitation crisis. Scene-level construct profiles for the Chapter 96 crisis sequence, showing how the chapter peak is generated by multiple distinct local scenes rather than by uniform elevation across the chapter. At minimum, the figure distinguishes the Brighton failure/loss-of-leverage scene, the dependency-panic counter-move, and the rage-after-refusal scene.

The broader implication is methodological. The chapter-level signal around Chapter 96 is not an artifact of a long chapter with many vaguely similar passages. It is produced by stacked, narratively distinct local mechanism scenes, each of which carries a slightly different construct profile (see Figure 6).

#### 3.5.3 A5: poverty, collapse, and the Persian-rug reorientation

The second zone is the poverty-collapse and existential-reorientation sequence. At the chapter level this might look like one broad “late suffering” phase; the scene-level results split it into two distinct centers—one around degradation and helplessness, the other around pattern, meaning, and release.

The clearest poverty-collapse scene is ch100_s04 (“Sustained Deterioration and Limit”), which gathers the chapter’s helplessness and repeated-investment signals into one unit of exhaustion, hunger, and dwindling agency. It is the strongest local site for learned helplessness, and also scores high on repeated-investment logic and intermittent reinforcement. This scene is not about relational coercion; it shows that the anchors also register a broader collapse loop, in which Philip keeps acting under worsening prospects while losing effective agency.

The later philosophical turn is sharply localized in Chapter 106, in ch106_s02 and ch106_s03 (“Persian Rug Epiphany”). The first gathers mortality, futility, grief, and the question of meaning; the second states the solution—that life can be shaped as a design even if it has no ultimate purpose. Splitting the chapter into scenes keeps the existential turn from collapsing into one vague “philosophical chapter”: the peak comes from two distinct scenes, one of questioning and collapse and one of answer and restructuring. The wording is transparent, moving from “Life had no meaning” to the idea that one might still “make a design” out of experience (Maugham, 1915, ch. 106).

**Figure 7:**
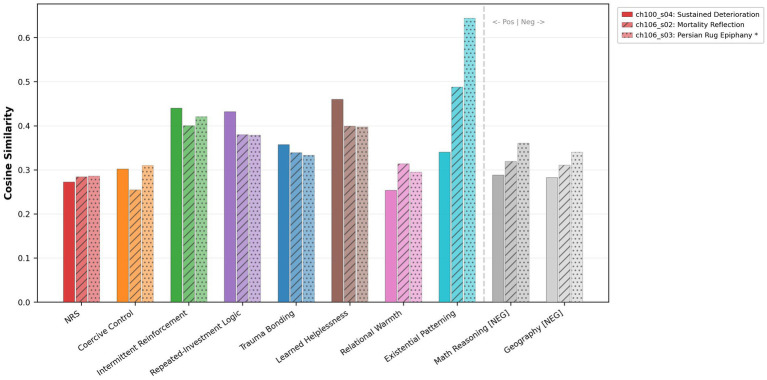
From poverty collapse to Persian-rug reorientation: local peak scenes in chapters 100 and 106. Scene-level contrast between the strongest helplessness/repeated-investment collapse scene (e.g., ch100_s04) and the existential-reorientation scenes around Chapter 106 (especially ch106_s02 and ch106_s03). The figure shows that the late-novel crisis is not semantically homogeneous but bifurcates into collapse and pattern-reorientation.

The Existential Patterning anchor shows its clearest local validity in these existential scenes. It is not a general “abstractness” detector; it rises most strongly where the Persian-rug solution is stated. This local concentration also explains its Test 3 behaviour: the Cronshaw signal need not form a smooth focal-arc trajectory to be valid, because its role is local concentration, not uniform spread (see Figure 7).

#### 3.5.4 A7–A8: pregnancy shock, residual bondage, and settlement

The final localization zone is the late Sally/settlement sequence, where the scene-level results complicate any simple reading of the ending as a clean replacement of one attachment by another.

The first key scene is ch121_s02 (“Pregnancy Shock, Panic, and Rationalized Escape”), whose profile is informatively mixed. It belongs to the Sally line but is not dominated by relational warmth; it combines panic, future planning, self-justification, and route-planning fantasy. This is exactly where the controls earn their place: the rise of geography/navigation here shows the scene is saturated with travel and future-movement language as well as interpersonal tension. Without that control, ch121_s02 might be over-read as another toxic-relational peak, when part of its signal is really escape-script and route-planning.

The final chapter then splits into two equally important local scenes. ch122_s01 functions as a residual-bondage trigger scene. On his way to meet Sally, Philip momentarily mistakes another woman for Mildred and is forced to admit that the old attachment remains active at some level. The local score pattern here is highly revealing: the strongest load falls not on coercion or explicit devaluation, but on intermittent reinforcement, trauma-bonding-related language, and associated residual dependence markers. This supports a crucial interpretive point: what survives at the end is not ongoing external control by Mildred, but an internalized reactive structure—the trace of the earlier bond. The textual surface is consistent with this interpretation: “Would he never be free from that passion?” (Maugham, 1915, ch. 122).

By contrast, ch122_s02 (“Recognition of True Desire, Proposal, and Settlement”) is an integration scene, not a simple “happy-love” scene. It contains Sally, the proposal, and settlement, but also the collapse of the travel plan, the rejection of abstract future-planning, and the return of Cronshaw-like pattern language to lived choice. That is why it stays mixed across constructs: residual attachment, ethical choice, and affiliative settlement coexist. Its center is domestic rather than romanticized—Philip recognizes his wish for “a wife and a home and love” (Maugham, 1915, ch. 122).

**Figure 8:**
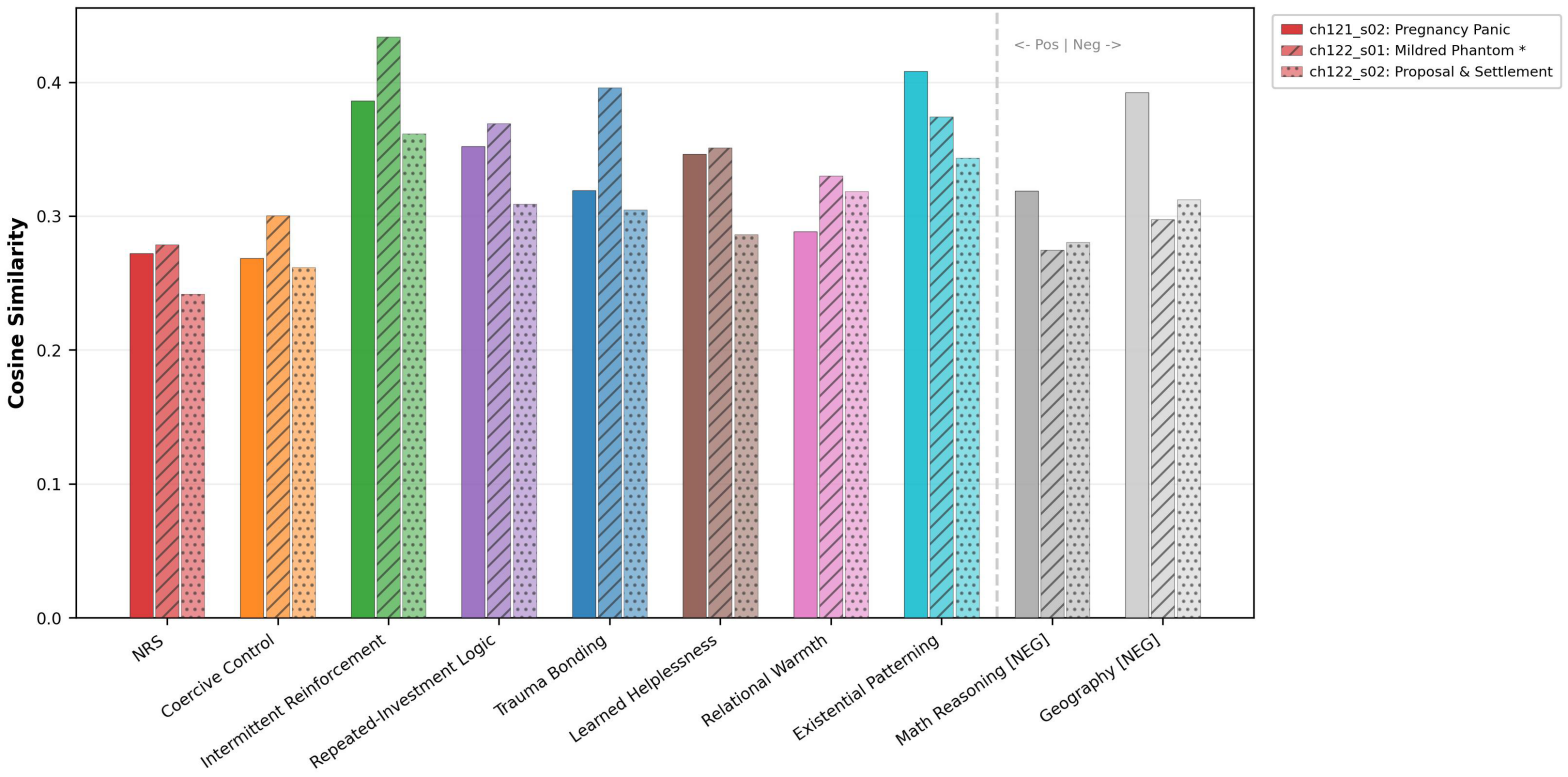
Residual reactivation and settlement in the late sally sequence. Scene-level construct profiles for ch121_s02, ch122_s01, and ch122_s02, showing that the ending is not a simple healthy replacement of the earlier bond. Instead, the final sequence combines future-script disruption, residual attachment reactivation, and affiliative settlement within a mixed but interpretable local pattern.

At scene scale the ending becomes more precise. Resolution does not mean the earlier bond is erased; the ending is built from two units—residual reactivation and repatterned acceptance—a distinction that chapter averages alone could not support (see Figure 8).

### 3.6 Cross-model robustness

To assess whether the patterns established under RQ1–RQ3 depend on a single embedding model, the same scene corpus was analyzed under all three models described in Section 2.4. Three broad findings recur across Qwen3-8B, MiniLM, and Qwen3-0.6B: the cohabitation crisis around Chapter 96 remains a major relational peak, the poverty–collapse sequence around Chapters 98–100 remains a major helplessness/repeated-investment zone, and the existential turn around Chapter 106 remains the main Existential Patterning peak. Across all three models, the negative controls do not reproduce the focal relational crisis arc but instead peak in reasoning-heavy or travel-oriented passages, extending the chapter-level negative-control results reported in Section 3.4.

What differs across models is not the existence of the major zones but the clarity of the boundaries between them. MiniLM preserves the broad narrative architecture but under-resolves finer interpersonal mechanism distinctions: the Relational Warmth anchor overlaps with non-reparative scenes, and the separation among NRS, coercive control, and trauma bonding is less clean. Qwen3-0.6B, by contrast, separates the positive anchors from the negative controls more sharply than the 8B model while preserving substantial rank-order agreement for central constructs such as Existential Patterning, Learned Helplessness, NRS, and Trauma Bonding. The standardized gap between the eight positive-anchor scores and the two negative-control scores, pooled across the 165 scenes, was largest for Qwen3-0.6B (*d* ≈ 1.7) and MiniLM (*d* ≈ 1.5), and smallest for the primary 8B model (*d* ≈ 0.7; Cohen’s *d* with pooled standard deviation). The smaller in-family model therefore does not generate an unrelated topology; it sharpens anchor–control separation relative to the primary model while keeping its rank order.

**Figure 9:**
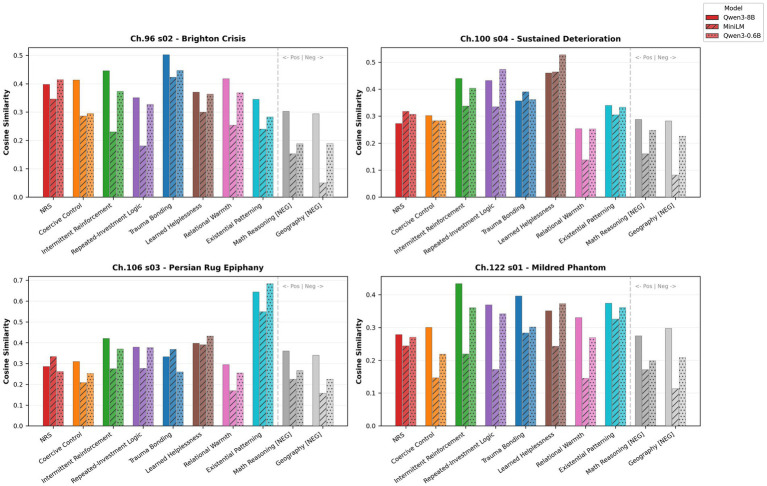
Cross-model robustness at major scene-level peaks. Cosine-similarity profiles at four representative peak scenes—the Chapter 96 cohabitation crisis (ch096_s02), the Chapter 100 poverty collapse (ch100_s04), the Chapter 106 existential turn (ch106_s03), and the Chapter 122 residual-attachment scene (ch122_s01)—compared across the three encoders (Qwen3-Embedding-8B, MiniLM, Qwen3-Embedding-0.6B), shown for all eight focal constructs and both negative-control domains. The relative construct profile recurs across all three models, while the negative control remains low in these relational scenes. Cross-model rank agreement for all eight positive constructs and both negative controls across the full 165-scene corpus is reported in Supplementary Appendix S3.

The full cross-model rank-agreement table is provided in Supplementary Appendix S3. In summary, rank agreement of the 165-scene construct profiles is positive for every construct and is higher within family (mean Spearman *ρ* = 0.73 for 8B vs. 0.6B) than across family (0.57 for 8B vs. MiniLM). The major zones recur in all three models: the poverty-collapse scene ch100_s04 and the existential scene ch106_s03 are each the top-ranked scene under all three encoders, and the Chapter 96 cohabitation scenes recur in the top five for trauma bonding and coercive control. The negative controls, by contrast, peak in reasoning-heavy chapters (67, 106) and travel chapters (121) under every model (Figure 9).

These results suggest that model scale does not monotonically improve construct discrimination for literary-scene probing. Larger models may maintain broader semantic integration, whereas smaller in-family models may produce harder local boundaries. This asymmetry indicates that different embedding scales yield different discrimination profiles, a methodological observation relevant to future work. Recovering the same construct architecture under the benchmark-leading 8B model—the most semantically integrated of the three—is therefore the conservative outcome; the sharper margins under the smaller in-family model reflect robustness to scale, not a flaw in the primary model.

### 3.7 Aggregation robustness (test 2 and test 3 under alternative pipelines)

To confirm that the chapter-level conclusions do not depend on the aggregation choices, Tests 2 and 3 are reported under four schemes: the published vector-level/trimmed-mean pipeline (VEC; the primary analysis of Tables 5, 6); similarity-level anchor aggregation (SL); plain-mean chunk pooling (MEAN); and pairwise chunk–anchor similarity (PAIR). The cached Qwen3-8B embeddings reproduce the released chapter similarities to within floating-point error, and the MEAN and PAIR schemes were obtained by re-aggregating those cached embeddings and recomputing the tests. The results are essentially invariant (Table 7). The same four constructs survive FDR correction in every scheme—NRS, Coercive Control, Intermittent Reinforcement, and Trauma Bonding—with effect sizes differing only in the second decimal; the negative controls never enter the significant set; and all eight positive constructs remain significantly autocorrelated in Test 3 under every scheme. As anticipated in Section 2.5, SL is numerically identical to VEC because the unit-normalized variants make the two differ only by a per-construct positive constant. Full per-construct tables (including Test 3) are provided in the Supplementary material.

**Table 7:** Test 2 effect sizes under four aggregation schemes (Hedges’ g; BH *q* in parentheses).

Construct	VEC (published scheme)	SL	MEAN	PAIR
Narcissistic relational style	+1.13 (0.009)*	+1.13 (0.009)*	+1.11 (0.010)*	+1.18 (0.005)*
Coercive control	+0.94 (0.021)*	+0.94 (0.021)*	+0.92 (0.021)*	+0.99 (0.010)*
Intermittent reinforcement	+0.81 (0.027)*	+0.81 (0.027)*	+0.79 (0.028)*	+0.91 (0.012)*
Trauma bonding	+0.87 (0.021)*	+0.87 (0.021)*	+0.86 (0.021)*	+0.90 (0.012)*
Repeated-investment logic	+0.60 (0.092)	+0.60 (0.092)	+0.59 (0.101)	+0.69 (0.053)
Relational warmth	+0.51 (0.150)	+0.51 (0.150)	+0.50 (0.155)	+0.53 (0.128)
Learned helplessness	+0.37 (0.285)	+0.37 (0.285)	+0.36 (0.296)	+0.44 (0.197)
Existential patterning	−0.14 (0.661)	−0.14 (0.661)	−0.16 (0.615)	−0.11 (0.727)

Mildred arc (*n* = 40) vs. resolution (*n* = 13). *Survives BH-FDR at *q* < 0.05. The significant set is identical across schemes. The VEC column is the published primary analysis and matches Table 5 exactly; SL is analytically identical to VEC (Section 2.5). MEAN and PAIR were obtained by re-aggregating the same cached embeddings under the alternative pooling schemes (10,000-permutation harness); their q-values differ from VEC only by aggregation scheme and permutation sampling.

### 3.8 Human-rater convergent validity

Six raters (R1–R6) independently rated the 20 blind passages on all ten rating dimensions (Section 2.7). Three indices are reported per dimension (Table 8): inter-rater reliability under both ICC (2,k) and Krippendorff’s *α* (ordinal); and convergent validity as the Spearman correlation between the mean human rating and the model cosine across the 20 passages. The two reliability indices index different properties: ICC (2,k) reports the reliability of the *mean* of six raters (incorporating the Spearman–Brown gain from averaging), whereas Krippendorff’s α reports per-rater agreement without averaging. We report both so that aggregate stability and per-rater concordance can be assessed independently.

**Table 8:** Human-rater reliability and convergent validity (six raters, R1–R6; 20 passages).

Construct	ICC (2,6)	*α* (ord.)	*ρ* (human, model)	95% CI	*p*
Narcissistic relational style	0.74	0.28	+0.72	[+0.40, +0.88]	<0.001
Coercive control	0.69	0.20	+0.65	[+0.25, +0.86]	0.003
Intermittent reinforcement	0.75	0.31	+0.28	[−0.13, +0.60]	0.228
Repeated-investment logic	0.67	0.23	+0.06	[−0.45, +0.53]	0.812
Trauma bonding	0.69	0.24	+0.67	[+0.26, +0.91]	0.002
Learned helplessness	0.46	0.09	+0.57	[+0.14, +0.84]	0.010
Relational warmth	0.83	0.42	−0.09	[−0.54, +0.40]	0.689
Existential patterning	0.86	0.46	+0.80	[+0.54, +0.93]	<0.001
Mathematical reasoning [NEG]	0.25	−0.03	+0.41	[−0.09, +0.78]	0.074
Geography/navigation [NEG]	0.49	0.15	+0.73	[+0.40, +0.90]	<0.001
Mean (positive constructs)	**0.71**	**0.28**	**+0.46**	**[+0.31, +0.54]**	**—**

ICC (2,6) is two-way random, average-measures, absolute-agreement reliability for the mean of six raters; Krippendorff’s α is ordinal-level per-rater agreement; ρ(human, model) is the Spearman correlation between the mean human rating and the model cosine across the 20 passages. 95% CIs are bootstrap intervals (10,000 passage resamples); *p* is a two-sided permutation test (10,000 permutations). The five focal constructs that reach significance are the NRS/Coercive Control/Trauma Bonding cluster that drives the Mildred Arc contrast, plus Learned Helplessness and Existential Patterning; the three non-significant rating dimensions (CIs spanning zero) are the broader anchors flagged for cautious interpretation. Bold values indicate Spearman correlations whose 95% bootstrap confidence intervals exclude zero and whose two-sided permutation *p*-values are below .05.

Because each scene was rated in isolation, the human study tests convergent validity at the scene level—whether an independent reader recognizes a construct within a single scene—rather than a construct’s overall presence across the novel. Across the eight focal constructs, the mean human rating agreed with the model scores at Spearman *ρ* = +0.46 (bootstrap 95% CI [+0.31, +0.54], excluding zero).

Five of the eight focal constructs showed individually significant convergent validity (two-sided permutation *p* < 0.05 with bootstrap CIs excluding zero; see Table 8): Existential Patterning (*ρ* = +0.80), NRS (+0.72), Trauma Bonding (+0.67), Coercive Control (+0.65), and Learned Helplessness (+0.57). The remaining three did not reach significance and their CIs span zero: Intermittent Reinforcement (*ρ* = +0.28), Repeated-Investment Logic (+0.06), and Relational Warmth (−0.09). Relational Warmth was, if anything, weakly negative. For all three, the divergence is not rater noise: inter-rater reliability remained moderate-to-high (ICC = 0.67–0.83; Table 8), so raters agreed with one another but tracked something different from the anchor.

Section 4.6 separates two diagnoses behind this pattern. Intermittent Reinforcement and Repeated-Investment Logic are *sequence-dependent* constructs—defined by patterns across many interactions—whose signal is strong and temporally organized at the chapter scale (Tests 2–3) but cannot be read from a single isolated, order-randomized scene. Their lower scene-level convergence is therefore a level-of-analysis effect rather than an invalid anchor. Relational Warmth shows the opposite pattern: reliable human agreement (ICC = 0.83) diverged from a stable model signal, pointing to an anchor semantic-boundary issue.

Accordingly, the human ratings converge with the model on three of the four constructs that drive the chapter-level Mildred Arc contrast (NRS, Coercive Control, Trauma Bonding), plus the Existential and Learned-Helplessness signals. The fourth, Intermittent Reinforcement, is robust in the computational analysis (Tables 5, 7) but is supported at the chapter/temporal scale rather than by the scene-level probe, and is interpreted with corresponding caution.

Inter-rater reliability for the six-rater mean was good for most focal constructs—ICC (2,6) from 0.46 (Learned Helplessness) to 0.86 (Existential Patterning), with bootstrap 95% CIs that excluded low values for the focal cluster (e.g., NRS [0.57, 0.82]; Trauma Bonding [0.37, 0.82]; Existential Patterning [0.64, 0.93]). Per-rater ordinal agreement was, as expected, lower and less precise (Krippendorff’s *α* from 0.09 to 0.46, with wide CIs that approached zero for the weakest constructs). Because the convergent-validity correlations use the *mean* of the six raters, ICC (2,6)—not α—is the reliability index relevant to them; α is reported for completeness. The convergent-validity CIs and permutation *p*-values are given in Table 8.

A separate matrix-alignment check addresses the concern that the four-construct co-elevation observed in the Mildred Arc could reflect rater confusion rather than shared structure. Specifically, the off-diagonal entries of the inter-construct correlation matrix (computed across the 20 passages) were compared between humans and model. The two matrices were positively aligned (Spearman *ρ* = +0.44, *p* = 0.018), indicating that the multi-construct co-occurrence pattern detected by the model is also visible in independent human judgment. Discriminant performance was likewise consistent: both humans and the model assigned low values on the two negative controls in the relational passages (model means: Math = 0.29, Geo = 0.28; mean across the six raters: Math = 0.38, Geo = 0.24). The positive human–model rank correlation for Geography/Navigation in Table 8 simply reflects that both humans and the model detect the few travel-saturated passages (e.g., the late escape-script scene); it does not indicate elevated absolute ratings, which remain low, so it does not undercut discriminant validity.

Robustness checks confirm that no single rater drives the result. Leave-one-out mean construct-level Spearman across the eight focal constructs ranged from +0.43 (dropping R2) to +0.49 (dropping R4), with the full six-rater set at +0.46.

A sensitivity subset restricted to raters with formal literary or translation training (R1, R2, R5; *n* = 3) yielded a nearly identical mean of +0.45, and a matrix-alignment *ρ* of +0.45 (*p* = 0.015), indicating that the validity estimates are robust across the full panel, leave-one-out checks, and the literary/translation-trained sensitivity subset. The human-rater results therefore provide graded rather than uniform support. They corroborate the chapter-level analysis most strongly for NRS, Coercive Control, Trauma Bonding, Learned Helplessness, and Existential Patterning—the constructs carrying the focal Mildred Arc cluster, the existential-reorientation signal, and the discriminant separation from negative-control domains—but weakly or not at all for Intermittent Reinforcement, Repeated-Investment Logic, and Relational Warmth, which should be interpreted most cautiously in subsequent applications.

## 4 Discussion

### 4.1 Summary of the main findings

At the chapter level, four constructs—NRS, coercive control, intermittent reinforcement, and trauma bonding—are consistently higher in the Mildred Arc than in the Resolution chapters. Together they form a non-random, time-organized relational cluster. At the scene level, those chapter peaks turn out to be produced by a few mechanism-bearing scenes rather than by uniform elevation. The chapter level captures the trajectory; the scene level captures where it is concentrated.

### 4.2 Pre-theoretical naturalism and the recoverability of later psychological constructs

One implication is historical, not just methodological. *Of Human Bondage* (1915) was written long before learned helplessness, trauma bonding, or coercive control were named in modern psychology. The results do not mean that Maugham anticipated those theories, and they do not license retrospective diagnosis. They suggest something more modest: later constructs may capture patterns of social and emotional life that are real enough to leave recognizable traces in a careful earlier observer’s prose.

We call this *pre-theoretical naturalistic convergence*, while noting a limit: the present design cannot fully separate genuine convergence from shared vocabulary (the constructs and the novel may simply use overlapping words). The novel does not “contain” these theories, but it portrays the interactions that later theory would name. The embedding results matter because they show that these patterns are not visible only through interpretive paraphrase—they also leave a measurable signature that theory-guided anchors can detect. The contribution is therefore not diagnosis but evidence that such mechanism patterns are plausibly present in richly observed fiction.

### 4.3 Mechanism clusters rather than isolated diagnostic constructs

A central lesson is that the strongest findings do not support eight independent “psychological meters.” The data make most sense as mechanism clusters.

At the chapter level, the Mildred Arc is marked by four constructs rising together—NRS, coercive control, intermittent reinforcement, and trauma bonding—not by any single construct. At the scene level these appear as overlapping but partly separable local mechanisms: some scenes stress humiliation and contempt, others leverage and dependence, others panic, others rage after a failed reconciliation. The later poverty-and-reorientation sequence forms a second cluster, in which helplessness, repeated-investment logic, and existential patterning (the “Cronshaw signal”; see Section 3.5) overlap without merging.

The key result is not that a preselected set of related labels all rose together, but that only a subset of a deliberately mixed battery separated the Mildred Arc from the Resolution chapters after correction. The Mildred Arc preferentially draws on one relational-process subcluster: degrading asymmetry, coercive leverage, intermittent re-engagement, and attachment under instability.

This cluster pattern is not a flaw in the method; it is part of the point. Literary narrative rarely builds each unit around one pure construct. Maugham braids several mechanisms together—humiliation with attraction, dependency with contempt, ethical reflection with self-deception, release with residue. A method that detects cluster-level organization is therefore better suited to fiction than one that assumes clean separation at every point.

### 4.4 Scale dependence: trajectories and local pulses

Comparing the two scales shows that scale is a substantive dimension, not just a convenience. At the chapter level, constructs form extended trajectories; at the scene level, those same trajectories resolve into local pulses produced by distinct scenes (Section 3.5). The practical implication is that computational literary psychology should ask not whether a construct is simply “present” or “absent,” but at what grain it is organized. Some constructs form smooth large-scale trajectories; others appear mainly as local pulses inside larger arcs. The distinction is narratological, not merely statistical.

### 4.5 Robustness, negative controls, and model scale

The negative controls and the cross-model comparison (Sections 3.4 and 3.6) strengthen the interpretation. The negative-control anchors do not follow the toxic-relational arc but respond to their own domains, which shows that the method is not simply rewarding abstract or dense prose. The cross-model results show that the broad structure is stable across all three models, yet a larger model does not automatically discriminate constructs better: the smaller in-family model produced sharper anchor–control margins, while the larger model kept broader semantic integration. Parameter count, in short, does not map straightforwardly onto interpretive usefulness.

### 4.6 Limitations

Several limitations should be acknowledged regarding the scope and design of the present study. All results derive from a single novel. This is appropriate for a theoretically saturated case study, but it limits generalizability. The analytic workflow itself is portable, but replication across additional long-form narratives would be needed before the present mechanism profiles can be treated as broader literary regularities. A particularly stringent next test would be to run the same battery on a control novel without a destructive attachment arc, in order to evaluate how specifically the observed pattern depends on the relational structure of “*Of Human Bondage*”. In addition, the semantic anchors were built from psychological theory and prior scholarship rather than from validated psychometric item sets, so the study is best understood as CCR-inspired rather than as a full psychometric implementation of CCR. The focal scene corpus was manually segmented on the basis of narrative-functional shifts. This affects the scene-level localization layer: although the segmentation is principled and documented, different but plausible scene boundaries could shift some local profiles.

The three constructs that did not converge with the human ratings fall under two diagnoses rather than a single weakness. Intermittent Reinforcement and Repeated-Investment Logic are defined by patterns *across* many interactions, which a single isolated scene can register only indirectly. Consistent with this, both remain robust at the chapter scale (Test 2 *g* = 0.81 and 0.60; Test 3 lag-1 *r* = 0.455 and 0.395, *p* < 0.001), so their weaker scene-level convergence reflects a level-of-analysis mismatch and calls for sequence-level human validation in future work. Relational Warmth is different: rater agreement was high (ICC = 0.83) yet diverged from a stable model signal. The chapter-level Warmth score correlates most strongly with the toxic-relational cluster (Warmth ~ Trauma Bonding *r* = +0.84; ~ NRS *r* = +0.73), indicating that the anchor also captures manipulative tenderness that readers decline to score as warmth—an anchor-specificity issue calling for a tighter anchor.

Finally, the human study is modest in scale (20 scenes, six raters) and targeted rather than representative in sampling, with wide confidence intervals; it is best read as a convergent-validity probe that triangulates the chapter-level findings, which rest on the permutation tests (Sections 3.1–3.3) and do not depend on the ratings. These limitations define the scope of the work: a theory-driven, multiscale literary-semantic case study.

## 5 Conclusion

The findings support a modest but durable claim: theory-informed anchors can show where a novel gives temporal and local form to devaluation, coercion, intermittent re-engagement, helplessness, and existential re-patterning. The method is a quantitative complement to close reading, not a replacement for it: it separates broad trajectories from local pulses, and relational crisis from reasoning- or travel-oriented passages.

More broadly, the study suggests that literary texts can serve as valuable sites for methodological experimentation in computational psychology, provided that the scope and strength of the claims remain proportionate to the design. The strongest contribution of the present work is therefore not clinical measurement, but multiscale semantic mapping: a way of showing how interpersonal mechanism clusters become narratively organized, locally instantiated, and methodologically testable within long-form fiction.

Philip’s bondage never announces itself in a single scene: it accumulates, wavers, and at last loosens into a chosen pattern. The method offered here is a way of seeing that movement whole, without mistaking the map for the mind.

## Data Availability

The data supporting the conclusions of this article are included in the article and Supplementary Material. These materials include the semantic anchor specifications, corpus and scene definitions, robustness tables, and human-rater validation materials. Additional supporting data will be made available by the corresponding author, without undue reservation.

## Supplementary material

SUPPLEMENTARY FIGURE S1 Scene-level construct heatmap (Qwen3-8B, standardized). Rows are the eight focal constructs; columns are the 165 scenes of the focal corpus (Chapters 55–122) in narrative order, with chapter starts marked on the horizontal axis. Color gives each scene's within-construct standardized similarity (z-score), so values are comparable along a row but not between rows. The major chapter-level peaks analyzed in Section 3.5 appear as localized bright bands rather than diffuse stretches: the early hooking scene ch055_s03 (Intermittent Reinforcement, Trauma Bonding, Repeated-Investment Logic, NRS); the Chapter 96 cohabitation-crisis scenes ch096_s02–s04 (Trauma Bonding, Coercive Control, NRS); the poverty-collapse scene ch100_s04 (Learned Helplessness, Repeated-Investment Logic); the existential-reorientation scenes ch106_s02–s03 (Existential Patterning; the brightest band in the figure); and the late residual-attachment and settlement scenes ch121_s02–ch122_s02. The figure provides a descriptive overview rather than a separate inferential result.
