# Supplementary figures and images for: Mapping relational mechanism clusters in Of Human Bondage: a theory-driven multiscale embedding analysis
Source: Front Psychol. 2026 Jul 16;17:1836153. doi: 10.3389/fpsyg.2026.1836153 (PMC13420877; doi:10.3389/fpsyg.2026.1836153)

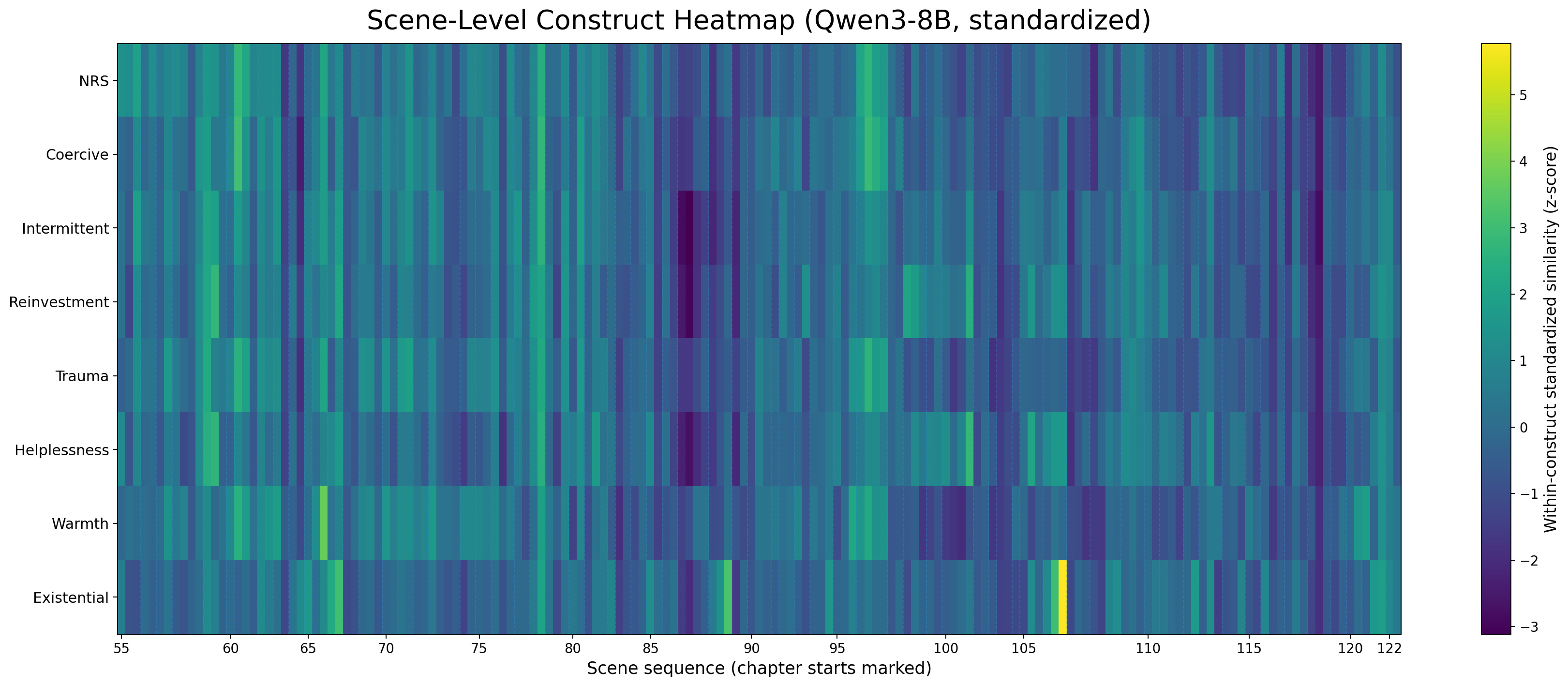

Supplement: SUPPLEMENTARY FIGURE S1 — Scene-level construct heatmap (Qwen3-8B, standardized). Rows are the eight focal constructs; columns are the 165 scenes of the focal corpus (Chapters 55–122) in narrative order, with chapter starts marked on the horizontal axis. Color gives each scene's within-construct standardized similarity (z-score), so values are comparable along a row but not between rows. The major chapter-level peaks analyzed in Section 3.5 appear as localized bright bands rather than diffuse stretches: the early hooking scene ch055_s03 (Intermittent Reinforcement, Trauma Bonding, Repeated-Investment Logic, NRS); the Chapter 96 cohabitation-crisis scenes ch096_s02–s04 (Trauma Bonding, Coercive Control, NRS); the poverty-collapse scene ch100_s04 (Learned Helplessness, Repeated-Investment Logic); the existential-reorientation scenes ch106_s02–s03 (Existential Patterning; the brightest band in the figure); and the late residual-attachment and settlement scenes ch121_s02–ch122_s02. The figure provides a descriptive overview rather than a separate inferential result. [file Image_1.JPEG]
