# Supplementary material for: Mapping relational mechanism clusters in Of Human Bondage: a theory-driven multiscale embedding analysis
Source: Front Psychol. 2026 Jul 16;17:1836153. doi: 10.3389/fpsyg.2026.1836153 (PMC13420877; doi:10.3389/fpsyg.2026.1836153)
Supplement: Supplementary file 2 [file Data_Sheet_1.ZIP › DataSheet_S1_HumanRater/Participant_Information_Sheet.docx]

**参与者知情信息说明书
Participant Information Sheet**

*研究题目 / Study Title:
面向计算文本分析校验的公开文学文本匿名人工标注研究
Anonymous Human Annotation of Published Literary Texts for Validation of Computational Text Analysis*

# 研究负责人 / Researcher

姓名 / Name: 赵政廷 Zhao Zhengting

单位 / Affiliation: 上海外国语大学高级翻译学院 Shanghai International Studies University, Graduate Institute of Interpretation and Translation

联系方式 / Contact: 02512@shisu.edu.cn

# 一、研究邀请 / Invitation

您好，感谢您考虑参与本项研究。本说明书将向您介绍本研究的目的、参与方式、可能涉及的事项与您的权利。请仔细阅读，并就任何不清楚的地方与研究者联系。您可以自主决定是否参与，亦可在任意阶段无条件退出，不会产生任何不利后果。

*Thank you for considering participation in this study. This sheet describes the purpose, procedure, and your rights as a participant. Please read it carefully and ask the researcher about anything that is unclear. Your participation is entirely voluntary, and you may withdraw at any time without any consequence.*

# 二、研究目的 / Purpose

本研究旨在为一项计算文本分析的研究结论提供独立的人工标注校验。具体而言，研究者使用语言模型嵌入（embedding）方法对公开出版的文学文本中若干叙事与心理学语义特征进行了量化分析；本人工标注环节用于检验上述计算结论是否与独立的人类阅读判断相一致。

*This study collects independent human ratings to validate the results of a computational text-analysis project. The computational component uses language-model embeddings to score narrative and psychological-semantic features in a published literary text; the present human-rating component asks independent adult readers to judge the same features so that computational and human judgments can be compared.*

# 三、您需要做什么 / What You Will Do

如果您同意参与，我们会请您完成以下事项：

- 阅读一份评分材料包（Rating Packet），其中包含来自一部公开出版小说的若干短篇段落。
- 针对每一段落，按 0–4 的整数评分量表，对若干叙事与心理学语义维度逐项打分。
- 评分材料分为两个 session，每个 session 10 段，中间可休息。
- 完成后将文档以 Rating_<您的编号>.docx 形式保存并回传给研究者。

*If you agree to participate, you will be asked to: read a rating packet containing short excerpts from a published novel; rate each passage on a 0–4 integer scale across several narrative and psychological-semantic dimensions; complete two sessions of ten passages each (with a break in between); and return the completed Word document to the researcher.*

# 四、所需时间 / Time Commitment

总计约 90–120 分钟，可分两次进行，无须一次完成。

*Approximately 90–120 minutes in total, which may be split across the two sessions and need not be completed in a single sitting.*

# 五、自愿参与与退出 / Voluntary Participation and Withdrawal

参与本研究完全自愿。您可以在阅读本说明书后选择不参与，也可在任何阶段——包括开始评分之后——选择停止并退出，无须说明理由，不会产生任何后果。如您在交回评分文件后希望撤回数据，请在数据交回后两周内联系研究者，研究者将删除您的全部评分数据。

*Participation is entirely voluntary. You may decline to participate or withdraw at any stage—including after beginning the rating—without giving a reason and without any consequence. If you wish to withdraw your data after returning the rating file, please contact the researcher within two weeks; your ratings will be deleted in full.*

# 六、可能的风险与收益 / Risks and Benefits

本研究属于最低风险研究。评分材料为公开出版小说中的段落，其中部分内容涉及人际冲突、心理痛苦或情绪强烈的情节描写。如果您在阅读过程中感到不适，可以随时暂停或退出。本研究不涉及任何临床干预、医学测试、生理记录、欺骗，亦不会评估或诊断您本人的心理健康状况。

本研究不向参与者提供任何直接利益，也不会给您带来法律、职业或人际方面的影响。您的参与有助于推进对长篇叙事文本的计算分析方法学研究。

*This is a minimal-risk study. The reading material consists of passages from a published novel; some passages describe interpersonal conflict, emotional distress, or psychologically intense scenes. You may pause or stop at any time if you feel uncomfortable. The study does not involve clinical intervention, medical testing, physiological recording, deception, or any assessment of your own mental health. There is no direct benefit to participants; your participation contributes to methodological research on computational analysis of long-form narrative text.*

# 七、数据处理与匿名 / Data Handling and Anonymity

- 您的评分以一个鉴别用的化名（如 R1、R2）记录，研究者持有的化名—姓名对照表将与评分数据分开保存，且不会出现在任何对外发表的文件中。
- 公开发表的版本将仅包含汇总后的统计指标（如 Spearman 相关系数、ICC 等），不会出现可识别任何参与者身份的内容。
- 您提供的人口学信息（性别、年龄区间、专业背景）仅用于在论文中以匿名方式描述评分者样本的整体构成，不会与具体评分挂钩。
- 原始评分数据将由研究者妥善保存至少 5 年，仅用于本研究及其后续学术发表。数据不会被出售、不会用于商业用途、亦不会与本研究目的之外的第三方共享。

*Your ratings are recorded under a pseudonym (e.g., R1, R2). The pseudonym–name lookup is held separately by the researcher and is not included in any published output. Published versions report only aggregated statistics (e.g., Spearman correlations, ICC) and contain no information that could identify any individual rater. Demographic information (gender, age band, professional background) is used only to describe the rater sample at the aggregate level and is not linked to specific ratings. Raw rating data will be stored securely by the researcher for at least five years and used solely for this research and related academic publication; data will not be sold, used commercially, or shared with third parties outside the stated research purpose.*

# 八、数据用途 / Use of Data

本研究的汇总性、匿名结果将用于学术论文发表、会议报告或方法学补充材料。为支持开放科学，汇总后的（不可识别个人的）评分数据可能随论文一并公开。您的原始单条评分、姓名、单位或其他可识别信息将不会被公开。

*Aggregated and anonymized results may appear in academic papers, conference reports, or methodological supplements. To support open science, the aggregated (non-identifiable) rating data may be made publicly available alongside the publication. No raw individual ratings, names, affiliations, or other identifying information will be released.*

# 九、联系方式 / Contact Information

如您对本研究有任何疑问、需要进一步说明，或希望反映任何与本研究相关的问题，请联系研究负责人：赵政廷（02512@shisu.edu.cn）。

如您希望就本研究的伦理事项向独立第三方进行咨询或投诉，可联系：上海外国语大学高级翻译学院科研伦理委员会，[联系电话 +86 21 35372991]。

*For questions about the study, please contact the researcher: Zhao Zhengting (02512@shisu.edu.cn). For independent enquiries or complaints regarding the ethics of this study, please contact: Research Ethics Committee, GIIT/SISU, Tel: +86 21 35372991.*

# 十、知情同意声明 / Consent Statement

请在阅读本说明书后，如同意参与，于下方签名或在回传研究者的邮件 / 信息中明确写出：「我已阅读并理解参与者知情信息说明书，自愿参与本研究」即可视为知情同意。本研究接受书面签字、电子签名或邮件 / 通讯软件文字确认三种形式。

*After reading this sheet, if you agree to participate, please sign below or include the following statement in your reply email/message to the researcher: "I have read and understood the Participant Information Sheet and voluntarily agree to participate in this study." Written signature, electronic signature, or written email/message confirmation are all accepted as evidence of informed consent.*

参与者签字 / Participant signature: ____________________________

日期 / Date: ____________________

研究者签字 / Researcher signature: ____________________________

日期 / Date: ____________________
