# Supplementary material for: Mapping relational mechanism clusters in Of Human Bondage: a theory-driven multiscale embedding analysis
Source: Front Psychol. 2026 Jul 16;17:1836153. doi: 10.3389/fpsyg.2026.1836153 (PMC13420877; doi:10.3389/fpsyg.2026.1836153)
Supplement: Supplementary file 2 [file Data_Sheet_1.ZIP › DataSheet_S1_HumanRater/Rating_Packet_English_blind.docx]

Scene Rating — English Scoring Packet

| **Rater ID** |  |
| --- | --- |
| **Background (psychology / literature)** |  |
| **Date** |  |

# Instructions

- Rate each passage on how strongly each construct is present IN THAT PASSAGE.
- Score the ENGLISH passages in this file.
- Scale 0–4: 0 = not present · 1 = slight · 2 = moderate · 3 = strong · 4 = central/defining.
- Rate each construct INDEPENDENTLY. A passage may be high on several, on one, or on NONE — many will be 0 on most constructs. If a construct does not apply, score 0.
- Type a single digit 0–4 in each shaded Score cell. Work alone; there are two sessions — take a break between them.
- When finished, save as Rating_<YourID>.docx and return the file.

# Construct definitions

| **Construct** | **Definition** |
| --- | --- |
| **Narcissistic Relational Style (NRS)** | One person treats the other with grandiosity, entitlement, contempt, or exploitation, showing little empathy; devalues, humiliates, or uses the other. |
| **Coercive Control (Coercive)** | One person restricts the other’s freedom through pressure, monitoring, intimidation, threats, isolation, or rules; the other’s options shrink. |
| **Intermittent Reinforcement (IntReinf)** | Unpredictable alternation of warmth and rejection: rare rewards after stretches of coldness keep the other hooked and waiting. |
| **Repeated-Investment Logic (RepInvest)** | Continuing to pour time, emotion, or sacrifice into a worsening or unrewarding situation, expecting it will finally pay off (sunk cost). |
| **Trauma Bonding (TraumaBond)** | A strong attachment that persists despite mistreatment; harm and occasional kindness are entangled, with self-blame and rationalizing. |
| **Learned Helplessness (Helpless)** | Repeated uncontrollable setbacks lead to giving up, passivity, resignation, loss of agency; effort feels pointless. |
| **Relational Warmth (Warmth)** | Mutual care, respect, trust, safety, and reciprocity; a steady, supportive connection. |
| **Existential Patterning (Existential)** | Reflection on meaning or meaninglessness; making a “pattern” or design out of life; detachment, acceptance. |
| **Mathematical Reasoning (Math)** | The passage is centrally about formal/abstract reasoning, calculation, logic, or proof. |
| **Geography / Navigation (Geo)** | The passage is centrally about travel, routes, maps, directions, places, or movement through space. |

## How to tell similar constructs apart

- **IntReinf —** Score only when reward/warmth and rejection alternate UNPREDICTABLY (not steady warmth or steady coldness).
- **TraumaBond —** Requires attachment that PERSISTS despite harm, with rationalizing or self-blame — not mere conflict.
- **RepInvest —** Requires continued or escalating investment / sunk-cost logic ("I have put in so much, I must go on").
- **Warmth —** High ONLY for mutual, respectful, safe connection. Do NOT score one-sided infatuation or tenderness inside a manipulative dynamic.

# Session 1

## Passage P01

He realised that he had deceived himself; it was no self-sacrifice that had driven him to think of marrying, but the desire for a wife and a home and love; and now that it all seemed to slip through his fingers he was seized with despair. He wanted all that more than anything in the world. What did he care for Spain and its cities, Cordova, Toledo, Leon; what to him were the pagodas of Burmah and the lagoons of South Sea Islands? America was here and now. It seemed to him that all his life he had followed the ideals that other people, by their words or their writings, had instilled into him, and never the desires of his own heart. Always his course had been swayed by what he thought he should do and never by what he wanted with his whole soul to do. He put all that aside now with a gesture of impatience. He had lived always in the future, and the present always, always had slipped through his fingers. His ideals? He thought of his desire to make a design, intricate and beautiful, out of the myriad, meaningless facts of life: had he not seen also that the simplest pattern, that in which a man was born, worked, married, had children, and died, was likewise the most perfect? It might be that to surrender to happiness was to accept defeat, but it was a defeat better than many victories.

He glanced quickly at Sally, he wondered what she was thinking, and then looked away again.

“I was going to ask you to marry me,” he said.

“I thought p’raps you might, but I shouldn’t have liked to stand in your way.”

“You wouldn’t have done that.”

“How about your travels, Spain and all that?”

“How d’you know I want to travel?”

“I ought to know something about it. I’ve heard you and Dad talk about it till you were blue in the face.”

“I don’t care a damn about all that.” He paused for an instant and then spoke in a low, hoarse whisper. “I don’t want to leave you! I can’t leave you.”

She did not answer. He could not tell what she thought.

“I wonder if you’ll marry me, Sally.”

She did not move and there was no flicker of emotion on her face, but she did not look at him when she answered.

“If you like.”

“Don’t you want to?”

“Oh, of course I’d like to have a house of my own, and it’s about time I was settling down.”

He smiled a little. He knew her pretty well by now, and her manner did not surprise him.

“But don’t you want to marry ME?”

“There’s no one else I would marry.”

“Then that settles it.”

“Mother and Dad will be surprised, won’t they?”

“I’m so happy.”

“I want my lunch,” she said.

“Dear!”

He smiled and took her hand and pressed it. They got up and walked out of the gallery. They stood for a moment at the balustrade and looked at Trafalgar Square. Cabs and omnibuses hurried to and fro, and crowds passed, hastening in every direction, and the sun was shining.

| **P01** | **Score (0–4)** |
| --- | --- |
| Narcissistic Relational Style (NRS) |  |
| Coercive Control (Coercive) |  |
| Intermittent Reinforcement (IntReinf) |  |
| Repeated-Investment Logic (RepInvest) |  |
| Trauma Bonding (TraumaBond) |  |
| Learned Helplessness (Helpless) |  |
| Relational Warmth (Warmth) |  |
| Existential Patterning (Existential) |  |
| Mathematical Reasoning (Math) |  |
| Geography / Navigation (Geo) |  |

## Passage P02

For the next three months Philip worked on subjects which were new to him. The unwieldy crowd which had entered the Medical School nearly two years before had thinned out: some had left the hospital, finding the examinations more difficult to pass than they expected, some had been taken away by parents who had not foreseen the expense of life in London, and some had drifted away to other callings. One youth whom Philip knew had devised an ingenious plan to make money; he had bought things at sales and pawned them, but presently found it more profitable to pawn goods bought on credit; and it had caused a little excitement at the hospital when someone pointed out his name in police-court proceedings. There had been a remand, then assurances on the part of a harassed father, and the young man had gone out to bear the White Man’s Burden overseas. The imagination of another, a lad who had never before been in a town at all, fell to the glamour of music-halls and bar parlours; he spent his time among racing-men, tipsters, and trainers, and now was become a book-maker’s clerk. Philip had seen him once in a bar near Piccadilly Circus in a tight-waisted coat and a brown hat with a broad, flat brim. A third, with a gift for singing and mimicry, who had achieved success at the smoking concerts of the Medical School by his imitation of notorious comedians, had abandoned the hospital for the chorus of a musical comedy. Still another, and he interested Philip because his uncouth manner and interjectional speech did not suggest that he was capable of any deep emotion, had felt himself stifle among the houses of London. He grew haggard in shut-in spaces, and the soul he knew not he possessed struggled like a sparrow held in the hand, with little frightened gasps and a quick palpitation of the heart: he yearned for the broad skies and the open, desolate places among which his childhood had been spent; and he walked off one day, without a word to anybody, between one lecture and another; and the next thing his friends heard was that he had thrown up medicine and was working on a farm.

Philip attended now lectures on medicine and on surgery. On certain mornings in the week he practised bandaging on out-patients glad to earn a little money, and he was taught auscultation and how to use the stethoscope. He learned dispensing. He was taking the examination in Materia Medica in July, and it amused him to play with various drugs, concocting mixtures, rolling pills, and making ointments. He seized avidly upon anything from which he could extract a suggestion of human interest.

| **P02** | **Score (0–4)** |
| --- | --- |
| Narcissistic Relational Style (NRS) |  |
| Coercive Control (Coercive) |  |
| Intermittent Reinforcement (IntReinf) |  |
| Repeated-Investment Logic (RepInvest) |  |
| Trauma Bonding (TraumaBond) |  |
| Learned Helplessness (Helpless) |  |
| Relational Warmth (Warmth) |  |
| Existential Patterning (Existential) |  |
| Mathematical Reasoning (Math) |  |
| Geography / Navigation (Geo) |  |

## Passage P03

Philip went to see her every day. She took the medicine he had prescribed and followed his directions, and soon the results were so apparent that she gained the greatest confidence in Philip’s skill. As she grew better she grew less despondent. She talked more freely.

“As soon as I can get a job I shall be all right,” she said. “I’ve had my lesson now and I mean to profit by it. No more racketing about for yours truly.”

Each time he saw her, Philip asked whether she had found work. She told him not to worry, she would find something to do as soon as she wanted it; she had several strings to her bow; it was all the better not to do anything for a week or two. He could not deny this, but at the end of that time he became more insistent. She laughed at him, she was much more cheerful now, and said he was a fussy old thing. She told him long stories of the manageresses she interviewed, for her idea was to get work at some eating-house; what they said and what she answered. Nothing definite was fixed, but she was sure to settle something at the beginning of the following week: there was no use hurrying, and it would be a mistake to take something unsuitable.

“It’s absurd to talk like that,” he said impatiently. “You must take anything you can get. I can’t help you, and your money won’t last for ever.”

“Oh, well, I’ve not come to the end of it yet and chance it.”

He looked at her sharply. It was three weeks since his first visit, and she had then less than seven pounds. Suspicion seized him. He remembered some of the things she had said. He put two and two together. He wondered whether she had made any attempt to find work. Perhaps she had been lying to him all the time. It was very strange that her money should have lasted so long.

“What is your rent here?”

“Oh, the landlady’s very nice, different from what some of them are; she’s quite willing to wait till it’s convenient for me to pay.”

He was silent. What he suspected was so horrible that he hesitated. It was no use to ask her, she would deny everything; if he wanted to know he must find out for himself. He was in the habit of leaving her every evening at eight, and when the clock struck he got up; but instead of going back to Harrington Street he stationed himself at the corner of Fitzroy Square so that he could see anyone who came along William Street. It seemed to him that he waited an interminable time, and he was on the point of going away, thinking his surmise had been mistaken, when the door of No. 7 opened and Mildred came out. He fell back into the darkness and watched her walk towards him. She had on the hat with a quantity of feathers on it which he had seen in her room, and she wore a dress he recognized, too showy for the street and unsuitable to the time of year. He followed her slowly till she came into the Tottenham Court Road, where she slackened her pace; at the corner of Oxford Street she stopped, looked round, and crossed over to a music-hall. He went up to her and touched her on the arm. He saw that she had rouged her cheeks and painted her lips.

“Where are you going, Mildred?”

She started at the sound of his voice and reddened as she always did when she was caught in a lie; then the flash of anger which he knew so well came into her eyes as she instinctively sought to defend herself by abuse. But she did not say the words which were on the tip of her tongue.

“Oh, I was only going to see the show. It gives me the hump sitting every night by myself.”

He did not pretend to believe her.

“You mustn’t. Good heavens, I’ve told you fifty times how dangerous it is. You must stop this sort of thing at once.”

“Oh, hold your jaw,” she cried roughly. “How d’you suppose I’m going to live?”

He took hold of her arm and without thinking what he was doing tried to drag her away.

“For God’s sake come along. Let me take you home. You don’t know what you’re doing. It’s criminal.”

“What do I care? Let them take their chance. Men haven’t been so good to me that I need bother my head about them.”

She pushed him away and walking up to the box-office put down her money. Philip had threepence in his pocket. He could not follow. He turned away and walked slowly down Oxford Street.

“I can’t do anything more,” he said to himself.

That was the end. He did not see her again.

| **P03** | **Score (0–4)** |
| --- | --- |
| Narcissistic Relational Style (NRS) |  |
| Coercive Control (Coercive) |  |
| Intermittent Reinforcement (IntReinf) |  |
| Repeated-Investment Logic (RepInvest) |  |
| Trauma Bonding (TraumaBond) |  |
| Learned Helplessness (Helpless) |  |
| Relational Warmth (Warmth) |  |
| Existential Patterning (Existential) |  |
| Mathematical Reasoning (Math) |  |
| Geography / Navigation (Geo) |  |

## Passage P04

But about three in the morning Philip awoke and could not sleep again. He began to think of Mildred. He tried not to, but could not help himself. He repeated to himself the same thing time after time till his brain reeled. It was inevitable that she should marry: life was hard for a girl who had to earn her own living; and if she found someone who could give her a comfortable home she should not be blamed if she accepted. Philip acknowledged that from her point of view it would have been madness to marry him: only love could have made such poverty bearable, and she did not love him. It was no fault of hers; it was a fact that must be accepted like any other. Philip tried to reason with himself. He told himself that deep down in his heart was mortified pride; his passion had begun in wounded vanity, and it was this at bottom which caused now a great part of his wretchedness. He despised himself as much as he despised her. Then he made plans for the future, the same plans over and over again, interrupted by recollections of kisses on her soft pale cheek and by the sound of her voice with its trailing accent; he had a great deal of work to do, since in the summer he was taking chemistry as well as the two examinations he had failed in. He had separated himself from his friends at the hospital, but now he wanted companionship. There was one happy occurrence: Hayward a fortnight before had written to say that he was passing through London and had asked him to dinner; but Philip, unwilling to be bothered, had refused. He was coming back for the season, and Philip made up his mind to write to him.

He was thankful when eight o’clock struck and he could get up. He was pale and weary. But when he had bathed, dressed, and had breakfast, he felt himself joined up again with the world at large; and his pain was a little easier to bear. He did not feel like going to lectures that morning, but went instead to the Army and Navy Stores to buy Mildred a wedding-present. After much wavering he settled on a dressing-bag. It cost twenty pounds, which was much more than he could afford, but it was showy and vulgar: he knew she would be aware exactly how much it cost; he got a melancholy satisfaction in choosing a gift which would give her pleasure and at the same time indicate for himself the contempt he had for her.

| **P04** | **Score (0–4)** |
| --- | --- |
| Narcissistic Relational Style (NRS) |  |
| Coercive Control (Coercive) |  |
| Intermittent Reinforcement (IntReinf) |  |
| Repeated-Investment Logic (RepInvest) |  |
| Trauma Bonding (TraumaBond) |  |
| Learned Helplessness (Helpless) |  |
| Relational Warmth (Warmth) |  |
| Existential Patterning (Existential) |  |
| Mathematical Reasoning (Math) |  |
| Geography / Navigation (Geo) |  |

## Passage P05

One evening, at the beginning of February, Philip told her that he was dining with Lawson, who was giving a party in his studio to celebrate his birthday; and he would not be in till late; Lawson had bought a couple of bottles of the punch they favoured from the tavern in Beak Street, and they proposed to have a merry evening. Mildred asked if there were going to be women there, but Philip told her there were not; only men had been invited; and they were just going to sit and talk and smoke: Mildred did not think it sounded very amusing; if she were a painter she would have half a dozen models about. She went to bed, but could not sleep, and presently an idea struck her; she got up and fixed the catch on the wicket at the landing, so that Philip could not get in. He came back about one, and she heard him curse when he found that the wicket was closed. She got out of bed and opened.

“Why on earth did you shut yourself in? I’m sorry I’ve dragged you out of bed.”

“I left it open on purpose, I can’t think how it came to be shut.”

“Hurry up and get back to bed, or you’ll catch cold.”

He walked into the sitting-room and turned up the gas. She followed him in. She went up to the fire.

“I want to warm my feet a bit. They’re like ice.”

He sat down and began to take off his boots. His eyes were shining and his cheeks were flushed. She thought he had been drinking.

“Have you been enjoying yourself?” she asked, with a smile.

“Yes, I’ve had a ripping time.”

Philip was quite sober, but he had been talking and laughing, and he was excited still. An evening of that sort reminded him of the old days in Paris. He was in high spirits. He took his pipe out of his pocket and filled it.

“Aren’t you going to bed?” she asked.

“Not yet, I’m not a bit sleepy. Lawson was in great form. He talked sixteen to the dozen from the moment I got there till the moment I left.”

“What did you talk about?”

“Heaven knows! Of every subject under the sun. You should have seen us all shouting at the tops of our voices and nobody listening.”

Philip laughed with pleasure at the recollection, and Mildred laughed too. She was pretty sure he had drunk more than was good for him. That was exactly what she had expected. She knew men.

“Can I sit down?” she said.

Before he could answer she settled herself on his knees.

“If you’re not going to bed you’d better go and put on a dressing-gown.”

“Oh, I’m all right as I am.” Then putting her arms round his neck, she placed her face against his and said: “Why are you so horrid to me, Phil?”

He tried to get up, but she would not let him.

“I do love you, Philip,” she said.

“Don’t talk damned rot.”

“It isn’t, it’s true. I can’t live without you. I want you.”

He released himself from her arms.

“Please get up. You’re making a fool of yourself and you’re making me feel a perfect idiot.”

“I love you, Philip. I want to make up for all the harm I did you. I can’t go on like this, it’s not in human nature.”

He slipped out of the chair and left her in it.

“I’m very sorry, but it’s too late.”

She gave a heart-rending sob.

“But why? How can you be so cruel?”

“I suppose it’s because I loved you too much. I wore the passion out. The thought of anything of that sort horrifies me. I can’t look at you now without thinking of Emil and Griffiths. One can’t help those things, I suppose it’s just nerves.”

She seized his hand and covered it with kisses.

“Don’t,” he cried.

She sank back into the chair.

“I can’t go on like this. If you won’t love me, I’d rather go away.”

“Don’t be foolish, you haven’t anywhere to go. You can stay here as long as you like, but it must be on the definite understanding that we’re friends and nothing more.”

Then she dropped suddenly the vehemence of passion and gave a soft, insinuating laugh. She sidled up to Philip and put her arms round him. She made her voice low and wheedling.

“Don’t be such an old silly. I believe you’re nervous. You don’t know how nice I can be.”

She put her face against his and rubbed his cheek with hers. To Philip her smile was an abominable leer, and the suggestive glitter of her eyes filled him with horror. He drew back instinctively.

“I won’t,” he said.

But she would not let him go. She sought his mouth with her lips. He took her hands and tore them roughly apart and pushed her away.

“You disgust me,” he said.

“Me?”

She steadied herself with one hand on the chimney-piece. She looked at him for an instant, and two red spots suddenly appeared on her cheeks. She gave a shrill, angry laugh.

“I disgust YOU.”

She paused and drew in her breath sharply. Then she burst into a furious torrent of abuse. She shouted at the top of her voice. She called him every foul name she could think of. She used language so obscene that Philip was astounded; she was always so anxious to be refined, so shocked by coarseness, that it had never occurred to him that she knew the words she used now. She came up to him and thrust her face in his. It was distorted with passion, and in her tumultuous speech the spittle dribbled over her lips.

“I never cared for you, not once, I was making a fool of you always, you bored me, you bored me stiff, and I hated you, I would never have let you touch me only for the money, and it used to make me sick when I had to let you kiss me. We laughed at you, Griffiths and me, we laughed because you was such a mug. A mug! A mug!”

Then she burst again into abominable invective. She accused him of every mean fault; she said he was stingy, she said he was dull, she said he was vain, selfish; she cast virulent ridicule on everything upon which he was most sensitive. And at last she turned to go. She kept on, with hysterical violence, shouting at him an opprobrious, filthy epithet. She seized the handle of the door and flung it open. Then she turned round and hurled at him the injury which she knew was the only one that really touched him. She threw into the word all the malice and all the venom of which she was capable. She flung it at him as though it were a blow.

“Cripple!”

| **P05** | **Score (0–4)** |
| --- | --- |
| Narcissistic Relational Style (NRS) |  |
| Coercive Control (Coercive) |  |
| Intermittent Reinforcement (IntReinf) |  |
| Repeated-Investment Logic (RepInvest) |  |
| Trauma Bonding (TraumaBond) |  |
| Learned Helplessness (Helpless) |  |
| Relational Warmth (Warmth) |  |
| Existential Patterning (Existential) |  |
| Mathematical Reasoning (Math) |  |
| Geography / Navigation (Geo) |  |

## Passage P06

Thinking of Cronshaw, Philip remembered the Persian rug which he had given him, telling him that it offered an answer to his question upon the meaning of life; and suddenly the answer occurred to him: he chuckled: now that he had it, it was like one of the puzzles which you worry over till you are shown the solution and then cannot imagine how it could ever have escaped you. The answer was obvious. Life had no meaning. On the earth, satellite of a star speeding through space, living things had arisen under the influence of conditions which were part of the planet’s history; and as there had been a beginning of life upon it so, under the influence of other conditions, there would be an end: man, no more significant than other forms of life, had come not as the climax of creation but as a physical reaction to the environment. Philip remembered the story of the Eastern King who, desiring to know the history of man, was brought by a sage five hundred volumes; busy with affairs of state, he bade him go and condense it; in twenty years the sage returned and his history now was in no more than fifty volumes, but the King, too old then to read so many ponderous tomes, bade him go and shorten it once more; twenty years passed again and the sage, old and gray, brought a single book in which was the knowledge the King had sought; but the King lay on his death-bed, and he had no time to read even that; and then the sage gave him the history of man in a single line; it was this: he was born, he suffered, and he died. There was no meaning in life, and man by living served no end. It was immaterial whether he was born or not born, whether he lived or ceased to live. Life was insignificant and death without consequence. Philip exulted, as he had exulted in his boyhood when the weight of a belief in God was lifted from his shoulders: it seemed to him that the last burden of responsibility was taken from him; and for the first time he was utterly free. His insignificance was turned to power, and he felt himself suddenly equal with the cruel fate which had seemed to persecute him; for, if life was meaningless, the world was robbed of its cruelty. What he did or left undone did not matter. Failure was unimportant and success amounted to nothing. He was the most inconsiderate creature in that swarming mass of mankind which for a brief space occupied the surface of the earth; and he was almighty because he had wrenched from chaos the secret of its nothingness. Thoughts came tumbling over one another in Philip’s eager fancy, and he took long breaths of joyous satisfaction. He felt inclined to leap and sing. He had not been so happy for months.

“Oh, life,” he cried in his heart, “Oh life, where is thy sting?”

For the same uprush of fancy which had shown him with all the force of mathematical demonstration that life had no meaning, brought with it another idea; and that was why Cronshaw, he imagined, had given him the Persian rug. As the weaver elaborated his pattern for no end but the pleasure of his aesthetic sense, so might a man live his life, or if one was forced to believe that his actions were outside his choosing, so might a man look at his life, that it made a pattern. There was as little need to do this as there was use. It was merely something he did for his own pleasure. Out of the manifold events of his life, his deeds, his feelings, his thoughts, he might make a design, regular, elaborate, complicated, or beautiful; and though it might be no more than an illusion that he had the power of selection, though it might be no more than a fantastic legerdemain in which appearances were interwoven with moonbeams, that did not matter: it seemed, and so to him it was. In the vast warp of life (a river arising from no spring and flowing endlessly to no sea), with the background to his fancies that there was no meaning and that nothing was important, a man might get a personal satisfaction in selecting the various strands that worked out the pattern. There was one pattern, the most obvious, perfect, and beautiful, in which a man was born, grew to manhood, married, produced children, toiled for his bread, and died; but there were others, intricate and wonderful, in which happiness did not enter and in which success was not attempted; and in them might be discovered a more troubling grace. Some lives, and Hayward’s was among them, the blind indifference of chance cut off while the design was still imperfect; and then the solace was comfortable that it did not matter; other lives, such as Cronshaw’s, offered a pattern which was difficult to follow, the point of view had to be shifted and old standards had to be altered before one could understand that such a life was its own justification. Philip thought that in throwing over the desire for happiness he was casting aside the last of his illusions. His life had seemed horrible when it was measured by its happiness, but now he seemed to gather strength as he realised that it might be measured by something else. Happiness mattered as little as pain. They came in, both of them, as all the other details of his life came in, to the elaboration of the design. He seemed for an instant to stand above the accidents of his existence, and he felt that they could not affect him again as they had done before. Whatever happened to him now would be one more motive to add to the complexity of the pattern, and when the end approached he would rejoice in its completion. It would be a work of art, and it would be none the less beautiful because he alone knew of its existence, and with his death it would at once cease to be.

Philip was happy.

| **P06** | **Score (0–4)** |
| --- | --- |
| Narcissistic Relational Style (NRS) |  |
| Coercive Control (Coercive) |  |
| Intermittent Reinforcement (IntReinf) |  |
| Repeated-Investment Logic (RepInvest) |  |
| Trauma Bonding (TraumaBond) |  |
| Learned Helplessness (Helpless) |  |
| Relational Warmth (Warmth) |  |
| Existential Patterning (Existential) |  |
| Mathematical Reasoning (Math) |  |
| Geography / Navigation (Geo) |  |

## Passage P07

Dropping the subject, he began to talk of his own youth; he had been in the Royal Navy, and it was his long connection with the sea that, when he retired, had made him settle at Farnley. He told Philip of old days in the Pacific and of wild adventures in China. He had taken part in an expedition against the head-hunters of Borneo and had known Samoa when it was still an independent state. He had touched at coral islands. Philip listened to him entranced. Little by little he told Philip about himself. Doctor South was a widower, his wife had died thirty years before, and his daughter had married a farmer in Rhodesia; he had quarrelled with him, and she had not come to England for ten years. It was just as if he had never had wife or child. He was very lonely. His gruffness was little more than a protection which he wore to hide a complete disillusionment; and to Philip it seemed tragic to see him just waiting for death, not impatiently, but rather with loathing for it, hating old age and unable to resign himself to its limitations, and yet with the feeling that death was the only solution of the bitterness of his life. Philip crossed his path, and the natural affection which long separation from his daughter had killed—she had taken her husband’s part in the quarrel and her children he had never seen—settled itself upon Philip. At first it made him angry, he told himself it was a sign of dotage; but there was something in Philip that attracted him, and he found himself smiling at him he knew not why. Philip did not bore him. Once or twice he put his hand on his shoulder: it was as near a caress as he had got since his daughter left England so many years before. When the time came for Philip to go Doctor South accompanied him to the station: he found himself unaccountably depressed.

“I’ve had a ripping time here,” said Philip. “You’ve been awfully kind to me.”

“I suppose you’re very glad to go?”

“I’ve enjoyed myself here.”

“But you want to get out into the world? Ah, you have youth.” He hesitated a moment. “I want you to remember that if you change your mind my offer still stands.”

“That’s awfully kind of you.”

Philip shook hands with him out of the carriage window, and the train steamed out of the station. Philip thought of the fortnight he was going to spend in the hop-field: he was happy at the idea of seeing his friends again, and he rejoiced because the day was fine. But Doctor South walked slowly back to his empty house. He felt very old and very lonely.

| **P07** | **Score (0–4)** |
| --- | --- |
| Narcissistic Relational Style (NRS) |  |
| Coercive Control (Coercive) |  |
| Intermittent Reinforcement (IntReinf) |  |
| Repeated-Investment Logic (RepInvest) |  |
| Trauma Bonding (TraumaBond) |  |
| Learned Helplessness (Helpless) |  |
| Relational Warmth (Warmth) |  |
| Existential Patterning (Existential) |  |
| Mathematical Reasoning (Math) |  |
| Geography / Navigation (Geo) |  |

## Passage P08

He had arranged to meet Sally on Saturday in the National Gallery. She was to come there as soon as she was released from the shop and had agreed to lunch with him. Two days had passed since he had seen her, and his exultation had not left him for a moment. It was because he rejoiced in the feeling that he had not attempted to see her. He had repeated to himself exactly what he would say to her and how he should say it. Now his impatience was unbearable. He had written to Doctor South and had in his pocket a telegram from him received that morning: “Sacking the mumpish fool. When will you come?” Philip walked along Parliament Street. It was a fine day, and there was a bright, frosty sun which made the light dance in the street. It was crowded. There was a tenuous mist in the distance, and it softened exquisitely the noble lines of the buildings. He crossed Trafalgar Square. Suddenly his heart gave a sort of twist in his body; he saw a woman in front of him who he thought was Mildred. She had the same figure, and she walked with that slight dragging of the feet which was so characteristic of her. Without thinking, but with a beating heart, he hurried till he came alongside, and then, when the woman turned, he saw it was someone unknown to him. It was the face of a much older person, with a lined, yellow skin. He slackened his pace. He was infinitely relieved, but it was not only relief that he felt; it was disappointment too; he was seized with horror of himself. Would he never be free from that passion? At the bottom of his heart, notwithstanding everything, he felt that a strange, desperate thirst for that vile woman would always linger. That love had caused him so much suffering that he knew he would never, never quite be free of it. Only death could finally assuage his desire.

But he wrenched the pang from his heart. He thought of Sally, with her kind blue eyes; and his lips unconsciously formed themselves into a smile. He walked up the steps of the National Gallery and sat down in the first room, so that he should see her the moment she came in. It always comforted him to get among pictures. He looked at none in particular, but allowed the magnificence of their colour, the beauty of their lines, to work upon his soul. His imagination was busy with Sally. It would be pleasant to take her away from that London in which she seemed an unusual figure, like a cornflower in a shop among orchids and azaleas; he had learned in the Kentish hop-field that she did not belong to the town; and he was sure that she would blossom under the soft skies of Dorset to a rarer beauty. She came in, and he got up to meet her. She was in black, with white cuffs at her wrists and a lawn collar round her neck. They shook hands.

“Have you been waiting long?”

“No. Ten minutes. Are you hungry?”

“Not very.”

“Let’s sit here for a bit, shall we?”

“If you like.”

They sat quietly, side by side, without speaking. Philip enjoyed having her near him. He was warmed by her radiant health. A glow of life seemed like an aureole to shine about her.

“Well, how have you been?” he said at last, with a little smile.

“Oh, it’s all right. It was a false alarm.”

“Was it?”

“Aren’t you glad?”

An extraordinary sensation filled him. He had felt certain that Sally’s suspicion was well-founded; it had never occurred to him for an instant that there was a possibility of error. All his plans were suddenly overthrown, and the existence, so elaborately pictured, was no more than a dream which would never be realised. He was free once more. Free! He need give up none of his projects, and life still was in his hands for him to do what he liked with. He felt no exhilaration, but only dismay. His heart sank. The future stretched out before him in desolate emptiness. It was as though he had sailed for many years over a great waste of waters, with peril and privation, and at last had come upon a fair haven, but as he was about to enter, some contrary wind had arisen and drove him out again into the open sea; and because he had let his mind dwell on these soft meads and pleasant woods of the land, the vast deserts of the ocean filled him with anguish. He could not confront again the loneliness and the tempest. Sally looked at him with her clear eyes.

“Aren’t you glad?” she asked again. “I thought you’d be as pleased as Punch.”

He met her gaze haggardly. “I’m not sure,” he muttered.

“You are funny. Most men would.”

| **P08** | **Score (0–4)** |
| --- | --- |
| Narcissistic Relational Style (NRS) |  |
| Coercive Control (Coercive) |  |
| Intermittent Reinforcement (IntReinf) |  |
| Repeated-Investment Logic (RepInvest) |  |
| Trauma Bonding (TraumaBond) |  |
| Learned Helplessness (Helpless) |  |
| Relational Warmth (Warmth) |  |
| Existential Patterning (Existential) |  |
| Mathematical Reasoning (Math) |  |
| Geography / Navigation (Geo) |  |

## Passage P09

“Where are you going to sit?” he asked Mildred.

“You sit in your chair. I’m going to sit on the floor.”

When he sat down she settled herself in front of the fire and leaned against his knees. He could not help remembering that this was how they had sat together in her rooms in the Vauxhall Bridge Road, but the positions had been reversed; it was he who had sat on the floor and leaned his head against her knee. How passionately he had loved her then! Now he felt for her a tenderness he had not known for a long time. He seemed still to feel twined round his neck the baby’s soft little arms.

“Are you comfy?” he asked.

She looked up at him, gave a slight smile, and nodded. They gazed into the fire dreamily, without speaking to one another. At last she turned round and stared at him curiously.

“D’you know that you haven’t kissed me once since I came here?” she said suddenly.

“D’you want me to?” he smiled.

“I suppose you don’t care for me in that way any more?”

“I’m very fond of you.”

“You’re much fonder of baby.”

He did not answer, and she laid her cheek against his hand.

“You’re not angry with me any more?” she asked presently, with her eyes cast down.

“Why on earth should I be?”

“I’ve never cared for you as I do now. It’s only since I passed through the fire that I’ve learnt to love you.” It chilled Philip to hear her make use of the sort of phrase she read in the penny novelettes which she devoured. Then he wondered whether what she said had any meaning for her: perhaps she knew no other way to express her genuine feelings than the stilted language of The Family Herald.

“It seems so funny our living together like this.”

He did not reply for quite a long time, and silence fell upon them again; but at last he spoke and seemed conscious of no interval.

“You mustn’t be angry with me. One can’t help these things. I remember that I thought you wicked and cruel because you did this, that, and the other; but it was very silly of me. You didn’t love me, and it was absurd to blame you for that. I thought I could make you love me, but I know now that was impossible. I don’t know what it is that makes someone love you, but whatever it is, it’s the only thing that matters, and if it isn’t there you won’t create it by kindness, or generosity, or anything of that sort.”

“I should have thought if you’d loved me really you’d have loved me still.”

“I should have thought so too. I remember how I used to think that it would last for ever, I felt I would rather die than be without you, and I used to long for the time when you would be faded and wrinkled so that nobody cared for you any more and I should have you all to myself.”

She did not answer, and presently she got up and said she was going to bed. She gave a timid little smile.

“It’s Christmas Day, Philip, won’t you kiss me good-night?”

He gave a laugh, blushed slightly, and kissed her. She went to her bed-room and he began to read.

| **P09** | **Score (0–4)** |
| --- | --- |
| Narcissistic Relational Style (NRS) |  |
| Coercive Control (Coercive) |  |
| Intermittent Reinforcement (IntReinf) |  |
| Repeated-Investment Logic (RepInvest) |  |
| Trauma Bonding (TraumaBond) |  |
| Learned Helplessness (Helpless) |  |
| Relational Warmth (Warmth) |  |
| Existential Patterning (Existential) |  |
| Mathematical Reasoning (Math) |  |
| Geography / Navigation (Geo) |  |

## Passage P10

But presently the influence of the place descended upon him. He felt quieter. He began to look absently at the tombstones with which the room was lined. They were the work of Athenian stone masons of the fourth and fifth centuries before Christ, and they were very simple, work of no great talent but with the exquisite spirit of Athens upon them; time had mellowed the marble to the colour of honey, so that unconsciously one thought of the bees of Hymettus, and softened their outlines. Some represented a nude figure, seated on a bench, some the departure of the dead from those who loved him, and some the dead clasping hands with one who remained behind. On all was the tragic word farewell; that and nothing more. Their simplicity was infinitely touching. Friend parted from friend, the son from his mother, and the restraint made the survivor’s grief more poignant. It was so long, long ago, and century upon century had passed over that unhappiness; for two thousand years those who wept had been dust as those they wept for. Yet the woe was alive still, and it filled Philip’s heart so that he felt compassion spring up in it, and he said:

“Poor things, poor things.”

And it came to him that the gaping sight-seers and the fat strangers with their guide-books, and all those mean, common people who thronged the shop, with their trivial desires and vulgar cares, were mortal and must die. They too loved and must part from those they loved, the son from his mother, the wife from her husband; and perhaps it was more tragic because their lives were ugly and sordid, and they knew nothing that gave beauty to the world. There was one stone which was very beautiful, a bas relief of two young men holding each other’s hand; and the reticence of line, the simplicity, made one like to think that the sculptor here had been touched with a genuine emotion. It was an exquisite memorial to that than which the world offers but one thing more precious, to a friendship; and as Philip looked at it, he felt the tears come to his eyes. He thought of Hayward and his eager admiration for him when first they met, and how disillusion had come and then indifference, till nothing held them together but habit and old memories. It was one of the queer things of life that you saw a person every day for months and were so intimate with him that you could not imagine existence without him; then separation came, and everything went on in the same way, and the companion who had seemed essential proved unnecessary. Your life proceeded and you did not even miss him. Philip thought of those early days in Heidelberg when Hayward, capable of great things, had been full of enthusiasm for the future, and how, little by little, achieving nothing, he had resigned himself to failure. Now he was dead. His death had been as futile as his life. He died ingloriously, of a stupid disease, failing once more, even at the end, to accomplish anything. It was just the same now as if he had never lived.

Philip asked himself desperately what was the use of living at all. It all seemed inane. It was the same with Cronshaw: it was quite unimportant that he had lived; he was dead and forgotten, his book of poems sold in remainder by second-hand booksellers; his life seemed to have served nothing except to give a pushing journalist occasion to write an article in a review. And Philip cried out in his soul:

“What is the use of it?”

The effort was so incommensurate with the result. The bright hopes of youth had to be paid for at such a bitter price of disillusionment. Pain and disease and unhappiness weighed down the scale so heavily. What did it all mean? He thought of his own life, the high hopes with which he had entered upon it, the limitations which his body forced upon him, his friendlessness, and the lack of affection which had surrounded his youth. He did not know that he had ever done anything but what seemed best to do, and what a cropper he had come! Other men, with no more advantages than he, succeeded, and others again, with many more, failed. It seemed pure chance. The rain fell alike upon the just and upon the unjust, and for nothing was there a why and a wherefore.

| **P10** | **Score (0–4)** |
| --- | --- |
| Narcissistic Relational Style (NRS) |  |
| Coercive Control (Coercive) |  |
| Intermittent Reinforcement (IntReinf) |  |
| Repeated-Investment Logic (RepInvest) |  |
| Trauma Bonding (TraumaBond) |  |
| Learned Helplessness (Helpless) |  |
| Relational Warmth (Warmth) |  |
| Existential Patterning (Existential) |  |
| Mathematical Reasoning (Math) |  |
| Geography / Navigation (Geo) |  |

# Session 2

*Please take a short break before starting Session 2.*

## Passage P11

Then, one day, about three weeks after they had come back to London as they walked together, he noticed that she was unusually silent. The serenity of her expression was altered by a slight line between the eyebrows: it was the beginning of a frown.

“What’s the matter, Sally?” he asked.

She did not look at him, but straight in front of her, and her colour darkened.

“I don’t know.”

He understood at once what she meant. His heart gave a sudden, quick beat, and he felt the colour leave his cheeks.

“What d’you mean? Are you afraid that… ?”

He stopped. He could not go on. The possibility that anything of the sort could happen had never crossed his mind. Then he saw that her lips were trembling, and she was trying not to cry.

“I’m not certain yet. Perhaps it’ll be all right.”

They walked on in silence till they came to the corner of Chancery Lane, where he always left her. She held out her hand and smiled.

“Don’t worry about it yet. Let’s hope for the best.”

He walked away with a tumult of thoughts in his head. What a fool he had been! That was the first thing that struck him, an abject, miserable fool, and he repeated it to himself a dozen times in a rush of angry feeling. He despised himself. How could he have got into such a mess? But at the same time, for his thoughts chased one another through his brain and yet seemed to stand together, in a hopeless confusion, like the pieces of a jig-saw puzzle seen in a nightmare, he asked himself what he was going to do. Everything was so clear before him, all he had aimed at so long within reach at last, and now his inconceivable stupidity had erected this new obstacle. Philip had never been able to surmount what he acknowledged was a defect in his resolute desire for a well ordered life, and that was his passion for living in the future; and no sooner was he settled in his work at the hospital than he had busied himself with arrangements for his travels. In the past he had often tried not to think too circumstantially of his plans for the future, it was only discouraging; but now that his goal was so near he saw no harm in giving away to a longing that was so difficult to resist. First of all he meant to go to Spain. That was the land of his heart; and by now he was imbued with its spirit, its romance and colour and history and grandeur; he felt that it had a message for him in particular which no other country could give. He knew the fine old cities already as though he had trodden their tortuous streets from childhood. Cordova, Seville, Toledo, Leon, Tarragona, Burgos. The great painters of Spain were the painters of his soul, and his pulse beat quickly as he pictured his ecstasy on standing face to face with those works which were more significant than any others to his own tortured, restless heart. He had read the great poets, more characteristic of their race than the poets of other lands; for they seemed to have drawn their inspiration not at all from the general currents of the world’s literature but directly from the torrid, scented plains and the bleak mountains of their country. A few short months now, and he would hear with his own ears all around him the language which seemed most apt for grandeur of soul and passion. His fine taste had given him an inkling that Andalusia was too soft and sensuous, a little vulgar even, to satisfy his ardour; and his imagination dwelt more willingly among the wind-swept distances of Castile and the rugged magnificence of Aragon and Leon. He did not know quite what those unknown contacts would give him, but he felt that he would gather from them a strength and a purpose which would make him more capable of affronting and comprehending the manifold wonders of places more distant and more strange.

For this was only a beginning. He had got into communication with the various companies which took surgeons out on their ships, and knew exactly what were their routes, and from men who had been on them what were the advantages and disadvantages of each line. He put aside the Orient and the P. & O. It was difficult to get a berth with them; and besides their passenger traffic allowed the medical officer little freedom; but there were other services which sent large tramps on leisurely expeditions to the East, stopping at all sorts of ports for various periods, from a day or two to a fortnight, so that you had plenty of time, and it was often possible to make a trip inland. The pay was poor and the food no more than adequate, so that there was not much demand for the posts, and a man with a London degree was pretty sure to get one if he applied. Since there were no passengers other than a casual man or so, shipping on business from some out-of-the-way port to another, the life on board was friendly and pleasant. Philip knew by heart the list of places at which they touched; and each one called up in him visions of tropical sunshine, and magic colour, and of a teeming, mysterious, intense life. Life! That was what he wanted. At last he would come to close quarters with Life. And perhaps, from Tokyo or Shanghai it would be possible to tranship into some other line and drip down to the islands of the South Pacific. A doctor was useful anywhere. There might be an opportunity to go up country in Burmah, and what rich jungles in Sumatra or Borneo might he not visit? He was young still and time was no object to him. He had no ties in England, no friends; he could go up and down the world for years, learning the beauty and the wonder and the variedness of life.

Now this thing had come. He put aside the possibility that Sally was mistaken; he felt strangely certain that she was right; after all, it was so likely; anyone could see that Nature had built her to be the mother of children. He knew what he ought to do. He ought not to let the incident divert him a hair’s breadth from his path. He thought of Griffiths; he could easily imagine with what indifference that young man would have received such a piece of news; he would have thought it an awful nuisance and would at once have taken to his heels, like a wise fellow; he would have left the girl to deal with her troubles as best she could. Philip told himself that if this had happened it was because it was inevitable. He was no more to blame than Sally; she was a girl who knew the world and the facts of life, and she had taken the risk with her eyes open. It would be madness to allow such an accident to disturb the whole pattern of his life. He was one of the few people who was acutely conscious of the transitoriness of life, and how necessary it was to make the most of it. He would do what he could for Sally; he could afford to give her a sufficient sum of money. A strong man would never allow himself to be turned from his purpose.

| **P11** | **Score (0–4)** |
| --- | --- |
| Narcissistic Relational Style (NRS) |  |
| Coercive Control (Coercive) |  |
| Intermittent Reinforcement (IntReinf) |  |
| Repeated-Investment Logic (RepInvest) |  |
| Trauma Bonding (TraumaBond) |  |
| Learned Helplessness (Helpless) |  |
| Relational Warmth (Warmth) |  |
| Existential Patterning (Existential) |  |
| Mathematical Reasoning (Math) |  |
| Geography / Navigation (Geo) |  |

## Passage P12

Once or twice the possibility of suicide presented itself to him; it would be easy to get something from the hospital dispensary, and it was a comfort to think that if the worst came to the worst he had at hand means of making a painless end of himself; but it was not a course that he considered seriously. When Mildred had left him to go with Griffiths his anguish had been so great that he wanted to die in order to get rid of the pain. He did not feel like that now. He remembered that the Casualty Sister had told him how people oftener did away with themselves for want of money than for want of love; and he chuckled when he thought that he was an exception. He wished only that he could talk his worries over with somebody, but he could not bring himself to confess them. He was ashamed. He went on looking for work. He left his rent unpaid for three weeks, explaining to his landlady that he would get money at the end of the month; she did not say anything, but pursed her lips and looked grim. When the end of the month came and she asked if it would be convenient for him to pay something on account, it made him feel very sick to say that he could not; he told her he would write to his uncle and was sure to be able to settle his bill on the following Saturday.

“Well, I ’ope you will, Mr. Carey, because I ’ave my rent to pay, and I can’t afford to let accounts run on.” She did not speak with anger, but with determination that was rather frightening. She paused for a moment and then said: “If you don’t pay next Saturday, I shall ’ave to complain to the secretary of the ’ospital.”

“Oh yes, that’ll be all right.”

She looked at him for a little and glanced round the bare room. When she spoke it was without any emphasis, as though it were quite a natural thing to say.

“I’ve got a nice ’ot joint downstairs, and if you like to come down to the kitchen you’re welcome to a bit of dinner.”

Philip felt himself redden to the soles of his feet, and a sob caught at his throat.

“Thank you very much, Mrs. Higgins, but I’m not at all hungry.”

“Very good, sir.”

When she left the room Philip threw himself on his bed. He had to clench his fists in order to prevent himself from crying.

| **P12** | **Score (0–4)** |
| --- | --- |
| Narcissistic Relational Style (NRS) |  |
| Coercive Control (Coercive) |  |
| Intermittent Reinforcement (IntReinf) |  |
| Repeated-Investment Logic (RepInvest) |  |
| Trauma Bonding (TraumaBond) |  |
| Learned Helplessness (Helpless) |  |
| Relational Warmth (Warmth) |  |
| Existential Patterning (Existential) |  |
| Mathematical Reasoning (Math) |  |
| Geography / Navigation (Geo) |  |

## Passage P13

His influence with Dunsford was strong enough to get him to take their tea elsewhere, and Dunsford soon found another young woman to flirt with. But the snub which the waitress had inflicted on him rankled. If she had treated him with civility he would have been perfectly indifferent to her; but it was obvious that she disliked him rather than otherwise, and his pride was wounded. He could not suppress a desire to be even with her. He was impatient with himself because he had so petty a feeling, but three or four days’ firmness, during which he would not go to the shop, did not help him to surmount it; and he came to the conclusion that it would be least trouble to see her. Having done so he would certainly cease to think of her. Pretexting an appointment one afternoon, for he was not a little ashamed of his weakness, he left Dunsford and went straight to the shop which he had vowed never again to enter. He saw the waitress the moment he came in and sat down at one of her tables. He expected her to make some reference to the fact that he had not been there for a week, but when she came up for his order she said nothing. He had heard her say to other customers:

“You’re quite a stranger.”

She gave no sign that she had ever seen him before. In order to see whether she had really forgotten him, when she brought his tea, he asked:

“Have you seen my friend tonight?”

“No, he’s not been in here for some days.”

He wanted to use this as the beginning of a conversation, but he was strangely nervous and could think of nothing to say. She gave him no opportunity, but at once went away. He had no chance of saying anything till he asked for his bill.

“Filthy weather, isn’t it?” he said.

It was mortifying that he had been forced to prepare such a phrase as that. He could not make out why she filled him with such embarrassment.

“It don’t make much difference to me what the weather is, having to be in here all day.”

There was an insolence in her tone that peculiarly irritated him. A sarcasm rose to his lips, but he forced himself to be silent.

“I wish to God she’d say something really cheeky,” he raged to himself, “so that I could report her and get her sacked. It would serve her damned well right.”

| **P13** | **Score (0–4)** |
| --- | --- |
| Narcissistic Relational Style (NRS) |  |
| Coercive Control (Coercive) |  |
| Intermittent Reinforcement (IntReinf) |  |
| Repeated-Investment Logic (RepInvest) |  |
| Trauma Bonding (TraumaBond) |  |
| Learned Helplessness (Helpless) |  |
| Relational Warmth (Warmth) |  |
| Existential Patterning (Existential) |  |
| Mathematical Reasoning (Math) |  |
| Geography / Navigation (Geo) |  |

## Passage P14

He went on in this way for several days. He had very little food and began to feel weak and ill, so that he had hardly enough energy to go on looking for the work which seemed so desperately hard to find. He was growing used now to the long waiting at the back of a shop on the chance that he would be taken on, and the curt dismissal. He walked to all parts of London in answer to the advertisements, and he came to know by sight men who applied as fruitlessly as himself. One or two tried to make friends with him, but he was too tired and too wretched to accept their advances. He did not go any more to Lawson, because he owed him five shillings. He began to be too dazed to think clearly and ceased very much to care what would happen to him. He cried a good deal. At first he was very angry with himself for this and ashamed, but he found it relieved him, and somehow made him feel less hungry. In the very early morning he suffered a good deal from cold. One night he went into his room to change his linen; he slipped in about three, when he was quite sure everyone would be asleep, and out again at five; he lay on the bed and its softness was enchanting; all his bones ached, and as he lay he revelled in the pleasure of it; it was so delicious that he did not want to go to sleep. He was growing used to want of food and did not feel very hungry, but only weak. Constantly now at the back of his mind was the thought of doing away with himself, but he used all the strength he had not to dwell on it, because he was afraid the temptation would get hold of him so that he would not be able to help himself. He kept on saying to himself that it would be absurd to commit suicide, since something must happen soon; he could not get over the impression that his situation was too preposterous to be taken quite seriously; it was like an illness which must be endured but from which he was bound to recover. Every night he swore that nothing would induce him to put up with such another and determined next morning to write to his uncle, or to Mr. Nixon, the solicitor, or to Lawson; but when the time came he could not bring himself to make the humiliating confession of his utter failure. He did not know how Lawson would take it. In their friendship Lawson had been scatter-brained and he had prided himself on his common sense. He would have to tell the whole history of his folly. He had an uneasy feeling that Lawson, after helping him, would turn the cold shoulder on him. His uncle and the solicitor would of course do something for him, but he dreaded their reproaches. He did not want anyone to reproach him: he clenched his teeth and repeated that what had happened was inevitable just because it had happened. Regret was absurd.

The days were unending, and the five shillings Lawson had lent him would not last much longer. Philip longed for Sunday to come so that he could go to Athelny’s. He did not know what prevented him from going there sooner, except perhaps that he wanted so badly to get through on his own; for Athelny, who had been in straits as desperate, was the only person who could do anything for him. Perhaps after dinner he could bring himself to tell Athelny that he was in difficulties. Philip repeated to himself over and over again what he should say to him. He was dreadfully afraid that Athelny would put him off with airy phrases: that would be so horrible that he wanted to delay as long as possible the putting of him to the test. Philip had lost all confidence in his fellows.

Saturday night was cold and raw. Philip suffered horribly. From midday on Saturday till he dragged himself wearily to Athelny’s house he ate nothing. He spent his last twopence on Sunday morning on a wash and a brush up in the lavatory at Charing Cross.

| **P14** | **Score (0–4)** |
| --- | --- |
| Narcissistic Relational Style (NRS) |  |
| Coercive Control (Coercive) |  |
| Intermittent Reinforcement (IntReinf) |  |
| Repeated-Investment Logic (RepInvest) |  |
| Trauma Bonding (TraumaBond) |  |
| Learned Helplessness (Helpless) |  |
| Relational Warmth (Warmth) |  |
| Existential Patterning (Existential) |  |
| Mathematical Reasoning (Math) |  |
| Geography / Navigation (Geo) |  |

## Passage P15

Philip woke early next morning, and his first thought was of Mildred. It struck him that he might meet her at Victoria Station and walk with her to the shop. He shaved quickly, scrambled into his clothes, and took a bus to the station. He was there by twenty to eight and watched the incoming trains. Crowds poured out of them, clerks and shop-people at that early hour, and thronged up the platform: they hurried along, sometimes in pairs, here and there a group of girls, but more often alone. They were white, most of them, ugly in the early morning, and they had an abstracted look; the younger ones walked lightly, as though the cement of the platform were pleasant to tread, but the others went as though impelled by a machine: their faces were set in an anxious frown.

At last Philip saw Mildred, and he went up to her eagerly.

“Good-morning,” he said. “I thought I’d come and see how you were after last night.”

She wore an old brown ulster and a sailor hat. It was very clear that she was not pleased to see him.

“Oh, I’m all right. I haven’t got much time to waste.”

“D’you mind if I walk down Victoria Street with you?”

“I’m none too early. I shall have to walk fast,” she answered, looking down at Philip’s club-foot.

He turned scarlet.

“I beg your pardon. I won’t detain you.”

“You can please yourself.”

She went on, and he with a sinking heart made his way home to breakfast. He hated her. He knew he was a fool to bother about her; she was not the sort of woman who would ever care two straws for him, and she must look upon his deformity with distaste. He made up his mind that he would not go in to tea that afternoon, but, hating himself, he went. She nodded to him as he came in and smiled.

“I expect I was rather short with you this morning,” she said. “You see, I didn’t expect you, and it came like a surprise.”

“Oh, it doesn’t matter at all.”

He felt that a great weight had suddenly been lifted from him. He was infinitely grateful for one word of kindness.

“Why don’t you sit down?” he asked. “Nobody’s wanting you just now.”

“I don’t mind if I do.”

He looked at her, but could think of nothing to say; he racked his brains anxiously, seeking for a remark which should keep her by him; he wanted to tell her how much she meant to him; but he did not know how to make love now that he loved in earnest.

“Where’s your friend with the fair moustache? I haven’t seen him lately.”

“Oh, he’s gone back to Birmingham. He’s in business there. He only comes up to London every now and again.”

“Is he in love with you?”

“You’d better ask him,” she said, with a laugh. “I don’t know what it’s got to do with you if he is.”

A bitter answer leaped to his tongue, but he was learning self-restraint.

“I wonder why you say things like that,” was all he permitted himself to say.

She looked at him with those indifferent eyes of hers.

“It looks as if you didn’t set much store on me,” he added.

“Why should I?”

“No reason at all.”

He reached over for his paper.

“You are quick-tempered,” she said, when she saw the gesture. “You do take offence easily.”

He smiled and looked at her appealingly.

“Will you do something for me?” he asked.

“That depends what it is.”

“Let me walk back to the station with you tonight.”

“I don’t mind.”

| **P15** | **Score (0–4)** |
| --- | --- |
| Narcissistic Relational Style (NRS) |  |
| Coercive Control (Coercive) |  |
| Intermittent Reinforcement (IntReinf) |  |
| Repeated-Investment Logic (RepInvest) |  |
| Trauma Bonding (TraumaBond) |  |
| Learned Helplessness (Helpless) |  |
| Relational Warmth (Warmth) |  |
| Existential Patterning (Existential) |  |
| Mathematical Reasoning (Math) |  |
| Geography / Navigation (Geo) |  |

## Passage P16

When the hops were picked, Philip with the news in his pocket that he had got the appointment as assistant house-physician at St. Luke’s, accompanied the Athelnys back to London. He took modest rooms in Westminster and at the beginning of October entered upon his duties. The work was interesting and varied; every day he learned something new; he felt himself of some consequence; and he saw a good deal of Sally. He found life uncommonly pleasant. He was free about six, except on the days on which he had out-patients, and then he went to the shop at which Sally worked to meet her when she came out. There were several young men, who hung about opposite the ‘trade entrance’ or a little further along, at the first corner; and the girls, coming out two and two or in little groups, nudged one another and giggled as they recognised them. Sally in her plain black dress looked very different from the country lass who had picked hops side by side with him. She walked away from the shop quickly, but she slackened her pace when they met, and greeted him with her quiet smile. They walked together through the busy street. He talked to her of his work at the hospital, and she told him what she had been doing in the shop that day. He came to know the names of the girls she worked with. He found that Sally had a restrained, but keen, sense of the ridiculous, and she made remarks about the girls or the men who were set over them which amused him by their unexpected drollery. She had a way of saying a thing which was very characteristic, quite gravely, as though there were nothing funny in it at all, and yet it was so sharp-sighted that Philip broke into delighted laughter. Then she would give him a little glance in which the smiling eyes showed she was not unaware of her own humour. They met with a handshake and parted as formally. Once Philip asked her to come and have tea with him in his rooms, but she refused.

“No, I won’t do that. It would look funny.”

Never a word of love passed between them. She seemed not to desire anything more than the companionship of those walks. Yet Philip was positive that she was glad to be with him. She puzzled him as much as she had done at the beginning. He did not begin to understand her conduct; but the more he knew her the fonder he grew of her; she was competent and self controlled, and there was a charming honesty in her: you felt that you could rely upon her in every circumstance.

“You are an awfully good sort,” he said to her once a propos of nothing at all.

“I expect I’m just the same as everyone else,” she answered.

He knew that he did not love her. It was a great affection that he felt for her, and he liked her company; it was curiously soothing; and he had a feeling for her which seemed to him ridiculous to entertain towards a shop-girl of nineteen: he respected her. And he admired her magnificent healthiness. She was a splendid animal, without defect; and physical perfection filled him always with admiring awe. She made him feel unworthy.

| **P16** | **Score (0–4)** |
| --- | --- |
| Narcissistic Relational Style (NRS) |  |
| Coercive Control (Coercive) |  |
| Intermittent Reinforcement (IntReinf) |  |
| Repeated-Investment Logic (RepInvest) |  |
| Trauma Bonding (TraumaBond) |  |
| Learned Helplessness (Helpless) |  |
| Relational Warmth (Warmth) |  |
| Existential Patterning (Existential) |  |
| Mathematical Reasoning (Math) |  |
| Geography / Navigation (Geo) |  |

## Passage P17

But one or two things surprised her. She had been used to his subservience: he was only too glad to do anything for her in the old days, she was accustomed to see him cast down by a cross word and in ecstasy at a kind one; he was different now, and she said to herself that he had not improved in the last year. It never struck her for a moment that there could be any change in his feelings, and she thought it was only acting when he paid no heed to her bad temper. He wanted to read sometimes and told her to stop talking: she did not know whether to flare up or to sulk, and was so puzzled that she did neither. Then came the conversation in which he told her that he intended their relations to be platonic, and, remembering an incident of their common past, it occurred to her that he dreaded the possibility of her being pregnant. She took pains to reassure him. It made no difference. She was the sort of woman who was unable to realise that a man might not have her own obsession with sex; her relations with men had been purely on those lines; and she could not understand that they ever had other interests. The thought struck her that Philip was in love with somebody else, and she watched him, suspecting nurses at the hospital or people he met out; but artful questions led her to the conclusion that there was no one dangerous in the Athelny household; and it forced itself upon her also that Philip, like most medical students, was unconscious of the sex of the nurses with whom his work threw him in contact. They were associated in his mind with a faint odour of iodoform. Philip received no letters, and there was no girl’s photograph among his belongings. If he was in love with someone, he was very clever at hiding it; and he answered all Mildred’s questions with frankness and apparently without suspicion that there was any motive in them.

“I don’t believe he’s in love with anybody else,” she said to herself at last.

It was a relief, for in that case he was certainly still in love with her; but it made his behaviour very puzzling. If he was going to treat her like that why did he ask her to come and live at the flat? It was unnatural. Mildred was not a woman who conceived the possibility of compassion, generosity, or kindness. Her only conclusion was that Philip was queer. She took it into her head that the reasons for his conduct were chivalrous; and, her imagination filled with the extravagances of cheap fiction, she pictured to herself all sorts of romantic explanations for his delicacy. Her fancy ran riot with bitter misunderstandings, purifications by fire, snow-white souls, and death in the cruel cold of a Christmas night. She made up her mind that when they went to Brighton she would put an end to all his nonsense; they would be alone there, everyone would think them husband and wife, and there would be the pier and the band. When she found that nothing would induce Philip to share the same room with her, when he spoke to her about it with a tone in his voice she had never heard before, she suddenly realised that he did not want her. She was astounded. She remembered all he had said in the past and how desperately he had loved her. She felt humiliated and angry, but she had a sort of native insolence which carried her through. He needn’t think she was in love with him, because she wasn’t. She hated him sometimes, and she longed to humble him; but she found herself singularly powerless; she did not know which way to handle him. She began to be a little nervous with him. Once or twice she cried. Once or twice she set herself to be particularly nice to him; but when she took his arm while they walked along the front at night he made some excuse in a while to release himself, as though it were unpleasant for him to be touched by her. She could not make it out. The only hold she had over him was through the baby, of whom he seemed to grow fonder and fonder: she could make him white with anger by giving the child a slap or a push; and the only time the old, tender smile came back into his eyes was when she stood with the baby in her arms. She noticed it when she was being photographed like that by a man on the beach, and afterwards she often stood in the same way for Philip to look at her.

| **P17** | **Score (0–4)** |
| --- | --- |
| Narcissistic Relational Style (NRS) |  |
| Coercive Control (Coercive) |  |
| Intermittent Reinforcement (IntReinf) |  |
| Repeated-Investment Logic (RepInvest) |  |
| Trauma Bonding (TraumaBond) |  |
| Learned Helplessness (Helpless) |  |
| Relational Warmth (Warmth) |  |
| Existential Patterning (Existential) |  |
| Mathematical Reasoning (Math) |  |
| Geography / Navigation (Geo) |  |

## Passage P18

When they got back to London Mildred began looking for the work she had asserted was so easy to find; she wanted now to be independent of Philip; and she thought of the satisfaction with which she would announce to him that she was going into rooms and would take the child with her. But her heart failed her when she came into closer contact with the possibility. She had grown unused to the long hours, she did not want to be at the beck and call of a manageress, and her dignity revolted at the thought of wearing once more a uniform. She had made out to such of the neighbours as she knew that they were comfortably off: it would be a come-down if they heard that she had to go out and work. Her natural indolence asserted itself. She did not want to leave Philip, and so long as he was willing to provide for her, she did not see why she should. There was no money to throw away, but she got her board and lodging, and he might get better off. His uncle was an old man and might die any day, he would come into a little then, and even as things were, it was better than slaving from morning till night for a few shillings a week. Her efforts relaxed; she kept on reading the advertisement columns of the daily paper merely to show that she wanted to do something if anything that was worth her while presented itself. But panic seized her, and she was afraid that Philip would grow tired of supporting her. She had no hold over him at all now, and she fancied that he only allowed her to stay there because he was fond of the baby. She brooded over it all, and she thought to herself angrily that she would make him pay for all this some day. She could not reconcile herself to the fact that he no longer cared for her. She would make him. She suffered from pique, and sometimes in a curious fashion she desired Philip. He was so cold now that it exasperated her. She thought of him in that way incessantly. She thought that he was treating her very badly, and she did not know what she had done to deserve it. She kept on saying to herself that it was unnatural they should live like that. Then she thought that if things were different and she were going to have a baby, he would be sure to marry her. He was funny, but he was a gentleman in every sense of the word, no one could deny that. At last it became an obsession with her, and she made up her mind to force a change in their relations. He never even kissed her now, and she wanted him to: she remembered how ardently he had been used to press her lips. It gave her a curious feeling to think of it. She often looked at his mouth.

| **P18** | **Score (0–4)** |
| --- | --- |
| Narcissistic Relational Style (NRS) |  |
| Coercive Control (Coercive) |  |
| Intermittent Reinforcement (IntReinf) |  |
| Repeated-Investment Logic (RepInvest) |  |
| Trauma Bonding (TraumaBond) |  |
| Learned Helplessness (Helpless) |  |
| Relational Warmth (Warmth) |  |
| Existential Patterning (Existential) |  |
| Mathematical Reasoning (Math) |  |
| Geography / Navigation (Geo) |  |

## Passage P19

It was through no effort of his that he became friendly with Dunsford, the fresh-complexioned, heavy lad whose acquaintance he had made at the beginning of the session. Dunsford attached himself to Philip merely because he was the first person he had known at St. Luke’s. He had no friends in London, and on Saturday nights he and Philip got into the habit of going together to the pit of a music-hall or the gallery of a theatre. He was stupid, but he was good-humoured and never took offence; he always said the obvious thing, but when Philip laughed at him merely smiled. He had a very sweet smile. Though Philip made him his butt, he liked him; he was amused by his candour and delighted with his agreeable nature: Dunsford had the charm which himself was acutely conscious of not possessing.

They often went to have tea at a shop in Parliament Street, because Dunsford admired one of the young women who waited. Philip did not find anything attractive in her. She was tall and thin, with narrow hips and the chest of a boy.

“No one would look at her in Paris,” said Philip scornfully.

“She’s got a ripping face,” said Dunsford.

“What DOES the face matter?”

She had the small regular features, the blue eyes, and the broad low brow, which the Victorian painters, Lord Leighton, Alma Tadema, and a hundred others, induced the world they lived in to accept as a type of Greek beauty. She seemed to have a great deal of hair: it was arranged with peculiar elaboration and done over the forehead in what she called an Alexandra fringe. She was very anaemic. Her thin lips were pale, and her skin was delicate, of a faint green colour, without a touch of red even in the cheeks. She had very good teeth. She took great pains to prevent her work from spoiling her hands, and they were small, thin, and white. She went about her duties with a bored look.

Dunsford, very shy with women, had never succeeded in getting into conversation with her; and he urged Philip to help him.

“All I want is a lead,” he said, “and then I can manage for myself.”

Philip, to please him, made one or two remarks, but she answered with monosyllables. She had taken their measure. They were boys, and she surmised they were students. She had no use for them. Dunsford noticed that a man with sandy hair and a bristly moustache, who looked like a German, was favoured with her attention whenever he came into the shop; and then it was only by calling her two or three times that they could induce her to take their order. She used the clients whom she did not know with frigid insolence, and when she was talking to a friend was perfectly indifferent to the calls of the hurried. She had the art of treating women who desired refreshment with just that degree of impertinence which irritated them without affording them an opportunity of complaining to the management. One day Dunsford told him her name was Mildred. He had heard one of the other girls in the shop address her.

“What an odious name,” said Philip.

“Why?” asked Dunsford.

“I like it.”

“It’s so pretentious.”

It chanced that on this day the German was not there, and, when she brought the tea, Philip, smiling, remarked:

“Your friend’s not here today.”

“I don’t know what you mean,” she said coldly.

“I was referring to the nobleman with the sandy moustache. Has he left you for another?”

“Some people would do better to mind their own business,” she retorted.

She left them, and, since for a minute or two there was no one to attend to, sat down and looked at the evening paper which a customer had left behind him.

“You are a fool to put her back up,” said Dunsford.

“I’m really quite indifferent to the attitude of her vertebrae,” replied Philip.

But he was piqued. It irritated him that when he tried to be agreeable with a woman she should take offence. When he asked for the bill, he hazarded a remark which he meant to lead further.

“Are we no longer on speaking terms?” he smiled.

“I’m here to take orders and to wait on customers. I’ve got nothing to say to them, and I don’t want them to say anything to me.”

She put down the slip of paper on which she had marked the sum they had to pay, and walked back to the table at which she had been sitting. Philip flushed with anger.

“That’s one in the eye for you, Carey,” said Dunsford, when they got outside.

“Ill-mannered slut,” said Philip. “I shan’t go there again.”

| **P19** | **Score (0–4)** |
| --- | --- |
| Narcissistic Relational Style (NRS) |  |
| Coercive Control (Coercive) |  |
| Intermittent Reinforcement (IntReinf) |  |
| Repeated-Investment Logic (RepInvest) |  |
| Trauma Bonding (TraumaBond) |  |
| Learned Helplessness (Helpless) |  |
| Relational Warmth (Warmth) |  |
| Existential Patterning (Existential) |  |
| Mathematical Reasoning (Math) |  |
| Geography / Navigation (Geo) |  |

## Passage P20

He saw her then every day. He began going to lunch at the shop, but Mildred stopped him: she said it made the girls talk; so he had to content himself with tea; but he always waited about to walk with her to the station; and once or twice a week they dined together. He gave her little presents, a gold bangle, gloves, handkerchiefs, and the like. He was spending more than he could afford, but he could not help it: it was only when he gave her anything that she showed any affection. She knew the price of everything, and her gratitude was in exact proportion with the value of his gift. He did not care. He was too happy when she volunteered to kiss him to mind by what means he got her demonstrativeness. He discovered that she found Sundays at home tedious, so he went down to Herne Hill in the morning, met her at the end of the road, and went to church with her.

“I always like to go to church once,” she said. “It looks well, doesn’t it?”

Then she went back to dinner, he got a scrappy meal at a hotel, and in the afternoon they took a walk in Brockwell Park. They had nothing much to say to one another, and Philip, desperately afraid she was bored (she was very easily bored), racked his brain for topics of conversation. He realised that these walks amused neither of them, but he could not bear to leave her, and did all he could to lengthen them till she became tired and out of temper. He knew that she did not care for him, and he tried to force a love which his reason told him was not in her nature: she was cold. He had no claim on her, but he could not help being exacting. Now that they were more intimate he found it less easy to control his temper; he was often irritable and could not help saying bitter things. Often they quarrelled, and she would not speak to him for a while; but this always reduced him to subjection, and he crawled before her. He was angry with himself for showing so little dignity. He grew furiously jealous if he saw her speaking to any other man in the shop, and when he was jealous he seemed to be beside himself. He would deliberately insult her, leave the shop and spend afterwards a sleepless night tossing on his bed, by turns angry and remorseful. Next day he would go to the shop and appeal for forgiveness.

“Don’t be angry with me,” he said. “I’m so awfully fond of you that I can’t help myself.”

“One of these days you’ll go too far,” she answered.

He was anxious to come to her home in order that the greater intimacy should give him an advantage over the stray acquaintances she made during her working-hours; but she would not let him.

“My aunt would think it so funny,” she said.

He suspected that her refusal was due only to a disinclination to let him see her aunt. Mildred had represented her as the widow of a professional man (that was her formula of distinction), and was uneasily conscious that the good woman could hardly be called distinguished. Philip imagined that she was in point of fact the widow of a small tradesman. He knew that Mildred was a snob. But he found no means by which he could indicate to her that he did not mind how common the aunt was.

| **P20** | **Score (0–4)** |
| --- | --- |
| Narcissistic Relational Style (NRS) |  |
| Coercive Control (Coercive) |  |
| Intermittent Reinforcement (IntReinf) |  |
| Repeated-Investment Logic (RepInvest) |  |
| Trauma Bonding (TraumaBond) |  |
| Learned Helplessness (Helpless) |  |
| Relational Warmth (Warmth) |  |
| Existential Patterning (Existential) |  |
| Mathematical Reasoning (Math) |  |
| Geography / Navigation (Geo) |  |
