# Supplementary material for: Mapping relational mechanism clusters in Of Human Bondage: a theory-driven multiscale embedding analysis
Source: Front Psychol. 2026 Jul 16;17:1836153. doi: 10.3389/fpsyg.2026.1836153 (PMC13420877; doi:10.3389/fpsyg.2026.1836153)
Supplement: Supplementary file 3 [file Table_1.DOCX]

Supplementary Appendix S1

# Semantic Anchor Specification: Full Variant Texts and Provenance

This appendix lists the full text of every semantic anchor used in the study. Each construct is represented by four variants (V0–V3); the construct-level anchor embedding is the mean of the four unit-normalized variant embeddings (see Methods §2.5). V0 is the original reference text; V1–V3 are paraphrase variants written to stabilize the construct representation across stylistic differences. All anchor texts were finalized before the analyses reported in this manuscript and were not altered in response to model outputs. The SHA-256 hashes of the source files are provided below to establish provenance.

## Provenance (SHA-256 of source files)

| File | SHA-256 |
| --- | --- |
| anchor_variants.json | 5b2f59bb317e488ae7350239b9bf72d1945324aced85ba20c7f5a29c12daa3d6 |
| negative_controls.json | fd900c88d16a494848e30680d07b113a05ad29508e2b4a6908026631c00d94be |

## Internal key — manuscript label map

| Internal key (preserved in code and JSON) | Manuscript label | Control type |
| --- | --- | --- |
| REF_NPD_BEHAVIOR | Narcissistic Relational Style (NRS) | Positive |
| REF_COERCIVE_CONTROL | Coercive Control | Positive |
| REF_INTERMITTENT_REINFORCEMENT | Intermittent Reinforcement | Positive |
| REF_GAMBLERS_FALLACY | Repeated-Investment Logic | Positive |
| REF_TRAUMA_BONDING | Trauma Bonding | Positive |
| REF_LEARNED_HELPLESSNESS | Learned Helplessness | Positive |
| REF_HEALTHY_LOVE | Relational Warmth | Positive |
| REF_CRONSHAWS_PHILOSOPHY | Existential Patterning | Positive |
| REF_MATHEMATICAL_REASONING | Math Reasoning [NEG] | Negative |
| REF_GEOGRAPHY_NAVIGATION | Geography/Navigation [NEG] | Negative |

*Label changes between the internal keys and the manuscript labels are display-string changes only; the anchor texts themselves (V0–V3 below) were not modified at any point, and the SHA-256 hashes above confirm that the file used at encoding time is byte-identical to the file released here.*

## Positive-control anchors (V0–V3)

### Narcissistic Relational Style (NRS) — internal key: REF_NPD_BEHAVIOR

| Variant | Text |
| --- | --- |
| V0 | A pervasive pattern of grandiosity, need for admiration, and lack of empathy. Interpersonally exploitative, taking advantage of others for their own ends. Sense of entitlement with unreasonable expectations of favorable treatment. Unwilling to recognize or identify with the feelings and needs of others. Shows arrogant, haughty behaviors and attitudes. |
| V1 | The person maintains an inflated self-image and expects special treatment. They seek admiration and become resentful when it is not provided. They use others instrumentally, focusing on their own needs while minimizing others’ feelings. Criticism triggers defensiveness, contempt, or blame-shifting. Their interactions often communicate superiority and entitlement. |
| V2 | They treat relationships as a stage for status and validation. Others are valued mainly for usefulness, attention, or compliance. Empathy is limited: the person discounts hurt they cause and reframes conflicts as others’ failures. They demand loyalty and praise, and respond to boundaries with irritation, ridicule, or punishment. |
| V3 | A stable interpersonal style marked by self-importance, external validation seeking, and reduced concern for others’ inner states. The individual expects preferential outcomes, exploits asymmetries to their advantage, and shows arrogance or disdain. They interpret disagreement as disrespect and maintain control through devaluation, blame, or strategic charm. |

### Coercive Control — internal key: REF_COERCIVE_CONTROL

| Variant | Text |
| --- | --- |
| V0 | A strategic, ongoing pattern of domination that restricts a victim's autonomy and liberty. The abuser uses isolation, surveillance, microregulation of daily behavior, economic exploitation, and threats to maintain power. The victim is systematically deprived of independence and subjected to rules and punishments. Control is exercised through intimidation, degradation, and the creation of fear and dependency. |
| V1 | A sustained pattern of domination that limits another person’s freedom. The controlling partner sets rules, monitors behavior, and punishes deviations through threats, humiliation, or withdrawal. Over time, the victim’s choices shrink and independence erodes. The environment becomes organized around compliance, fear, and dependency. |
| V2 | Control is maintained through everyday restrictions rather than isolated assaults. The abuser dictates routines, finances, contacts, and acceptable behavior, using intimidation and degradation to enforce obedience. The victim is made to feel unsafe, watched, or constantly at fault, and learns to self-censor to avoid consequences. |
| V3 | An ongoing interpersonal regime aimed at restricting autonomy and producing compliance. Tactics include isolation, economic constraint, microregulation, punishment, and implied threat. The target adapts by anticipating the controller’s reactions, narrowing actions to what is permitted, and becoming dependent on the controller’s approval or tolerance. |

### Intermittent Reinforcement — internal key: REF_INTERMITTENT_REINFORCEMENT

| Variant | Text |
| --- | --- |
| V0 | Rewards are delivered unpredictably and inconsistently. The subject never knows when the next positive response will occur. This creates an addictive cycle where the rare reward produces an intense dopamine spike. The subject becomes obsessively focused on obtaining the next reward. Periods of rejection are followed by brief moments of warmth, creating a powerful emotional dependency. |
| V1 | Positive responses arrive on an irregular schedule. Because rewards are rare and unpredictable, the person remains vigilant for signs of approval. Small moments of kindness feel disproportionately powerful after long stretches of rejection. The uncertainty keeps attention locked onto the relationship, strengthening pursuit and making disengagement difficult. |
| V2 | The pattern alternates between cold withdrawal and brief warmth without warning. This inconsistency creates heightened hope and intense relief when the positive moment appears. The person becomes preoccupied with earning the next good moment and may tolerate repeated disappointments because the occasional reward feels like proof that improvement is possible. |
| V3 | Inconsistent reinforcement sustains persistence: rewards are delivered unpredictably, so the subject cannot learn when to stop trying. Scarce positive feedback increases salience, and variability maintains engagement. Rejection and acceptance are interwoven, producing a cycle of anticipation, renewed effort, and renewed dependence on the next favorable signal. |

### Repeated-Investment Logic — internal key: REF_GAMBLERS_FALLACY

| Variant | Text |
| --- | --- |
| V0 | After repeated losses, the person believes they are due for a win. They continue investing time, money, and emotional energy despite consistent failure, convinced that the next attempt will finally yield success. The mistaken belief that past failures increase the probability of future success. |
| V1 | After many failures, the person assumes success must be approaching. They treat continued effort as an investment that will soon pay off, even when evidence suggests otherwise. Past losses are seen as making a future win more likely. This belief sustains persistence and deepens commitment despite repeated disappointment. |
| V2 | They reason as if chance has a memory: “it has gone badly for so long that it should turn around.” In relationships, they keep pouring time, emotion, and sacrifice into an unresponsive situation, convinced the next attempt will finally change things. The longer they invest, the harder it becomes to stop. |
| V3 | A biased expectation that prior losses increase the likelihood of future gains. The individual escalates commitment after repeated setbacks, interpreting continued failure as evidence that a breakthrough is ‘due.’ This mistaken logic maintains effort and attachment even when outcomes remain consistently negative. |

### Trauma Bonding — internal key: REF_TRAUMA_BONDING

| Variant | Text |
| --- | --- |
| V0 | An emotional bond between an abuser and victim through cyclical abuse and intermittent positive reinforcement. The victim develops strong attachment despite mistreatment. Power imbalances and periods of kindness create cognitive dissonance. The victim rationalizes the abuser's behavior and blames themselves. |
| V1 | A strong attachment forms in a relationship where harm and occasional care are intertwined. The injured partner becomes emotionally invested despite mistreatment. Power imbalance and sporadic kindness produce confusion and self-doubt. To reduce inner conflict, the person explains away the other’s behavior and often turns blame inward. |
| V2 | In an unequal relationship, repeated episodes of cruelty are punctuated by brief tenderness. The contrast intensifies longing and makes the bond feel urgent. The person experiences cognitive dissonance—holding both “this is harmful” and “this is love.” They cope by rationalizing the harm, focusing on rare positives, and hoping for change. |
| V3 | An attachment maintained under cycles of threat and intermittent relief. The dependent partner remains connected through fear, hope, and the memory of occasional kindness. Imbalance in power and uncertainty promote self-blame, normalization of harm, and increased psychological commitment to the relationship despite ongoing injury. |

### Learned Helplessness — internal key: REF_LEARNED_HELPLESSNESS

| Variant | Text |
| --- | --- |
| V0 | The individual repeatedly experiences uncontrollable negative outcomes. Over time, they develop a belief that nothing they do will change the situation. They stop trying to escape or improve their circumstances. They experience cognitive deficits, motivational passivity, and emotional depression. They feel powerless, resign themselves to suffering, and accept the painful situation as inevitable. |
| V1 | A person faces repeated setbacks that they cannot influence or prevent. With time, they come to expect failure regardless of effort. They reduce attempts to change the situation and withdraw from problem-solving. Motivation declines, thinking becomes narrowed, and mood turns persistently low. They settle into resignation and endure the situation rather than trying to alter it. |
| V2 | After many experiences where action makes no difference, the person begins to treat effort as pointless. They hesitate to try, delay decisions, and accept negative outcomes in advance. Even when opportunities appear, they respond with passivity or avoidance. Their sense of agency erodes, and they adapt to suffering as if it were fixed and unavoidable. |
| V3 | Repeated exposure to uncontrollable adversity leads to learned expectations of ineffectiveness. The individual disengages from goal-directed behavior and shows reduced initiative. They display diminished persistence, lowered confidence in action-outcome links, and a subdued emotional tone. Over time, they accept hardship as the default and cease attempts to improve circumstances. |

### Relational Warmth — internal key: REF_HEALTHY_LOVE

| Variant | Text |
| --- | --- |
| V0 | Mutual respect, trust, and emotional reciprocity characterize the relationship. Both partners feel safe and valued. Communication is open and honest. Each person maintains their sense of self while sharing intimacy. The relationship brings joy, security, and personal growth. |
| V1 | The relationship is characterized by mutual care, reliability, and respect. Both people feel emotionally safe and able to express needs without fear of retaliation. Conflict is addressed with listening and repair rather than punishment. Intimacy grows alongside autonomy, and the bond supports wellbeing and development. |
| V2 | Partners communicate directly and treat each other as equals. Trust is built through consistent behavior, honesty, and accountability. Each person’s boundaries are recognized, and affection is not used as leverage. The relationship feels steady, supportive, and life-enhancing rather than destabilizing or fear-driven. |
| V3 | A reciprocal bond featuring warmth, stability, and secure connection. The partners value each other’s agency, sustain openness in communication, and respond to stress with cooperation. Closeness coexists with independence, and the relationship promotes confidence, calm, and constructive growth. |

### Existential Patterning — internal key: REF_CRONSHAWS_PHILOSOPHY

| Variant | Text |
| --- | --- |
| V0 | The meaning of life is like a pattern in a Persian carpet. You can create any design you wish. Life has no inherent meaning; it is up to each individual to weave their own pattern. The wise person recognizes the futility of passion and desire. One should observe life with detachment and find beauty in the pattern itself. |
| V1 | The answer to the riddle of existence can be found by observing ancient woven artifacts in a museum. Life is a profound mystery that each person must attempt to solve. There is a hidden truth in the intricate arrangements of color and threads, suggesting that human existence might be understood through aesthetic contemplation rather than following strict moral rules. |
| V2 | Philosophical truth cannot be simply handed down from a teacher; it must be discovered through personal experience. When confronted with the mystery of existence, an individual must seek their own answers. The ultimate secret of life is waiting to be deciphered, but the realization must come from within after a long period of enduring confusion and suffering. |
| V3 | Human existence is fundamentally devoid of objective purpose or predefined teleology. Instead, individuals are free agents who construct personal meaning through the careful arrangement of their life experiences into an aesthetic whole. By adopting a stance of philosophical detachment and relinquishing the pursuit of fleeting emotional desires, one can achieve inner tranquility and self-justification. |

## Negative-control anchors (V0–V3)

### Math Reasoning [NEG] — internal key: REF_MATHEMATICAL_REASONING

| Variant | Text |
| --- | --- |
| V0 | A person approaches a formal problem by identifying the premises, defining the relevant quantities, and applying explicit rules of deduction. Each step must follow from the previous one, and intermediate results are checked for internal consistency before the conclusion is accepted. The task depends on ordered reasoning, exact symbolic manipulation, and the demonstrable validity of the final solution. |
| V1 | The activity involves working through equations, relations, and constraints in a systematic sequence. Expressions are transformed according to established principles until the unknown value or general rule is derived. The emphasis falls on precision, coherence, and proof rather than on personal interpretation or situational judgment. |
| V2 | A thinker examines a structured set of propositions and determines whether the conclusion follows from the assumptions. Invalid inferences are rejected, alternative derivations are compared, and the argument is completed only when each transition can be justified. Success depends on disciplined logic, explicit method, and the reliable organization of abstract information. |
| V3 | The task centers on calculation, formal proof, and the stepwise resolution of a defined problem. Symbols and quantities are handled under general rules, and each intermediate operation is evaluated for correctness before proceeding. What matters is exact reasoning, stable inferential structure, and the transparent derivation of a result. |

### Geography/Navigation [NEG] — internal key: REF_GEOGRAPHY_NAVIGATION

| Variant | Text |
| --- | --- |
| V0 | A traveler determines direction and position by consulting landmarks, distances, and the relation between one point and another. Roads, boundaries, coastlines, and changes in elevation are used to construct an accurate route through unfamiliar terrain. The task requires spatial orientation, route planning, and continual adjustment to the physical layout of the environment. |
| V1 | The activity involves reading maps, tracing routes, and estimating location from known reference points. Rivers, roads, settlements, and topographic features provide the information needed to decide where one is and which path should be taken next. The emphasis is on spatial arrangement, direction, and the practical organization of movement through geographic space. |
| V2 | A navigator moves through an unfamiliar region by comparing the surrounding landscape with a map or a written set of directions. Progress depends on recognizing landmarks, judging distance, and correcting course when the observed terrain no longer matches the expected route. The process is defined by orientation, path selection, and the accurate interpretation of geographic features. |
| V3 | Someone plans a journey by identifying destinations, intermediate stops, and the most efficient route between them. Position is established through coordinates, boundaries, and recognizable features on the ground, while movement is guided by direction, distance, and terrain. The central concerns are navigation, spatial reference, and the orderly passage from one location to another. |
