# Supplementary material for: Mapping relational mechanism clusters in Of Human Bondage: a theory-driven multiscale embedding analysis
Source: Front Psychol. 2026 Jul 16;17:1836153. doi: 10.3389/fpsyg.2026.1836153 (PMC13420877; doi:10.3389/fpsyg.2026.1836153)
Supplement: Supplementary file 4 [file Table_2.DOCX]

Supplementary Appendix S2

# Corpus Inventory: Focal-Arc Chapter Membership and 165-Scene Listing

## Part A. Focal-arc chapter table (Chapters 55-122)

For every focal chapter (Chs. 55-122), this table lists the macro-group and arc/cluster labels used to define the focal subset and the Test 2 contrast. The "Test 2" column indicates whether the chapter is included in the Mildred Arc vs. Resolution comparison (Section 3.2): chapters in either Mildred Arc or Resolution are included; chapters in any other macro-group are focal but excluded from this specific contrast by design. Group totals: Mildred Arc = 40 (Chs. 55-65, 69-80, 90-105, 109); Resolution = 13 (Chs. 110-122); other focal = 15 (Norah Contrast 66-68, Contextual Prelude 81-89, Cronshaw Anchor 106, Transition 107-108).

| Chapter | Macro group | Arc label (phase) | Cluster label | Test 2 |
| --- | --- | --- | --- | --- |
| 55 | Mildred Arc | Phase 1: First Encounter | First Encounter | Yes |
| 56 | Mildred Arc | Phase 1: First Encounter | First Encounter | Yes |
| 57 | Mildred Arc | Phase 1: First Encounter | First Encounter | Yes |
| 58 | Mildred Arc | Phase 1: First Encounter | First Encounter | Yes |
| 59 | Mildred Arc | Phase 2: First Separation | Separation/Rejection | Yes |
| 60 | Mildred Arc | Phase 2: First Separation | Separation/Rejection | Yes |
| 61 | Mildred Arc | Phase 2: First Separation | Separation/Rejection | Yes |
| 62 | Mildred Arc | Phase 3a: First Reunion (Mildred) | Reunion/Return | Yes |
| 63 | Mildred Arc | Phase 3a: First Reunion (Mildred) | Reunion/Return | Yes |
| 64 | Mildred Arc | Phase 3a: First Reunion (Mildred) | Reunion/Return | Yes |
| 65 | Mildred Arc | Phase 3a: First Reunion (Mildred) | Reunion/Return | Yes |
| 66 | Norah Contrast | Phase 3b: Norah Interlude | Alternative Attachment | No |
| 67 | Norah Contrast | Phase 3b: Norah Interlude | Alternative Attachment | No |
| 68 | Norah Contrast | Phase 3b: Norah Interlude | Alternative Attachment | No |
| 69 | Mildred Arc | Phase 4: Mildred Returns (Norah Abandoned) | Return / Abandonment | Yes |
| 70 | Mildred Arc | Phase 4: Mildred Returns (Norah Abandoned) | Return / Abandonment | Yes |
| 71 | Mildred Arc | Phase 4: Mildred Returns (Norah Abandoned) | Return / Abandonment | Yes |
| 72 | Mildred Arc | Phase 5: Second Reunion | Reunion/Return | Yes |
| 73 | Mildred Arc | Phase 5: Second Reunion | Reunion/Return | Yes |
| 74 | Mildred Arc | Phase 5: Second Reunion | Reunion/Return | Yes |
| 75 | Mildred Arc | Phase 5: Second Reunion | Reunion/Return | Yes |
| 76 | Mildred Arc | Phase 5: Second Reunion | Reunion/Return | Yes |
| 77 | Mildred Arc | Phase 5: Second Reunion | Reunion/Return | Yes |
| 78 | Mildred Arc | Phase 6: Griffiths Betrayal | Separation/Rejection | Yes |
| 79 | Mildred Arc | Phase 6: Griffiths Betrayal | Separation/Rejection | Yes |
| 80 | Mildred Arc | Phase 6: Griffiths Betrayal | Separation/Rejection | Yes |
| 81 | Contextual Prelude | Phase 7: Bondage Prelude (Cronshaw / Athelny / Sally groundwork) | Contextual Prelude | No |
| 82 | Contextual Prelude | Phase 7: Bondage Prelude (Cronshaw / Athelny / Sally groundwork) | Contextual Prelude | No |
| 83 | Contextual Prelude | Phase 7: Bondage Prelude (Cronshaw / Athelny / Sally groundwork) | Contextual Prelude | No |
| 84 | Contextual Prelude | Phase 7: Bondage Prelude (Cronshaw / Athelny / Sally groundwork) | Contextual Prelude | No |
| 85 | Contextual Prelude | Phase 7: Bondage Prelude (Cronshaw / Athelny / Sally groundwork) | Contextual Prelude | No |
| 86 | Contextual Prelude | Phase 7: Bondage Prelude (Cronshaw / Athelny / Sally groundwork) | Contextual Prelude | No |
| 87 | Contextual Prelude | Phase 7: Bondage Prelude (Cronshaw / Athelny / Sally groundwork) | Contextual Prelude | No |
| 88 | Contextual Prelude | Phase 7: Bondage Prelude (Cronshaw / Athelny / Sally groundwork) | Contextual Prelude | No |
| 89 | Contextual Prelude | Phase 7: Bondage Prelude (Cronshaw / Athelny / Sally groundwork) | Contextual Prelude | No |
| 90 | Mildred Arc | Phase 8: Cohabitation | Marriage/Cohabitation | Yes |
| 91 | Mildred Arc | Phase 8: Cohabitation | Marriage/Cohabitation | Yes |
| 92 | Mildred Arc | Phase 8: Cohabitation | Marriage/Cohabitation | Yes |
| 93 | Mildred Arc | Phase 8: Cohabitation | Marriage/Cohabitation | Yes |
| 94 | Mildred Arc | Phase 8: Cohabitation | Marriage/Cohabitation | Yes |
| 95 | Mildred Arc | Phase 8: Cohabitation | Marriage/Cohabitation | Yes |
| 96 | Mildred Arc | Phase 8: Cohabitation | Marriage/Cohabitation | Yes |
| 97 | Mildred Arc | Phase 8: Cohabitation | Marriage/Cohabitation | Yes |
| 98 | Mildred Arc | Phase 9: Financial Collapse | Collapse / Poverty | Yes |
| 99 | Mildred Arc | Phase 9: Financial Collapse | Collapse / Poverty | Yes |
| 100 | Mildred Arc | Phase 9: Financial Collapse | Collapse / Poverty | Yes |
| 101 | Mildred Arc | Phase 9: Financial Collapse | Collapse / Poverty | Yes |
| 102 | Mildred Arc | Phase 10: Aftermath | Aftermath/Breakdown | Yes |
| 103 | Mildred Arc | Phase 10: Aftermath | Aftermath/Breakdown | Yes |
| 104 | Mildred Arc | Phase 10: Aftermath | Aftermath/Breakdown | Yes |
| 105 | Mildred Arc | Phase 10: Aftermath | Aftermath/Breakdown | Yes |
| 106 | Cronshaw Anchor | Phase 11: Cronshaw's Revelation | Meaning Reorientation | No |
| 107 | Transition | Phase 12: Transition / Reorientation | Transition | No |
| 108 | Transition | Phase 12: Transition / Reorientation | Transition | No |
| 109 | Mildred Arc | Phase 13: Last Mildred | Aftermath/Breakdown | Yes |
| 110 | Resolution | Phase 14: Sally, Work & Reconstruction | Reconstruction | Yes |
| 111 | Resolution | Phase 14: Sally, Work & Reconstruction | Reconstruction | Yes |
| 112 | Resolution | Phase 14: Sally, Work & Reconstruction | Reconstruction | Yes |
| 113 | Resolution | Phase 14: Sally, Work & Reconstruction | Reconstruction | Yes |
| 114 | Resolution | Phase 14: Sally, Work & Reconstruction | Reconstruction | Yes |
| 115 | Resolution | Phase 14: Sally, Work & Reconstruction | Reconstruction | Yes |
| 116 | Resolution | Phase 14: Sally, Work & Reconstruction | Reconstruction | Yes |
| 117 | Resolution | Phase 14: Sally, Work & Reconstruction | Reconstruction | Yes |
| 118 | Resolution | Phase 15: Final Integration / Residual Bondage | Final Integration | Yes |
| 119 | Resolution | Phase 15: Final Integration / Residual Bondage | Final Integration | Yes |
| 120 | Resolution | Phase 15: Final Integration / Residual Bondage | Final Integration | Yes |
| 121 | Resolution | Phase 15: Final Integration / Residual Bondage | Final Integration | Yes |
| 122 | Resolution | Phase 15: Final Integration / Residual Bondage | Final Integration | Yes |

## Part B. 165-Scene Inventory with segmentation boundaries (Chapters 55-122)

For each of the 165 hand-segmented scenes, this table reports the scene identifier, chapter and within-chapter scene index, arc and cluster labels, the focal label used in Section 3.5, the word count, and the scene-boundary locator (the first words ... the last words of the scene). The boundary locator lets any reader verify each segmentation point directly against the public-domain text. Scene boundaries were drawn by narrative-functional shifts rather than by sentence counts or fixed token windows (Section 2.2).

| Scene ID | Ch | Sc | Arc label | Cluster label | Focal label | Words | Scene boundary (first words … last words) |
| --- | --- | --- | --- | --- | --- | --- | --- |
| ch055_s01 | 55 | 1 | Medical Training and the Initial Mildred Sequence | Medical Isolation and Initial Vulnerability | Medical-School Background | 1008 | Philip’s ideas of the life of medical students... … possible for him to live a good deal by himself. |
| ch055_s02 | 55 | 2 | Medical Training and the Initial Mildred Sequence | Initial Mildred Courtship | Tea-Shop Introduction and First Humiliation | 798 | It was through no effort of his that he became … “Ill-mannered slut,” said Philip. “I shan’t go there again.” |
| ch055_s03 | 55 | 3 | Medical Training and the Initial Mildred Sequence | Initial Mildred Courtship | Post-Snub Rumination | 423 | His influence with Dunsford was strong enough... … get her sacked. It would serve her damned well right.” |
| ch056_s01 | 56 | 1 | Medical Training and the Initial Mildred Sequence | Initial Mildred Courtship | Repeated Return and First Softening | 925 | He could not get her out of his mind... … “She’s not a bad sort,” he murmured. |
| ch056_s02 | 56 | 2 | Medical Training and the Initial Mildred Sequence | Initial Mildred Courtship | Jealousy Reversal and Date Arrangement | 862 | It was silly of him to take offence at what … as though she conferred a favour. Philip was vaguely irritated. |
| ch057_s01 | 57 | 1 | Medical Training and the Initial Mildred Sequence | Initial Mildred Courtship | The First Date as a Full Outing | 1155 | Philip arrived at Victoria Station nearly half an hour before … evening had been unsatisfactory. He felt irritated, restless, and miserable. |
| ch057_s02 | 57 | 2 | Medical Training and the Initial Mildred Sequence | Initial Mildred Courtship | Realization of Love and the Split from the Ideal | 783 | When he lay in bed he seemed still to see … was going to endure that ceaseless aching of his soul. |
| ch058_s01 | 58 | 1 | Medical Training and the Initial Mildred Sequence | First Rupture, Re-engagement, and Proposal Pressure | Morning Humiliation and Partial Repair | 617 | Philip woke early next morning, and his first thought was … “I don’t mind.” |
| ch058_s02 | 58 | 2 | Medical Training and the Initial Mildred Sequence | First Rupture, Re-engagement, and Proposal Pressure | Train Confidences and Rebuffed Kiss | 686 | He went out after tea and went back to his … She snatched away her hand and hurried towards her house. |
| ch058_s03 | 58 | 3 | Medical Training and the Initial Mildred Sequence | First Rupture, Re-engagement, and Proposal Pressure | Cancelled Date and Jealous Suspicion | 628 | Philip bought tickets for Saturday night. … He had an unhappy passion for certainty. |
| ch058_s04 | 58 | 4 | Medical Training and the Initial Mildred Sequence | First Rupture, Re-engagement, and Proposal Pressure | Surveillance, Confrontation, and the First Break | 477 | “What are you doing here?” … realised that she was glad to be quit of him. |
| ch059_s01 | 59 | 1 | Medical Training and the Initial Mildred Sequence | First Rupture, Re-engagement, and Proposal Pressure | Post Break Rumination and Renewed Fixation | 1392 | Philip passed the evening wretchedly. … out to Dunsford for Heaven’s sake to hold his tongue. |
| ch059_s02 | 59 | 2 | Medical Training and the Initial Mildred Sequence | First Rupture, Re-engagement, and Proposal Pressure | Failure Humiliation Self-Mortification | 679 | Then came the day of his examination. … to himself: “I must see her. I must see her.” |
| ch059_s03 | 59 | 3 | Medical Training and the Initial Mildred Sequence | First Rupture, Re-engagement, and Proposal Pressure | Humiliating Return and Re-engagement | 729 | The desire was so great that he could not give … her hand there and then to cover it with kisses. |
| ch060_s01 | 60 | 1 | Medical Training and the Initial Mildred Sequence | First Rupture, Re-engagement, and Proposal Pressure | Renewed Date and First Physical Access | 1112 | They dined in Soho. Philip was tremulous with joy... … ...“Mind my hat, silly. You are clumsy,” she said. |
| ch061_s01 | 61 | 1 | Medical Training and the Initial Mildred Sequence | First Rupture, Re-engagement, and Proposal Pressure | Routine Attachment and Limits on Access | 597 | He saw her then every day. He began going to … that he did not mind how common the aunt was. |
| ch061_s02 | 61 | 2 | Medical Training and the Initial Mildred Sequence | First Rupture, Re-engagement, and Proposal Pressure | Jealousy Crisis, Pursuit, and Reconciliation | 1033 | Their worst quarrel took place one evening at dinner when … little watch with a brooch to pin on her dress. |
| ch061_s03 | 61 | 3 | Medical Training and the Initial Mildred Sequence | First Rupture, Re-engagement, and Proposal Pressure | Rival Date and Status Anxiety | 468 | But three or four days later, when she brought him … make himself more interesting, he read industriously The Sporting Times. |
| ch062_s01 | 62 | 1 | Medical Training and the Initial Mildred Sequence | First Rupture, Re-engagement, and Proposal Pressure | Obsessive Torment and the Marriage Fantasy | 1372 | Philip did not surrender himself willingly to the passion that … for all the suffering I’ve endured,” he said to himself. |
| ch062_s02 | 62 | 2 | Medical Training and the Initial Mildred Sequence | First Rupture, Re-engagement, and Proposal Pressure | Proposal Rejection and Economic Humiliation | 706 | At last he could bear the agony no longer. After … dear, if you did you’d never speak to me again.” |
| ch063_s01 | 63 | 1 | Medical Training and the Initial Mildred Sequence | First Rupture, Re-engagement, and Proposal Pressure | Exam Failure, Self-Effacement, and False Hope | 902 | Philip did not pass the examination in anatomy at the … way of exception took a cigarette. She smoked very seldom. |
| ch063_s02 | 63 | 2 | Medical Training and the Initial Mildred Sequence | First Rupture, Re-engagement, and Proposal Pressure | Miller Engagement Revelation and Cutoff | 570 | “I don’t like to see a lady smoking,” she said. … sleep almost as soon as his head touched the pillow. |
| ch064_s01 | 64 | 1 | Medical Training and the Initial Mildred Sequence | Aftermath, Reorientation, and the Return of Meaning | Insomnia, Recoil, and the Wedding Gift | 440 | But about three in the morning Philip awoke and could … time indicate for himself the contempt he had for her. |
| ch064_s02 | 64 | 2 | Medical Training and the Initial Mildred Sequence | Aftermath, Reorientation, and the Return of Meaning | Hayward Reunion and the Reanimation of Beauty | 1329 | Philip had looked forward with apprehension to the day on … interesting,” laughed Philip. “Let’s go and have a stodgy tea.” |
| ch065_s01 | 65 | 1 | Medical Training and the Initial Mildred Sequence | Aftermath, Reorientation, and the Return of Meaning | Self Recovery and Meaning Line Return | 1322 | Hayward’s visit did Philip a great deal of good. … find out for myself, or else the answer meant nothing.” |
| ch066_s01 | 66 | 1 | The Norah Interlude and the Griffiths Triangle | Norah Repair and Partial Recovery | Norah's Entry and Relationship Formation | 1230 | Philip worked well and easily; he had a good deal … Philip of a happiness which seemed both solid and durable. |
| ch066_s02 | 66 | 2 | The Norah Interlude and the Griffiths Triangle | Norah Repair and Partial Recovery | Healing Attachment and Future Reorientation | 971 | They became lovers but remained friends. … I ever met who are able to learn from experience.” |
| ch067_s01 | 67 | 1 | The Norah Interlude and the Griffiths Triangle | Norah Repair and Partial Recovery | Return to London and the Hayward Value Contrast | 1015 | Philip looked forward to his return to London with impatience. … them back into their case, thought of them no more. |
| ch067_s02 | 67 | 2 | The Norah Interlude and the Griffiths Triangle | Norah Repair and Partial Recovery | Tavern Metaphysics and the Futility of Regret | 1207 | And it was Hayward who made a momentous discovery. … the forces of the universe were bent on spilling it.” |
| ch068_s01 | 68 | 1 | The Norah Interlude and the Griffiths Triangle | Griffiths Temptation, Mildred's Return, and the End of Norah | Illness, Nursing, and the Griffiths Bond | 1136 | One morning Philip on getting up felt his head swim, … He was in bed for five days. |
| ch068_s02 | 68 | 2 | The Norah Interlude and the Griffiths Triangle | Griffiths Temptation, Mildred's Return, and the End of Norah | Griffiths's Charisma, Social World, and Financial Temptation | 624 | Norah and Griffiths nursed him between them. Though Griffiths was … delightful it would be to make fifty pounds, so that... |
| ch069_s01 | 69 | 1 | The Norah Interlude and the Griffiths Triangle | Griffiths Temptation, Mildred's Return, and the End of Norah | Mildred's Return and the Reactivation of Rescue | 1374 | One afternoon, when he went back to his rooms from … ...but at all events it postponed it. |
| ch069_s02 | 69 | 2 | The Norah Interlude and the Griffiths Triangle | Griffiths Temptation, Mildred's Return, and the End of Norah | Betrayal Revelation, Jealousy, Disgust, and the Rejected Offer | 711 | Next day he wired again. Regret, unable to come. Will … ...“You are a good friend, Philip.” |
| ch069_s03 | 69 | 3 | The Norah Interlude and the Griffiths Triangle | Griffiths Temptation, Mildred's Return, and the End of Norah | Return to Companionship and the Caretaking Commitment | 836 | They went on talking, and soon they had returned to … hear her talking in this way. With her delicate li... |
| ch070_s01 | 70 | 1 | The Norah Interlude and the Griffiths Triangle | Griffiths Temptation, Mildred's Return, and the End of Norah | Norah Disengagement, Deception, and Emotional Withdrawal | 1420 | Philip expected to find a letter from Norah... … ...It delighted him to perform menial offices. |
| ch070_s02 | 70 | 2 | The Norah Interlude and the Griffiths Triangle | Griffiths Temptation, Mildred's Return, and the End of Norah | Mildred Domestic Enmeshment and the Break with Norah | 1263 | “You do spoil me,” she said... … an immense popularity among kitchen-maids. They think me so genteel.” |
| ch071_s01 | 71 | 1 | The Norah Interlude and the Griffiths Triangle | Griffiths Temptation, Mildred's Return, and the End of Norah | Griffiths's Advice Letter and the Execution of the Break | 1301 | Philip, in return for Griffiths’ confidences, had told him... … ...he leaned over her. |
| ch071_s02 | 71 | 2 | The Norah Interlude and the Griffiths Triangle | Griffiths Temptation, Mildred's Return, and the End of Norah | Norah's Wounded Confession, Truth Revelation, and Return to Mildred | 700 | “Won’t you drink a little? It’ll relieve you.” … his love for her by recollecting every whim she had. |
| ch072_s01 | 72 | 1 | The Norah Interlude and the Griffiths Triangle | Domestic Devotion, Griffiths Betrayal, and Sacrificial Collapse | Daily Devotion, Pregnancy Burden, and the Unwanted Child | 1015 | For the next three months Philip went every day to... … for all concerned such an event was to be desired. |
| ch072_s02 | 72 | 2 | The Norah Interlude and the Griffiths Triangle | Domestic Devotion, Griffiths Betrayal, and Sacrificial Collapse | Economic Claim, Self-Sacrifice, and Birth-Possession Fantasy | 1005 | “It’s all very fine to say this and that,” Mildred... … nurse entered. There was a slight smile on her lips. |
| ch073_s01 | 73 | 1 | The Norah Interlude and the Griffiths Triangle | Domestic Devotion, Griffiths Betrayal, and Sacrificial Collapse | Brighton Departure Letters and Separation Setup | 1079 | Three weeks later Philip saw Mildred and her baby off... … and to take her in his arms and kiss her. |
| ch073_s02 | 73 | 2 | The Norah Interlude and the Griffiths Triangle | Domestic Devotion, Griffiths Betrayal, and Sacrificial Collapse | Exam Success Weekend Plan and Deferred Return | 573 | He went into the examination with happy confidence. … superior lady-like person who was going to take the baby. |
| ch073_s03 | 73 | 3 | The Norah Interlude and the Griffiths Triangle | Domestic Devotion, Griffiths Betrayal, and Sacrificial Collapse | Brighton Reunion, Seafront Walk, and Lunch | 798 | Sunday. He blessed the day because it was fine. As... … blue sea, and the blue sea was trim and neat. |
| ch073_s04 | 73 | 4 | The Norah Interlude and the Griffiths Triangle | Domestic Devotion, Griffiths Betrayal, and Sacrificial Collapse | Baby Arrangement Brighton Display and Paris Projection | 630 | After luncheon they went to Hove to see the woman … he left her. He was strangely grotesque when he ran. |
| ch074_s01 | 74 | 1 | The Norah Interlude and the Griffiths Triangle | Domestic Devotion, Griffiths Betrayal, and Sacrificial Collapse | Saturday Idyll and the Open Transfer of Desire toward Griffiths | 947 | The following Saturday Mildred returned, and that eveni... … ... him,” she said. “All right,” he laughed. “Good-night.” |
| ch074_s02 | 74 | 2 | The Norah Interlude and the Griffiths Triangle | Domestic Devotion, Griffiths Betrayal, and Sacrificial Collapse | Triangular Evening, Surveillance, and Male Confrontation | 1348 | Next day, when they were having tea, Griffiths came in.... … a sigh of relief. The cab stopped at their door. |
| ch075_s01 | 75 | 1 | The Norah Interlude and the Griffiths Triangle | Domestic Devotion, Griffiths Betrayal, and Sacrificial Collapse | Love-Letter Revelation and the Initial Control Attempt | 1125 | Next day Philip was in a good temper. He was … were perfunctory, and she volunteered no remarks of her own. |
| ch075_s02 | 75 | 2 | The Norah Interlude and the Griffiths Triangle | Domestic Devotion, Griffiths Betrayal, and Sacrificial Collapse | Paris Collapse Restaurant Standoff and Financial Retaliation | 1019 | At last she interrupted abruptly what he was saying... … ...get home with.” Before she could speak he hurried away. |
| ch076_s01 | 76 | 1 | The Norah Interlude and the Griffiths Triangle | Domestic Devotion, Griffiths Betrayal, and Sacrificial Collapse | Mildred's Return, Panic, and Emotional Pressure | 1057 | Next day, in the afternoon, Philip sat in his room … shut her eyes as though she were going to faint. |
| ch076_s02 | 76 | 2 | The Norah Interlude and the Griffiths Triangle | Domestic Devotion, Griffiths Betrayal, and Sacrificial Collapse | Rescue Financing for Griffiths and Mildred's Elation | 486 | A strange idea came to Philip, and he spoke it … “You are a darling, Philip.” |
| ch076_s03 | 76 | 3 | The Norah Interlude and the Griffiths Triangle | Domestic Devotion, Griffiths Betrayal, and Sacrificial Collapse | Friday Return, Cold Negotiation, and the Final Advance for the Oxford Weekend | 1165 | She sent him a note a couple of hours later … She left him. |
| ch077_s01 | 77 | 1 | The Norah Interlude and the Griffiths Triangle | Domestic Devotion, Griffiths Betrayal, and Sacrificial Collapse | Waiting, Handover, Departure, and Immediate Collapse | 711 | After lunching in the basement of the Medical School Philip … could not; and great painful sobs were forced from him. |
| ch077_s02 | 77 | 2 | The Norah Interlude and the Griffiths Triangle | Domestic Devotion, Griffiths Betrayal, and Sacrificial Collapse | Ticket Burning, Night Wandering, and Self-Degradation | 758 | He got up at last, exhausted and ashamed, and washed … going to bed sank into a dreamless sleep till mid-day. |
| ch078_s01 | 78 | 1 | The Norah Interlude and the Griffiths Triangle | Withdrawal, Recovery, and the Norah Revisit | Waiting, Betrayal Confirmation, and Second Abandonment | 1384 | At last Monday came, and Philip thought his long torture … with himself. He felt that he was a little mad. |
| ch078_s02 | 78 | 2 | The Norah Interlude and the Griffiths Triangle | Withdrawal, Recovery, and the Norah Revisit | Blackstable Withdrawal, Reflective Recovery, and Exit Planning | 1099 | Since he was grown up Philip had been given the … no might to alter one smallest particle of what occurred. |
| ch079_s01 | 79 | 1 | The Norah Interlude and the Griffiths Triangle | Withdrawal, Recovery, and the Norah Revisit | New Rooms, Social Re-entry, and Norah Revaluation | 1247 | Philip went up to London a couple of days before … suffered would pass from his mind like a bad dream. |
| ch079_s02 | 79 | 2 | The Norah Interlude and the Griffiths Triangle | Withdrawal, Recovery, and the Norah Revisit | Visit to Norah, Rival Presence, and Awkward Waiting | 589 | But when next day, about tea-time, an hour at which … punishment, and with this thought he regained his good humour. |
| ch079_s03 | 79 | 3 | The Norah Interlude and the Griffiths Triangle | Withdrawal, Recovery, and the Norah Revisit | Private Confession, Engagement Shock, and Self-Recognition | 983 | At last, however, the clock struck six, and Kingsford got … have the gift of being amused at one’s own absurdity. |
| ch080_s01 | 80 | 1 | Clinical Humanism, Cronshaw, and the Athelny Threshold | Clinical Humanism and the Cronshaw Sequence | Medical Re-immersion and Clinical Training | 455 | For the next three months Philip worked on subjects which … from which he could extract a suggestion of human interest. |
| ch080_s02 | 80 | 2 | Clinical Humanism, Cronshaw, and the Athelny Threshold | Clinical Humanism and the Cronshaw Sequence | Griffiths's Mediation and Mildred's Aftermath History | 1278 | He saw Griffiths once in the distance, but, not to … into the vast anonymous mass of the population of London. |
| ch081_s01 | 81 | 1 | Clinical Humanism, Cronshaw, and the Athelny Threshold | Clinical Humanism and the Cronshaw Sequence | Outpatients Clerkship and the Clinical Frame | 1491 | At the beginning of the winter session Philip became an … bell and, when the porter poked his head in, said: |
| ch081_s02 | 81 | 2 | Clinical Humanism, Cronshaw, and the Athelny Threshold | Clinical Humanism and the Cronshaw Sequence | Women Patients, Poverty, and Fatal Social Reality | 1323 | “Old women, please.” He leaned back in his chair, chatting … it away. “I give him a year,” said Dr. Tyrell. |
| ch081_s03 | 81 | 3 | Clinical Humanism, Cronshaw, and the Athelny Threshold | Clinical Humanism and the Cronshaw Sequence | Comic Cases and the Total Impression of Life | 455 | Sometimes there was comedy. Now and then came a flash … nor bad there. There were just facts. It was life. |
| ch082_s01 | 82 | 1 | Clinical Humanism, Cronshaw, and the Athelny Threshold | Clinical Humanism and the Cronshaw Sequence | Cronshaw Recall, Mortality Conversation, and the Question of Meaning | 1359 | Towards the end of the year, when Philip was bringing … The answer is meaningless unless you discover it for yourself.” |
| ch083_s01 | 83 | 1 | Clinical Humanism, Cronshaw, and the Athelny Threshold | Clinical Humanism and the Cronshaw Sequence | Publication Hope and the Last Recognition Fantasy | 541 | Cronshaw was publishing his poems. His friends had been urging … delighted with the thought of the stir he would make. |
| ch083_s02 | 83 | 2 | Clinical Humanism, Cronshaw, and the Athelny Threshold | Clinical Humanism and the Cronshaw Sequence | Squalid Lodging Discovery, Rescue, and Cohabitation | 1305 | One day Philip went to dine by arrangement at the … supposed to have recovered from the avalanche of Christmas books. |
| ch084_s01 | 84 | 1 | Clinical Humanism, Cronshaw, and the Athelny Threshold | Clinical Humanism and the Cronshaw Sequence | Surgical Clinic, Deformity Exposure, and the Possibility of Operation | 1080 | At the new year Philip became dresser in the surgical … was rather a simple soul in those days,” he thought. |
| ch084_s02 | 84 | 2 | Clinical Humanism, Cronshaw, and the Athelny Threshold | Clinical Humanism and the Cronshaw Sequence | Cronshaw's Decline, the Upjohn Conflict, and the Tyrell Consultation | 1076 | Towards the end of February it was clear that Cronshaw … do any of the things Tyrell advised?” “Nothing,” smiled Cronshaw. |
| ch085_s01 | 85 | 1 | Clinical Humanism, Cronshaw, and the Athelny Threshold | Clinical Humanism and the Cronshaw Sequence | Discovery of Cronshaw's Death and Immediate Funeral Arrangements | 1230 | About a fortnight after this Philip, going home one evening … escaped them; they seemed to hurry just for hurrying’s sake. |
| ch085_s02 | 85 | 2 | Clinical Humanism, Cronshaw, and the Athelny Threshold | Clinical Humanism and the Cronshaw Sequence | Upjohn, Funeral Aestheticization, and Posthumous Critical Appropriation | 856 | Next morning Leonard Upjohn appeared with a small wreath of … a warm humanity about this article which was infinitely attractive. |
| ch086_s01 | 86 | 1 | Clinical Humanism, Cronshaw, and the Athelny Threshold | The Athelny Encounter and Household Entry | In-Patient Ward Framework and the First Encounter with Athelny | 806 | In the spring Philip, having finished his dressing in the … had finished his examination he went on to other beds. |
| ch086_s02 | 86 | 2 | Clinical Humanism, Cronshaw, and the Athelny Threshold | The Athelny Encounter and Household Entry | Athelny as Imaginative Stimulus through Spanish Poetry, Worldview, and Invitation | 804 | Thorpe Athelny’s illness was not grave, and, though remaining very … “I’d like to very much,” said Philip. |
| ch087_s01 | 87 | 1 | Clinical Humanism, Cronshaw, and the Athelny Threshold | The Athelny Encounter and Household Entry | House Arrival, Architectural Display, and the Threshold to Family Space | 626 | Ten days later Thorpe Athelny was well enough to leave … “what do you suppose the Almighty gave you fingers for?” |
| ch087_s02 | 87 | 2 | Clinical Humanism, Cronshaw, and the Athelny Threshold | The Athelny Encounter and Household Entry | Entry into the Dining Room and the Material Basis of the Athelny Household | 783 | They went upstairs, and Philip was taken into a room … never get him to sit down and eat his dinner.” |
| ch087_s03 | 87 | 3 | Clinical Humanism, Cronshaw, and the Athelny Threshold | The Athelny Encounter and Household Entry | Sunday Dinner, Domestic Philosophy, Sally, and Athelny's View of Marriage and Family | 767 | Athelny and Philip installed themselves in the great monkish chairs, … you must eat on Sundays roast beef and rice pudding.” |
| ch087_s04 | 87 | 4 | Clinical Humanism, Cronshaw, and the Athelny Threshold | The Athelny Encounter and Household Entry | Family-Tree Display and Philip's Suspicion of Athelny's Self-Mythologizing | 830 | “You’ll call when you’re ready for cheese,” said Sally impassively. … connection with the ancient family whose tree he was displaying. |
| ch088_s01 | 88 | 1 | Clinical Humanism, Cronshaw, and the Athelny Threshold | Aesthetic and Spiritual Pedagogy; Belonging | Betty Religion and Spain as Threshold to Alternative Worldview | 937 | There was a knock at the door and a troop … it is all finger-marked and frayed. Murillo is its painter.” |
| ch088_s02 | 88 | 2 | Clinical Humanism, Cronshaw, and the Athelny Threshold | Aesthetic and Spiritual Pedagogy; Belonging | El Greco Display and Athelny's Initial Toledo Lesson | 764 | Athelny got up from his chair, walked over to the … he is unastounded. His lips are not lips that smile. |
| ch088_s03 | 88 | 3 | Clinical Humanism, Cronshaw, and the Athelny Threshold | Aesthetic and Spiritual Pedagogy; Belonging | Philip's Internalization of Toledo and the Discovery of Inward Life | 1298 | Philip, silent still, returned to the photograph of Toledo, which … life of one who conquered realms and explored unknown lands. |
| ch089_s01 | 89 | 1 | Clinical Humanism, Cronshaw, and the Athelny Threshold | Aesthetic and Spiritual Pedagogy; Belonging | Family Tea, Mrs. Athelny, and First Inclusion | 985 | The conversation between Philip and Athelny was broken into by … “and it’s a charity to come and talk to him.” |
| ch089_s02 | 89 | 2 | Clinical Humanism, Cronshaw, and the Athelny Threshold | Aesthetic and Spiritual Pedagogy; Belonging | Repeated Sunday Integration with Children and Athelny Life History | 405 | On the following Saturday Philip received a postcard from Athelny … needs of his family had made him stick to it. |
| ch090_s01 | 90 | 1 | Mildred Cohabitation and Domestic Breakdown | Rescue, Cohabitation, and Unstable Domesticity | Piccadilly Sighting Solicitation Shock and Forced Private Talk | 769 | When he left the Athelnys’ Philip walked down Chancery Lane … like you to drive up to the door,” she said. |
| ch090_s02 | 90 | 2 | Mildred Cohabitation and Domestic Breakdown | Rescue, Cohabitation, and Unstable Domesticity | Hired Room Confession Disease and Money Gift | 765 | They were the first words either of them had spoken … he would be able to do nothing to prevent it. |
| ch090_s03 | 90 | 3 | Mildred Cohabitation and Domestic Breakdown | Rescue, Cohabitation, and Unstable Domesticity | Cohabitation Offer Terms and Domestic Reentry | 694 | She had got up to take the money, and they … he had to walk home, but it did not seem |
| ch091_s01 | 91 | 1 | Mildred Cohabitation and Domestic Breakdown | Rescue, Cohabitation, and Unstable Domesticity | Mildred Move in First Evening and Platonic Domestic Setup | 1362 | Next day he got up early to make the room … moving about in the bed-room, and in a little whil |
| ch092_s01 | 92 | 1 | Mildred Cohabitation and Domestic Breakdown | Rescue, Cohabitation, and Unstable Domesticity | Domestic Installation Tuesday Tavern and Midnight Boundary Test | 1188 | The following day was Tuesday. Philip as usual hurried through … He smiled when he heard her lock the door loudly. |
| ch092_s02 | 92 | 2 | Mildred Cohabitation and Domestic Breakdown | Rescue, Cohabitation, and Unstable Domesticity | Settled Cohabitation Music Hall Outing and Explicit Rejection | 1302 | The next few days passed without incident. Mildred settled down … She went out, slamming the door behind her. |
| ch093_s01 | 93 | 1 | Mildred Cohabitation and Domestic Breakdown | Rescue, Cohabitation, and Unstable Domesticity | Friendly Domestic Terms and Attachment to the Child | 999 | Next morning Mildred was sulky and taciturn. She remained in … known anybody else’s baby, so I can’t say,” said Philip. |
| ch093_s02 | 93 | 2 | Mildred Cohabitation and Domestic Breakdown | Rescue, Cohabitation, and Unstable Domesticity | Stock Windfall Operation Plan and Brighton Honeymoon Fantasy | 869 | Towards the end of his second term as in-patients’ clerk … “How much can I have for my new dress, Phil?” |
| ch094_s01 | 94 | 1 | Mildred Cohabitation and Domestic Breakdown | Brighton Erosion and Household Conflict | Operation Convalescence Brighton Entry and Separate Rooms Conflict | 1407 | Philip asked Mr. Jacobs, the assistant-surgeon for whom he had … “Oh, I wouldn’t fuss about that if I were you.” |
| ch094_s02 | 94 | 2 | Mildred Cohabitation and Domestic Breakdown | Brighton Erosion and Household Conflict | Brighton Boarding House Tedium and Domestic Stagnation | 878 | There were about a dozen people in the boarding-house. They … her dreams, which she would relate every day with prolixity. |
| ch094_s03 | 94 | 3 | Mildred Cohabitation and Domestic Breakdown | Brighton Erosion and Household Conflict | Athelny Letter and Future Release Longing | 997 | One morning he received a long letter from Thorpe Athelny. … was clean yesterday and just look at it now, Philip.” |
| ch095_s01 | 95 | 1 | Mildred Cohabitation and Domestic Breakdown | Brighton Erosion and Household Conflict | Surgical Dressing Accident Duty and Money Axis | 1042 | When they returned to London Philip began his dressing in … “I suppose money’s more important than love,” suggested Philip. |
| ch095_s02 | 95 | 2 | Mildred Cohabitation and Domestic Breakdown | Brighton Erosion and Household Conflict | Financial Erosion Job Failure Drawings Conflict and Baby Weaponization | 1076 | Money was in any case occupying Philip’s thoughts a good … angry. He grew used to having her about. Christmas came, |
| ch095_s03 | 95 | 3 | Mildred Cohabitation and Domestic Breakdown | Brighton Erosion and Household Conflict | Christmas Fire Conversation About Lost Love and Goodnight Kiss | 567 | “Where are you going to sit?” he asked Mildred. … She went to her bed-room and he began to read. |
| ch096_s01 | 96 | 1 | Mildred Cohabitation and Domestic Breakdown | Mildred's Countermove and the Final Rupture | Mildred Interior Baseline Contempt and Dependence | 632 | The climax came two or three weeks later. Mildred was … on another man’s child. He was peculiar and no mistake. |
| ch096_s02 | 96 | 2 | Mildred Cohabitation and Domestic Breakdown | Mildred's Countermove and the Final Rupture | Brighton Failure and Discovery of Lost Control | 805 | But one or two things surprised her. … in the same way for Philip to look at her. |
| ch096_s03 | 96 | 3 | Mildred Cohabitation and Domestic Breakdown | Mildred's Countermove and the Final Rupture | London Panic Dependency Fear and Sex as Last Weapon | 508 | When they got back to London Mildred began looking for … be independent of Philip; and she thought of the satisfactio |
| ch096_s04 | 96 | 4 | Mildred Cohabitation and Domestic Breakdown | Mildred's Countermove and the Final Rupture | Night Seduction Rejection and Narcissistic Rage | 1166 | One evening, at the beginning of February, Philip told her … “Cripple!” |
| ch097_s01 | 97 | 1 | Mildred Cohabitation and Domestic Breakdown | Mildred's Countermove and the Final Rupture | Morning after Shame Processing and Workday Displacement | 764 | Philip awoke with a start next morning, conscious that it … and Philip chaffed him as he put a clean dress |
| ch097_s02 | 97 | 2 | Mildred Cohabitation and Domestic Breakdown | Mildred's Countermove and the Final Rupture | Wrecked Rooms Persian Rug and Exit from Kennington | 1253 | He finished his day’s work and got on a tram … the damage done, and he had so little money lef |
| ch098_s01 | 98 | 1 | Poverty, Drapery, and Existential Reorientation | Speculation Crash and Starvation Crisis | War Boom Fantasy Speculative Entry and First Slide | 920 | And now it happened that the fortunes of Philip Carey, … to get over to the club reading-room and see t |
| ch098_s02 | 98 | 2 | Poverty, Drapery, and Existential Reorientation | Speculation Crash and Starvation Crisis | Hayward Departure Forced Liquidation and Shock Realisation | 966 | Early in April he went to the tavern in Beak … suppose anyone likes losing between three and four hundred pounds.” |
| ch098_s03 | 98 | 3 | Poverty, Drapery, and Existential Reorientation | Speculation Crash and Starvation Crisis | Seven Pounds Uncle Refusal and Panic Drop | 575 | When Philip got back to his shabby little room he … he had paid everything he would have seven pounds left |
| ch099_s01 | 99 | 1 | Poverty, Drapery, and Existential Reorientation | Speculation Crash and Starvation Crisis | Pawning Hunger and Futile Job Search | 749 | Philip began to pawn his clothes. He reduced his expenses … “I’d sooner starve,” Philip muttered to himself. |
| ch099_s02 | 99 | 2 | Poverty, Drapery, and Existential Reorientation | Speculation Crash and Starvation Crisis | Rent Crisis Mrs Higgins Offer and Near Tears | 430 | Once or twice the possibility of suicide presented itself to … clench his fists in order to prevent himself from crying. |
| ch100_s01 | 100 | 1 | Poverty, Drapery, and Existential Reorientation | Speculation Crash and Starvation Crisis | First Night Homelessness | 808 | Saturday. It was the day on which he had promised … did when they had no money. Presently he fell asleep. |
| ch100_s02 | 100 | 2 | Poverty, Drapery, and Existential Reorientation | Speculation Crash and Starvation Crisis | Job Search Humiliation and Borrowing | 1057 | When he awoke it was nearly mid-day, and he thought … Here you are. |
| ch100_s03 | 100 | 3 | Poverty, Drapery, and Existential Reorientation | Speculation Crash and Starvation Crisis | Fragmented Survival Second Night | 570 | Philip went to the public baths in Westminster and spent … and set out once more on the search for work. |
| ch100_s04 | 100 | 4 | Poverty, Drapery, and Existential Reorientation | Speculation Crash and Starvation Crisis | Sustained Deterioration and Limit | 721 | He went on in this way for several days. He … and a brush up in the lavatory at Charing Cross. |
| ch101_s01 | 101 | 1 | Poverty, Drapery, and Existential Reorientation | Speculation Crash and Starvation Crisis | Sunday Arrival Family Warmth and Hunger Concealment | 935 | When Philip rang a head was put out of the … to let the children come in till I call them.” |
| ch101_s02 | 101 | 2 | Poverty, Drapery, and Existential Reorientation | Speculation Crash and Starvation Crisis | Private Confrontation Confession and Move in Offer | 535 | Philip gave him a startled look, but before he could … not a very nice night to be out, is it?” |
| ch102_s01 | 102 | 1 | Poverty, Drapery, and Existential Reorientation | Drapery Initiation and Servitude | Athelny Job Lead Shopwalker Interview and Conditional Hire | 1164 | Athelny told Philip that he could easily get him something … send your box there on Monday.” The manager nodded: “Good-morning.” |
| ch103_s01 | 103 | 1 | Poverty, Drapery, and Existential Reorientation | Drapery Initiation and Servitude | First Day on the Floor Departmental Initiation and Canteen Shock | 729 | Mrs. Athelny lent Philip money to pay his landlady enough … kept in the ‘store’ and had their names written on. |
| ch103_s02 | 103 | 2 | Poverty, Drapery, and Existential Reorientation | Drapery Initiation and Servitude | Harrington Street Quarters Dormitory Culture and Burning Feet | 1380 | Philip was exhausted when work stopped at half past six. … fastened them with little pieces of stamp-paper he whistled monotonously. |
| ch104_s01 | 104 | 1 | Poverty, Drapery, and Existential Reorientation | Drapery Initiation and Servitude | Social Evening Basement Performance and Claustrophobic Loneliness | 1627 | The social evenings took place on alternate Mondays. There was … the life he was leading. The soldier was snoring quietly. |
| ch105_s01 | 105 | 1 | Poverty, Drapery, and Existential Reorientation | Drapery Initiation and Servitude | Pay Day and Athelny Respite | 830 | The wages were paid once a month by the secretary. … go back to Harrington Street without a feeling of exultation. |
| ch105_s02 | 105 | 2 | Poverty, Drapery, and Existential Reorientation | Drapery Initiation and Servitude | Dark Return and Exit Fantasy | 650 | At first Philip, in order not to forget what he … became unendurable, he had at all events a way out. |
| ch105_s03 | 105 | 3 | Poverty, Drapery, and Existential Reorientation | Drapery Initiation and Servitude | Duty Week Window Dressing Shame and Partial Social Fit | 796 | “Second to the right, madam, and down the stairs. First … time; and he was nearly always tired and often hungry. |
| ch106_s01 | 106 | 1 | Poverty, Drapery, and Existential Reorientation | Existential Reorientation within Drapery | Lawson Encounter Shame Exposure and Hayward Death Shock | 1280 | Philip avoided the places he had known in happier times. … fox or the goat. Human beings filled him with disgust. |
| ch106_s02 | 106 | 2 | Poverty, Drapery, and Existential Reorientation | Existential Reorientation within Drapery | Mortality Reflection and Question | 756 | But presently the influence of the place descended upon him. … ...for nothing was there a why and a wherefore. |
| ch106_s03 | 106 | 3 | Poverty, Drapery, and Existential Reorientation | Existential Reorientation within Drapery | Persian Rug Epiphany | 1038 | Thinking of Cronshaw, Philip remembered the Persian rug which he … Philip was happy. |
| ch107_s01 | 107 | 1 | Poverty, Drapery, and Existential Reorientation | Existential Reorientation within Drapery | Department Patronage and Creative Promise | 596 | Mr. Sampson, the buyer, took a fancy to Philip. Mr. … it comes round to my own idea in the end.” |
| ch107_s02 | 107 | 2 | Poverty, Drapery, and Existential Reorientation | Existential Reorientation within Drapery | Alice Antonia Commission and Tivoli Validation | 966 | One day, when Philip had been at the shop for … had attached himself to Philip, could not conceal his bitterness. |
| ch107_s03 | 107 | 3 | Poverty, Drapery, and Existential Reorientation | Existential Reorientation within Drapery | Wage Stagnation Raise Risk and Queue of Replacements | 420 | “Some people ’ave all the luck,” he said. “You’ll be … employment. It made him shudder. He dared not risk it. |
| ch108_s01 | 108 | 1 | Uncle Carey's Decline and Clinical Return | Uncle Carey's Decline, Inheritance, and Return | Letter Raise and Return to Blackstable | 564 | The winter passed. Now and then Philip went to the … long he would have to wait for that. Two years? |
| ch108_s02 | 108 | 2 | Uncle Carey's Decline and Clinical Return | Uncle Carey's Decline, Inheritance, and Return | Direct Observation of Uncle Decay | 561 | He was startled at the change in his uncle. … ...It was a hideous old age. |
| ch108_s03 | 108 | 3 | Uncle Carey's Decline and Clinical Return | Uncle Carey's Decline, Inheritance, and Return | Dr Wigram Consultation and Death Fear Judgment | 878 | In the afternoon Dr. Wigram came, and after the visit … nakedness the dreadful dismay of the unknown which he suspected. |
| ch108_s04 | 108 | 4 | Uncle Carey's Decline and Clinical Return | Uncle Carey's Decline, Inheritance, and Return | Athelny Household Labour Philosophy and Meaninglessness Insight | 1066 | The fortnight passed quickly and Philip returned to London. He … and he faced it with a strange sense of power. |
| ch109_s01 | 109 | 1 | Uncle Carey's Decline and Clinical Return | Uncle Carey's Decline, Inheritance, and Return | Mildred Letter and First Visit | 1004 | The autumn passed into winter. Philip had left his address … in a rash, and I can’t get rid of it.” |
| ch109_s02 | 109 | 2 | Uncle Carey's Decline and Clinical Return | Uncle Carey's Decline, Inheritance, and Return | Diagnosis Fear Bonding and Cheap Dinner | 844 | Philip felt a twinge of horror in his heart. … to go back to Harrington Street. He was hideously bored. |
| ch109_s03 | 109 | 3 | Uncle Carey's Decline and Clinical Return | Uncle Carey's Decline, Inheritance, and Return | Daily Visits Suspicion and Final Theatre Break | 829 | Philip went to see her every day. She took the … That was the end. He did not see her again. |
| ch110_s01 | 110 | 1 | Uncle Carey's Decline and Clinical Return | Uncle Carey's Decline, Inheritance, and Return | Christmas Visit Uncle Carey Deterioration and Death Horror Observation | 1048 | Christmas that year falling on Thursday, the shop was to … that there was no God and after this life nothing. |
| ch110_s02 | 110 | 2 | Uncle Carey's Decline and Clinical Return | Uncle Carey's Decline, Inheritance, and Return | Boxing Day Inheritance Calculation Euthanasia Temptation and Uncanny Recognition | 876 | On the evening of Boxing Day Philip sat in the … “Yes, she married a widower. I believe they’re quite comfortable.” |
| ch111_s01 | 111 | 1 | Uncle Carey's Decline and Clinical Return | Uncle Carey's Decline, Inheritance, and Return | Long Wait Shop Exit Return to Blackstable and False Rally | 1040 | Next day Philip began work again, but the end which … whether the next vicar will suit us half so well.” |
| ch111_s02 | 111 | 2 | Uncle Carey's Decline and Clinical Return | Uncle Carey's Decline, Inheritance, and Return | Night Vigil Last Terror and Human Clinging | 681 | For several days Mr. Carey continued without change. His appetite … and dewy in the morning. The birds were singing gaily. |
| ch111_s03 | 111 | 3 | Uncle Carey's Decline and Clinical Return | Uncle Carey's Decline, Inheritance, and Return | Dawn Release Communion and Death | 753 | The sky was blue, but the air, salt-laden, was sweet … run down. The bluebottle buzzed, buzzed noisily against the windowpane. |
| ch112_s01 | 112 | 1 | Uncle Carey's Decline and Clinical Return | Uncle Carey's Decline, Inheritance, and Return | Funeral Aftermath Inheritance and Mother Letter Discovery | 978 | Josiah Graves in his masterful way made arrangements, becoming but … gentle soul. He went on with the Vicar's dreary correspondence. |
| ch112_s02 | 112 | 2 | Uncle Carey's Decline and Clinical Return | Uncle Carey's Decline, Inheritance, and Return | Hospital Reentry School Revisit and Pattern of Life Reassertion | 1235 | A few days later he went up to London, and … saw the beautiful thing before him only with his eyes. |
| ch113_s01 | 113 | 1 | Uncle Carey's Decline and Clinical Return | District Medicine and Social Vision | District Midwifery Concrete Cases and Unwanted Births | 712 | At the beginning of the last week in August Philip … little 'earts," said the grandmother, "what should 'appen to them?" |
| ch113_s02 | 113 | 2 | Uncle Carey's Decline and Clinical Return | District Medicine and Social Vision | District Midwifery Social Reflection Work and Poverty | 948 | The great difficulty was to keep the mothers in bed … get no time now not even to read the paper.' |
| ch113_s03 | 113 | 3 | Uncle Carey's Decline and Clinical Return | District Medicine and Social Vision | Herb and Polly Dinner and Working Class Domestic Warmth | 823 | The usual practice was to pay three visits after a … "'Ow d'you know there's going to be a next time?" |
| ch114_s01 | 114 | 1 | Uncle Carey's Decline and Clinical Return | District Medicine and Social Vision | Last Call Initial Examination and Teen Mother Crisis | 893 | The three weeks which the appointment lasted drew to an … he said. He looked at her quickly: something had happened. |
| ch114_s02 | 114 | 2 | Uncle Carey's Decline and Clinical Return | District Medicine and Social Vision | Senior Intervention Husband Confrontation and Death | 659 | In cases of emergency the S. O. C.—senior obstetric clerk—had … have you had?” “Sixty-three.” “Good. You’ll get your certificate then.” |
| ch114_s03 | 114 | 3 | Uncle Carey's Decline and Clinical Return | District Medicine and Social Vision | Dawn Bridge Vision Social Fatalism and Beauty against Misery | 573 | They arrived at the hospital, and the S. O. C. … beauty of the world. Beside that nothing seemed to matter. |
| ch115_s01 | 115 | 1 | Uncle Carey's Decline and Clinical Return | Sally within the Athelny Household | Sally Portrait Necklace Boundary and Quiet Parlour Intimacy | 804 | Philip spent the few weeks that remained before the beginning … their flat chests and anaemic faces. Mildred suffered from anaemia. |
| ch115_s02 | 115 | 2 | Uncle Carey's Decline and Clinical Return | Sally within the Athelny Household | Suitor Tea Family Pressure and Sally Refusal | 1044 | After a time it appeared that Sally had a suitor. … touch her sense of humour. She was an odd girl. |
| ch116_s01 | 116 | 1 | Doctor South, Sally Courtship, and the Pregnancy Crisis | Doctor South Apprenticeship and the Alternative Future | Last Year Balance Diploma and Farnley Locum Setup | 985 | During his last year at St. Luke’s Philip had to … speak; he merely stared at Philip. Philip was taken aback. |
| ch116_s02 | 116 | 2 | Doctor South, Sally Courtship, and the Pregnancy Crisis | Doctor South Apprenticeship and the Alternative Future | Doctor South Arrival First Dinner and Mutual Sizing Up | 799 | “I think you’re expecting me,” he said. “The secretary of … left the room. “What a funny old fellow!” thought Philip. |
| ch116_s03 | 116 | 3 | Doctor South, Sally Courtship, and the Pregnancy Crisis | Doctor South Apprenticeship and the Alternative Future | Farnley Practice Comic Sparring and Professional Ease | 751 | He soon discovered why Doctor South’s assistants found it difficult … “Damn his impudence,” he chuckled to himself. “Damn his impudence.” |
| ch117_s01 | 117 | 1 | Doctor South, Sally Courtship, and the Pregnancy Crisis | Doctor South Apprenticeship and the Alternative Future | Hop Field Invitation Farnley Idyll and Coastal Reverie | 431 | Philip had written to Athelny to tell him that he … heart and at the same time melted it to tenderness. |
| ch117_s02 | 117 | 2 | Doctor South, Sally Courtship, and the Pregnancy Crisis | Doctor South Apprenticeship and the Alternative Future | Mrs Fletcher Call Sunset and Partnership Offer | 1225 | One evening, when Philip had reached his last week with … He appeared to look upon Philip’s reasons as sound. |
| ch117_s03 | 117 | 3 | Doctor South, Sally Courtship, and the Pregnancy Crisis | Doctor South Apprenticeship and the Alternative Future | Doctor South Self Revelation and Station Farewell | 463 | Dropping the subject, he began to talk of his own … his empty house. He felt very old and very lonely. |
| ch118_s01 | 118 | 1 | Doctor South, Sally Courtship, and the Pregnancy Crisis | Hop-Field Courtship with Sally | Ferne Arrival Firelight Sally and Rural Family Enclosure | 1313 | It was late in the evening when Philip arrived at … wake me I’ll come and bathe with them,” said Philip. |
| ch118_s02 | 118 | 2 | Doctor South, Sally Courtship, and the Pregnancy Crisis | Hop-Field Courtship with Sally | Dawn Bath Meadow Crossing and Communal Morning Entry | 499 | Jane and Harold and Edward shouted with delight at the … meadow into the hop-field. They were the last to leave. |
| ch118_s03 | 118 | 3 | Doctor South, Sally Courtship, and the Pregnancy Crisis | Hop-Field Courtship with Sally | Hop Field Labour World Aesthetic Immersion and Earn My Dinner | 903 | A hop-garden was one of the sights connected with Philip’s … the hand, as he strolled away. “No work, no dinner.” |
| ch119_s01 | 119 | 1 | Doctor South, Sally Courtship, and the Pregnancy Crisis | Hop-Field Courtship with Sally | First Full Picking Day Communal Rhythm and Sally in Work Light | 1239 | Philip had not a basket of his own, but sat … presently Philip heard his wife address the world in general. |
| ch119_s02 | 119 | 2 | Doctor South, Sally Courtship, and the Pregnancy Crisis | Hop-Field Courtship with Sally | Tea Errand Night Walk and First Kiss | 825 | “There now, I’m out of tea and I wanted Athelny … He drew her into the darker shadow of the hedge. |
| ch120_s01 | 120 | 1 | Doctor South, Sally Courtship, and the Pregnancy Crisis | Hop-Field Courtship with Sally | Morning after Bewilderment and Sallys Maternal Care | 888 | Philip slept like a log and awoke with a start … as she had with regard to her brothers and sisters. |
| ch120_s02 | 120 | 2 | Doctor South, Sally Courtship, and the Pregnancy Crisis | Hop-Field Courtship with Sally | Evening Clarification Mutual Choice and Night Tryst | 930 | It was not till the evening that he found himself … “You’re an old silly, that’s what you are,” she said. |
| ch121_s01 | 121 | 1 | Doctor South, Sally Courtship, and the Pregnancy Crisis | Pregnancy Shock and Sacrificial Commitment | Hospital Routine and Quiet Sally Companionship | 560 | When the hops were picked, Philip with the news in … him always with admiring awe. She made him feel unworthy. |
| ch121_s02 | 121 | 2 | Doctor South, Sally Courtship, and the Pregnancy Crisis | Pregnancy Shock and Sacrificial Commitment | Pregnancy Shock Panic and Rationalized Escape | 1267 | Then, one day, about three weeks after they had come … would never allow himself to be turned from his purpose. |
| ch121_s03 | 121 | 3 | Doctor South, Sally Courtship, and the Pregnancy Crisis | Pregnancy Shock and Sacrificial Commitment | Decision to Marry and Sacrificial Elation | 838 | Philip said all this to himself, but he knew he … memory: Forgive them, for they know not what they do. |
| ch122_s01 | 122 | 1 | Release, Choice, and Settlement | Residual Bondage, Recognition, and Settlement | Residual Bondage Trigger False Alarm and Freedom Shock | 830 | He had arranged to meet Sally on Saturday in the … not sure,” he muttered. “You are funny. Most men would.” |
| ch122_s02 | 122 | 2 | Release, Choice, and Settlement | Residual Bondage, Recognition, and Settlement | Recognition of True Desire Proposal and Settlement | 533 | He realised that he had deceived himself; it was no … passed, hastening in every direction, and the sun was shining. |
