# Supplementary material for: Mapping relational mechanism clusters in Of Human Bondage: a theory-driven multiscale embedding analysis
Source: Front Psychol. 2026 Jul 16;17:1836153. doi: 10.3389/fpsyg.2026.1836153 (PMC13420877; doi:10.3389/fpsyg.2026.1836153)
Supplement: Supplementary file 5 [file Table_3.DOCX]

Supplementary Appendix S3

# Aggregation Robustness and Cross-Model Rank Agreement (Full Tables)

This appendix expands the robustness analyses summarized in Sections 3.6 and 3.7 of the manuscript. Part A reports the chapter-level focal group contrast (Test 2: Mildred Arc, n = 40, vs. Resolution, n = 13) under four aggregation schemes; Part B reports the chapter-level lag-1 autocorrelation (Test 3) on the full 122-chapter sequence under the same schemes; Part C reports cross-model rank agreement of 165-scene construct profiles between the primary Qwen3-Embedding-8B encoder and the two comparison encoders (MiniLM, Qwen3-Embedding-0.6B).

*Aggregation schemes (each operates on the cached Qwen3-8B chunk and variant embeddings; no model re-encoding was required):*

- **VEC:** Published pipeline. Chunks → per-dimension trimmed mean (trim_frac = 0.1) → L2-normalize → cosine to construct embedding (itself the mean of four unit-normalized variant vectors).
- **SL:** Similarity-level anchor aggregation. Cosine similarity to each unit-normalized variant vector, then mean of the four similarities. Equivalent to VEC up to a positive per-construct scalar (||v̄|| ≤ 1); rank orderings, standardized effect sizes, and permutation p-values are identical to VEC.
- **MEAN:** Plain-mean chunk pooling. Chunks → plain mean → L2-normalize → cosine to construct embedding.
- **PAIR:** Pairwise chunk–anchor similarity. Cosine similarity between each chunk and the construct embedding, then mean of the chunk-level similarities (chapter score = mean of cos(chunk, construct) across the chapter's chunks).

## Part A. Test 2 (Mildred Arc vs. Resolution): Hedges' g and BH-FDR q under four aggregation schemes

Permutation-based mean-difference tests (seed = 42, 10,000 permutations); Benjamini–Hochberg FDR correction across the eight focal constructs in each scheme. * indicates q < 0.05 (FDR-significant). Negative controls are reported below the positive constructs but were not included in the FDR correction within each scheme. The same four focal constructs (NRS, Coercive Control, Intermittent Reinforcement, Trauma Bonding) survive FDR correction under all four schemes.

| Construct | VEC g | VEC q | SL g | SL q | MEAN g | MEAN q | PAIR g | PAIR q |
| --- | --- | --- | --- | --- | --- | --- | --- | --- |
| Narcissistic Relational Style (NRS) | +1.126 | 0.0088* | +1.126 | 0.0088* | +1.112 | 0.0096* | +1.185 | 0.0048* |
| Coercive Control | +0.937 | 0.0211* | +0.937 | 0.0211* | +0.924 | 0.0208* | +0.995 | 0.0100* |
| Intermittent Reinforcement | +0.809 | 0.0268* | +0.809 | 0.0268* | +0.788 | 0.0284* | +0.915 | 0.0122* |
| Repeated-Investment Logic | +0.603 | 0.0918 | +0.603 | 0.0918 | +0.595 | 0.1014 | +0.691 | 0.0531 |
| Trauma Bonding | +0.871 | 0.0211* | +0.871 | 0.0211* | +0.860 | 0.0208* | +0.901 | 0.0122* |
| Learned Helplessness | +0.367 | 0.2845 | +0.367 | 0.2845 | +0.361 | 0.2961 | +0.436 | 0.1970 |
| Relational Warmth | +0.508 | 0.1497 | +0.508 | 0.1497 | +0.499 | 0.1549 | +0.535 | 0.1277 |
| Existential Patterning | -0.137 | 0.6607 | -0.137 | 0.6607 | -0.159 | 0.6152 | -0.111 | 0.7273 |
| Mathematical Reasoning [NEG] | -0.204 | 0.5230 | -0.204 | 0.5230 | -0.220 | — | -0.173 | — |
| Geography/Navigation [NEG] | -0.708 | 0.0594 | -0.708 | 0.0594 | -0.708 | — | -0.714 | — |

## Part B. Test 3 (lag-1 autocorrelation, full-122): r₁ and BH-FDR q under four aggregation schemes

Permutation-based lag-1 autocorrelation tests on the full 122-chapter sequence (seed = 42, 10,000 permutations); BH-FDR correction across the eight focal constructs. All eight focal constructs remain FDR-significantly autocorrelated under every aggregation scheme.

| Construct | VEC r₁ | VEC q | SL r₁ | SL q | MEAN r₁ | MEAN q | PAIR r₁ | PAIR q |
| --- | --- | --- | --- | --- | --- | --- | --- | --- |
| Narcissistic Relational Style (NRS) | +0.441 | 0.0001* | +0.441 | 0.0001* | +0.446 | 0.0001* | +0.420 | 0.0001* |
| Coercive Control | +0.391 | 0.0001* | +0.391 | 0.0001* | +0.388 | 0.0001* | +0.365 | 0.0001* |
| Intermittent Reinforcement | +0.455 | 0.0001* | +0.455 | 0.0001* | +0.459 | 0.0001* | +0.456 | 0.0001* |
| Repeated-Investment Logic | +0.395 | 0.0001* | +0.395 | 0.0001* | +0.401 | 0.0001* | +0.428 | 0.0001* |
| Trauma Bonding | +0.484 | 0.0001* | +0.484 | 0.0001* | +0.484 | 0.0001* | +0.470 | 0.0001* |
| Learned Helplessness | +0.359 | 0.0001* | +0.359 | 0.0001* | +0.356 | 0.0001* | +0.380 | 0.0001* |
| Relational Warmth | +0.486 | 0.0001* | +0.486 | 0.0001* | +0.491 | 0.0001* | +0.478 | 0.0001* |
| Existential Patterning | +0.191 | 0.0142* | +0.191 | 0.0142* | +0.192 | 0.0319* | +0.184 | 0.0380* |

## Part C. Cross-model 165-scene rank agreement (Spearman)

Per-construct Spearman ρ between the primary Qwen3-Embedding-8B scene-level construct profile and each of the two comparison encoders, across the 165 hand-segmented focal scenes (Chs. 55–122). The within-family contrast (8B vs. 0.6B) shows higher mean agreement than the cross-family contrast (8B vs. MiniLM), but both contrasts show positive agreement for every construct and every negative control.

| Construct | 8B ~ MiniLM ρ | 8B ~ 0.6B ρ |
| --- | --- | --- |
| Narcissistic Relational Style (NRS) | +0.475 | +0.805 |
| Coercive Control | +0.417 | +0.528 |
| Intermittent Reinforcement | +0.497 | +0.746 |
| Repeated-Investment Logic | +0.608 | +0.758 |
| Trauma Bonding | +0.545 | +0.785 |
| Learned Helplessness | +0.699 | +0.831 |
| Relational Warmth | +0.718 | +0.690 |
| Existential Patterning | +0.629 | +0.699 |
| Mean (positive constructs) | +0.574 | +0.730 |
| Mathematical Reasoning [NEG] | +0.340 | +0.548 |
| Geography/Navigation [NEG] | +0.635 | +0.602 |

## Notes on chunking and trimming

At the chapter scale, chunk counts ranged from 3 to 21 per chapter (median = 11, mean = 11.66, total = 1422 chunks across 122 chapters); because floor(0.1 · n) = 0 whenever n < 10, trimming engages only for the 88 chapters with ≥ 10 chunks (trimming at most two chunks per tail) and reduces exactly to plain-mean pooling for the remaining 34 chapters. At the scene scale, every scene contained 2–6 chunks (median = 3), so floor(0.1 · n) = 0 for all 165 scenes and the trimmed mean is exactly equal to the ordinary mean at scene scale.

*Sanity check: re-aggregating the cached chunk embeddings under the published VEC scheme reproduces the released chapter-level similarities to within 8.94e-08 (max absolute difference across all 122 chapters and eight constructs), confirming that the cached pipeline is byte-faithful to the published outputs.*
